# Organophotoredox 1,6-Addition of 3,4-Dihydroquinoxalin-2-ones to *para*-Quinone Methides Using Visible Light

**DOI:** 10.1021/acsorginorgau.2c00064

**Published:** 2023-01-20

**Authors:** Jaume Rostoll-Berenguer, Víctor García-García, Gonzalo Blay, José R. Pedro, Carlos Vila

**Affiliations:** † Departament de Química Orgànica, Facultat de Química, Universita de València, Dr. Moliner 50, 46100 Burjassot, València, Spain

**Keywords:** organophotoredox catalysis, visible-light photocatalysis, quinoxalin-2-ones, 1,6-addition, para-quinone methides

## Abstract

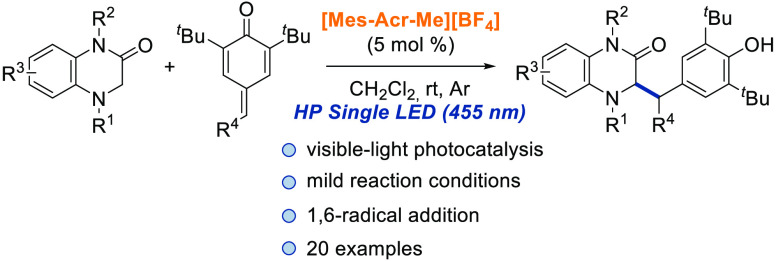

An organophotoredox 1,6-radical addition of 3,4-dihidroquinoxalin-2-ones to *para*-quinone methides catalyzed by Fukuzumi’s photocatalyst is described under the irradiation of a HP Single LED (455 nm). The corresponding 1,1-diaryl compounds bearing a dihydroquinoxalin-2-one moiety (20 examples) are obtained with good to excellent yields under mild reaction conditions. Several experiments have been carried out in order to propose a reaction mechanism.

The conjugate addition of nucleophiles to electron-deficient alkenes is one of the most important synthetic methodologies for the formation of C–C bonds in organic synthesis.^1−3^ In contrast, the radical addition (Giese reaction) to electron-deficient alkenes is less investigated.^4−6^ In this context, the 1,6-addition^7−9^ is much less studied than the 1,4-addition that is pivotal for synthetic organic chemistry. Nevertheless, in recent years, *para*-quinone methides have become important substrates for the development of 1,6-conjugate additions.^10−12^*para*-Quinone methides are organic molecules that contain a carbonyl group and an *exo*-methylene moiety connected to cyclohexadiene, and display intrinsically high reactivity as versatile Michael acceptors driven by aromatization. Despite the significant advances in the field of 1,6-conjugate additions thanks to the versatility of *para*-quinone methides, if we compare the nucleophilic versus the radical 1,6-addition reactions, we could conclude that the radical version is scarcely explored.

Since the development of visible-light photoredox catalysis has allowed the generation of organic radicals under mild reaction conditions,^13−17^ impressive achievements have been made in radical functionalization reactions. Accordingly, several radical 1,6-additions have been reported using *para*-quinone methides as electron-deficient acceptors mediated by visible-light.^18,19^ For example, photocatalytic fluoroalkylation reactions using sodium sulfinates^20^ or difluoroalkylating reagents^21^ have been described, as well as alkylation reactions using cyanoalkylation reagents,^22^ 4-substituted Hantzsch esters,^23^ or carboxylic acids.^24−27^ Moreover, a photocatalytic 1,6-radical acylation reaction had been reported using simple carboxylic acids, triphenylphospine, and iridium photocatalyst.^28^

Regarding the rich chemistry of α-aminoradicals^29,30^ for conjugate additions, amines such as glycine^26^ or anilines^31^ have been used as precursors to describe the radical 1,6-addition with *para*-quinone methides. These reactions represent a convenient strategy for the synthesis of 2,2-diarylethylamines,^32^ an important motif that widely exists in drugs and natural products. Despite these successful examples, these reports are limited to acyclic amines. As a part of our continuing interest in the development of synthetic approaches for the generation of α-amino radicals from other tertiary amines such as 3,4-dihydroquinoxalin-2-ones,^33−39^ we envisioned that these cyclic amines could be suitable α-amino radical precursors which undergo a 1,6-radical addition with *para*-quinone methides using photocatalysis (Scheme 1). Furthermore, 1,4-dihydroquinoxalinones are an interesting class of nitrogen heterocycles which are present in many molecules with biological activities such as antiviral,^40^ anticancer^41^ or anti-inflammatory compounds.^42^ Accordingly, the functionalization of this class of nitrogen heterocycles is significant for medicinal and pharmaceutical chemistry.

**Scheme 1:**
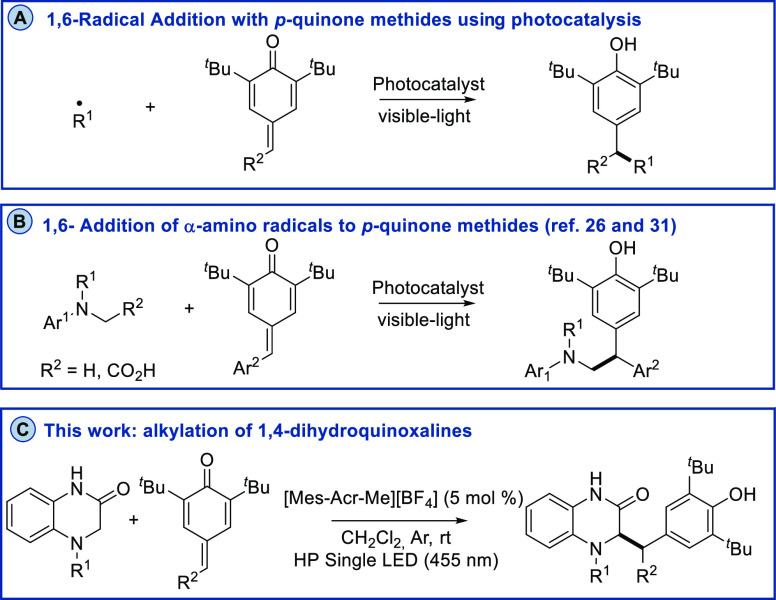
(A) 1,6-Radical addition with *para*-quinone methides. (B) 1,6-Addition of α-amino radicals to *para*-quinone methides. (C) 1,6-Radical addition of dihydroquinoxalin-2-ones.

Our previous observations in this field^35,36^ prompted us to start the optimization of the reaction between 4-benzyl-3,4-dihydroquinoxalin-2(1*H*)-one (**1a**) and 4-benzylidene-2,6-di-*tert*-butylcyclohexa-2,5-dien-1-one (**2a**) focusing on the photoredox catalyst. Specifically, we decided to screen several photoredox catalysts while using dry and degassed MeCN as solvent, 0.15 mmol of **1a**, 0.1 mmol of **2a** and HP (High Power) Single LED (455 nm) as light source (Table 1).

**Table 1:**
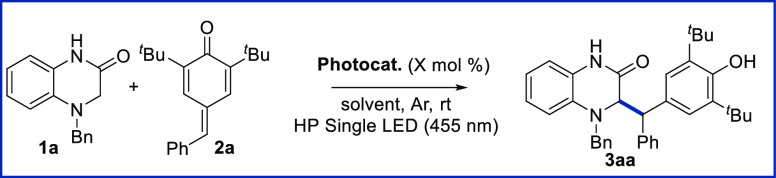
Optimization of the Reaction Conditions^a^

entry	photocatalyst (X mol %)	solvent	*t* (h)	dr^b^	yield (%)^c^
1	Ru(bpy)_3_Cl_2_ (1%)	CH_3_CN	24	1.2:1	72
2	Eosin–Y-Na_2_ (5%)	CH_3_CN	24	1.1:1	27
3	[2,4,6-Ph_3_-pyrillium][BF_4_] (5%)	CH_3_CN	19	1.1:1	43
4	4-CzIPN (5%)	CH_3_CN	24	–	–i
5	9,10-phenanthrenedione (5%)	CH_3_CN	19	–	–
6	[Mes-Acr-Me][BF_4_] (5%)	CH_3_CN	19	1.3:1	94
7	[Mes-Acr-Me][BF_4_] (5%)	DMF	24	1.9:1	41
8	[Mes-Acr-Me][BF_4_] (5%)	CH_2_Cl_2_	9	1.2:1	99 (99)^d^
9	[Mes-Acr-Me][BF_4_] (5%)	toluene	26	–	–
10	[Mes-Acr-Me][BF_4_] (5%)	DCE	6	1.3:1	93
11	[Mes-Acr-Me][BF_4_] (5%)	CHCl_3_	8	1:1	87
12^e^	[Mes-Acr-Me][BF_4_] (5%)	CH_2_Cl_2_	9	1.2:1	71
13^f^	[Mes-Acr-Me][BF_4_] (5%)	CH_2_Cl_2_	9	1.4:1	60
14	–	CH_2_Cl_2_	9	–	–
15^g^	[Mes-Acr-Me][BF_4_] (5%)	CH_2_Cl_2_	9	–	–
16^h^	[Mes-Acr-Me][BF_4_] (5%)	CH_2_Cl_2_	9	–	–i
17^j^	[Mes-Acr-Me][BF_4_] (5%)	CH_2_Cl_2_	24	–	–

a Reaction conditions: **1a** (0.15 mmol), **2a** (0.1 mmol), *X* mol % of photocatalyst in 1 mL of solvent at rt under argon atmosphere and HP Single LED (455 nm) irradiation.

b Determined by ^1^H NMR.

c Yield determined by ^1^H NMR using *p*-acetophenone as internal standard.

d In brackets isolated yield after column chromatography using Et_3_N-deactivated silica gel.

e 0.12 mmol of **1a** was used.

f 0.1 mmol of **1a** and 0.12 mmol of **2a** were used.

g Reaction performed under darkness.

h Reaction performed under air atmosphere.

i Complex reaction mixture.

j 1.5 equiv of TEMPO were added.

First, we evaluated the reaction using Ru(bpy)_3_Cl_2_ as photocatalyst (entry 1). With these conditions, we obtained product **3aa** in 72% yield determined as a mixture of diastereoisomers (1.2:1). After we decided to evaluate organophotocatalysts in order to increase the yield of product **3aa**. When Eosin Y (entry 2) or 2,4,6-triphenylpyrylium tetrafluoroborate (entry 3) were used as photocatalysts, the efficiency of the reaction was worse, and **3aa** was gained with much lower yield. A complex reaction mixture was observed when 4-CzIPN (2,4,5,6-tetrakis(9*H*-carbazol-9-yl) isophthalonitrile)^43^ was used, while product **3aa** was not observed when 9,10-phenanthrenedione^44,45^ was tested (entry 4 and 5, respectively). Delightfully, we could quantify by ^1^H NMR the expected product **3aa** in 94% yield after 19 h of irradiation when Fukuzumi’s photocatalyst ([Mes-Acr-Me][BF_4_])^46^ was employed. After, we proceeded to evaluated different solvents (entries 7–11) with [Mes-Acr-Me][BF_4_] photocatalyst. When DMF was used as solvent, we could observe only 41% yield of **3aa**, after 24 h of irradiation (entry 7). To our delight, when the reaction was performed in dichloromethane (DCM), the product **3aa** was found in quantitative yield after only 9 h of irradiation (entry 8). However, the reaction did not proceed at all in toluene, probably due to the low solubility of both photocatalyst and 3,4-dihydroquinoxalin-2-one **1a** in this solvent (entry 9). Other chlorinated solvents such as 1,2-dichloroethane (DCE) and chloroform, were also tested obtaining high yields for product **3aa**, but the performance of DCM as solvent was slightly better. The variation of the equivalents of **1a** (entry 12) or **2a** (entry 13) did not improve the yield of the reaction. The use of Et_3_N-deactivated silica gel as stationary phase allowed us to purify product **3aa** without observing decomposition, and **3aa** was isolated in 99% yield (entry 8). Additionally, control experiments showed that the photocatalyst, visible-light irradiation, and an inert atmosphere are essential for the success of this transformation (entries 14–16). Moreover, product **3aa** was not observed when the reaction was performed under oxygen atmosphere or in the presence of 1.5 equiv of the radical scavenger TEMPO (entry 17).

After establishing the optimized reaction conditions to carry out the photocatalytic 1,6-addition reaction of 3,4-dihydroquinoxalin-2-one **1a** to *para*-quinone methide **2a**, we wanted to explore the generality of this transformation. First, the versatility of the cyclic amines was investigated. Different substituted 3,4-dihydroquinoxalin-2-ones with different electronic and steric properties were tested in the reaction with *para*-quinone methide **2a** and the corresponding addition products **3aa**–**3ia** could be obtained with good to excellent yields (Scheme 2). Initially, we studied the effect of different substituents at the aminic nitrogen (R^1^) of 3,4-dihydroquinoxalin-2-one **1**. The presence of a more electron-rich benzylic substituent such as the *para*-methoxybenzyl group resulted in the corresponding product **3ba** with an excellent 99% yield, comparable with that of compound **3aa**. Similarly, the presence of a methyl or CH_2_CO_2_Me group at this nitrogen of the dihydroquinoxalin-2-one moiety was allowed, and the corresponding products **3ca** and **3da**, were obtained in 91 and 81% yield, respectively. In any case, we did not observe the product functionalized at exocyclic CH_2_ of amines **1**. When we tested the reaction with *N*-4 unprotected quinoxalin-2-one derivative **1e**, we isolated *N*-alkylated product **4ea** in 44% yield after 15 h. This product corresponds to the 1,6-aza-conjugate addition reaction to *para*-quinone methide **2a**. Actually, we confirmed that this reaction should be mediated by visible light, since if it is performed in the dark, product **4aa** was only isolated in 11% yield after 3 days. To our delight, 3,4-dihydroquinoxalin-2-one bearing an electron-donating (Me) or electron-withdrawing (F) group at different positions of the parent aromatic ring furnished the corresponding phenols **3fa** and **3ga** in good to excellent yields (58 and 95%, respectively). Moreover, 1,4-disubstituted-3,4-dihydroquinoxalin-2-ones could be used under the optimized reaction conditions giving the corresponding products **3ha** and **3ia** with high yield, even with the presence of a strong electron-withdrawing group (CF_3_) at the C-7 position of the aromatic ring of the 3,4-dihydroquinoxalin-2-one.

**Scheme 2:**
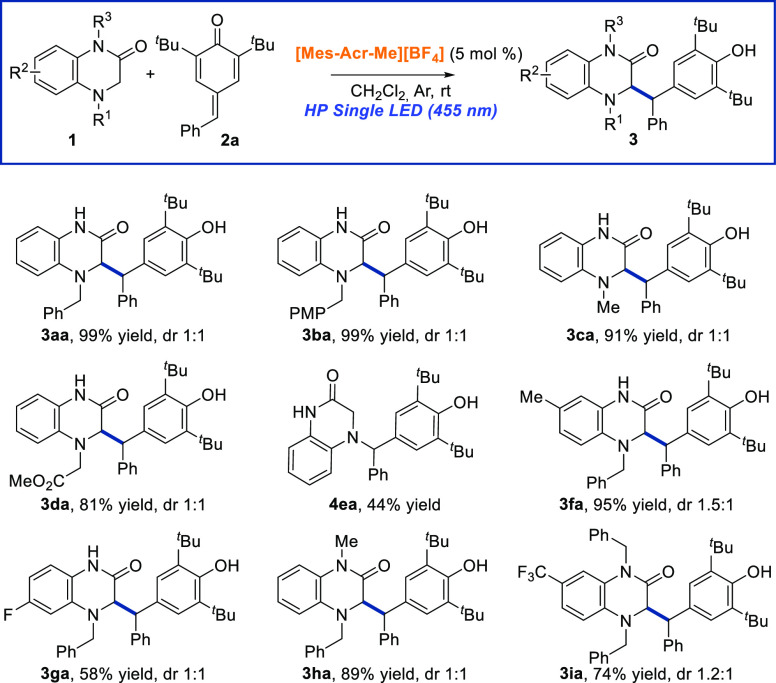
Scope of the 1,6-Radical Addition Reaction Regarding the Dihydroquinoxalin-2-one Derivatives 1 Reaction conditions: **1** (0.15 mmol), **2a** (0.1 mmol), [Mes-Acr-Me][BF_4_] (5 mol %), DCM (1 mL), under argon atmosphere and under HP Single LED (455 nm) irradiation for 6–16 h. Diastereomeric ratio was determined by ^1^H NMR of the crude reaction mixture. Yield determined after purification by column chromatography using Et_3_N-deactivated silica gel.

Subsequently, the scope and limitation of *para*-quinone methides **2** were explored (Scheme 3). Initially, we envisioned that it would be of interest to carry out this photochemical reaction with all the regioisomeric MeO-substituted *para*-quinone methides at the aromatic ring (**2b**–**2d**). Independently of the position of methoxy group, we could isolate the corresponding products with excellent yields (86–97%). Next, we evaluated the incorporation of electron-withdrawing groups such as halogens (Cl or Br), NO_2_, or CN on the benzene ring of the *para*-quinone methide **2**, and we observed that the presence of these groups had no remarkable impact on the reaction and the corresponding products (**3ae** – **3ah**) were obtained very high yields. Moreover, the reaction tolerates *para*-quinone methides bearing different hydroxyl groups protected with *tert*-butyldimethylsilyl or acetyl groups. Besides, a *para*-quinone methide with an alkyl group (Me) at the electrophilic position was tolerated under the optimized reaction conditions providing the expected product (**3ak**) in quantitative yield. Finally, we demonstrated the utility of our protocol for the late-stage functionalization of structurally diverse pharmaceutically relevant substances using a sophisticated *para*-quinone methide **2l** resulting from the incorporation of the indomethacin core, a nonsteroidal anti-inflammatory drug. This derivative was subjected to our organophotoredox 1,6-radical addition protocol furnishing the desired dihydroquinoxalin-2-one derivative **3al** bearing the indomethacin scaffold in 79% yield.

**Scheme 3:**
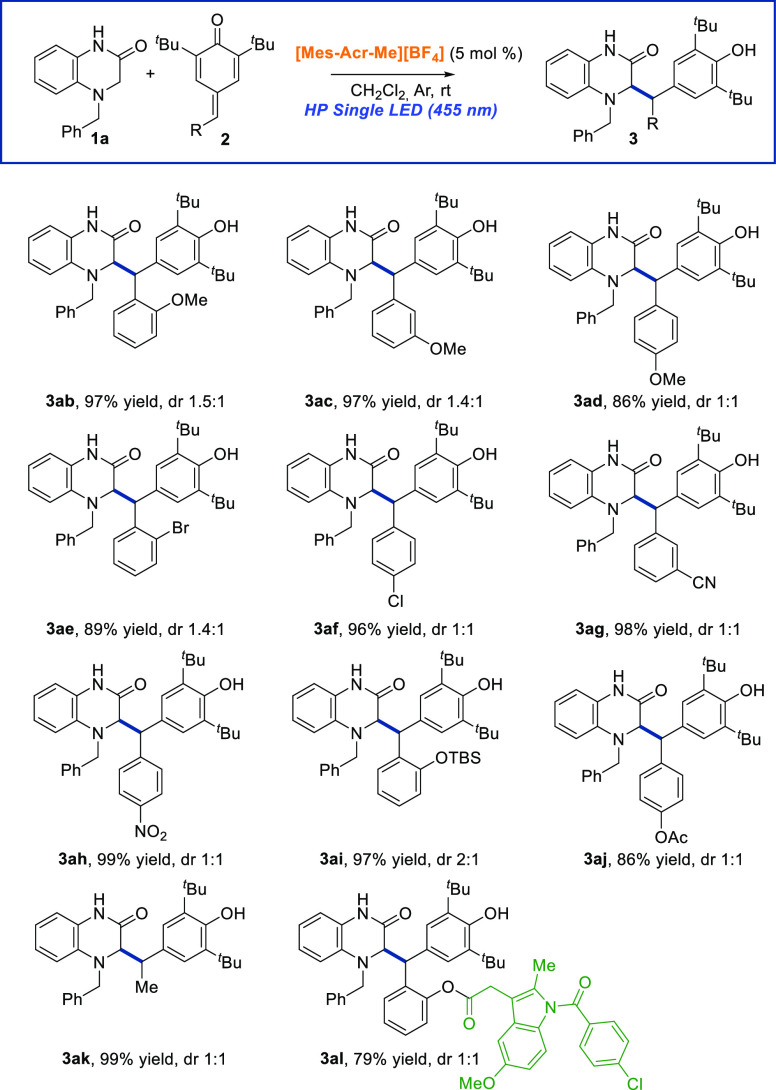
Scope of the 1,6-Radical Addition Reaction Regarding the *para*-Quinone Methides Derivatives 2 Reaction conditions: **1a** (0.15 mmol), **2** (0.1 mmol), [Mes-Acr-Me][BF_4_] (5 mol %), DCM (1 mL), under argon atmosphere and under HP Single LED (455 nm) irradiation for 6–16 h. Diastereomeric ratio was determined by ^1^H NMR of the crude reaction mixture. Yield determined after purification by column chromatography using Et_3_N-deactivated silica gel.

To gain insight into the mechanism of the reaction, we first examined the reduction potential values of each component in the reaction mixture. According to the literature, [Mes-Acr-Me]*^+^ has a reduction potential of +1.88 V (vs SCE) from its T_1_ excited state and a reduction potential of +2.18 V (vs SCE) from its S_1_ excited state.^47,48^ Curiously, since [Mes-Acr-Me]^+^ does not exhibit reductive abilities, it can only participate in reductive quenching cycles. Regarding both substrates, the reduction potential of 3,4-dihydroquinoxalin-2-one **1a** was already determined by us,^35^ and it was +0.80 V (vs SCE). The reduction potential of *para*-quinone methide **2a** was determined by Tang, Cai, and co-workers, and it was found to be −1.18 V (vs SCE).^27^ Hence, according to these data, the most probable pathway involves a single electron transfer between the excited state of [Mes-Acr-Me]^+^ and **1a**. To prove this thermodynamic assumption, we decided to perform steady-state luminescence quenching experiments. The study of the luminescence quenching of [Mes-Acr-Me]^+^ by **2a** was already reported in the bibliography by Ao, Liu, and co-workers.^23^ They found that *para*-quinone methide **2a** was not able to quench the excited state of [Mes-Acr-Me]^+^. Therefore, we only tested the ability of 3,4-dihydroquinoxalin-2-one **1a** to quench the excited photocatalyst. Luminescence quenching experiments are summarized in Figure 1A.^49^ According to these studies, 3,4-dihydroquinoxalin-2-one **1a** could quench the photoexcited [Mes-Acr-Me]^+^ effectively, and therefore, we can establish a Stern–Volmer constant (*K*_SV_) of 127 M^–1^ (Figure 1B). Additionally, to confirm the participation of a closed photoredox catalytic cycle and exclude a radical chain process, we determined the quantum yield of the process.^49^ We found out that the quantum yield of our photochemical reaction is as low as Φ = 0.040 ± 0.004, showing that the participation of a chain mechanism is unlikely.

**Figure 1:**
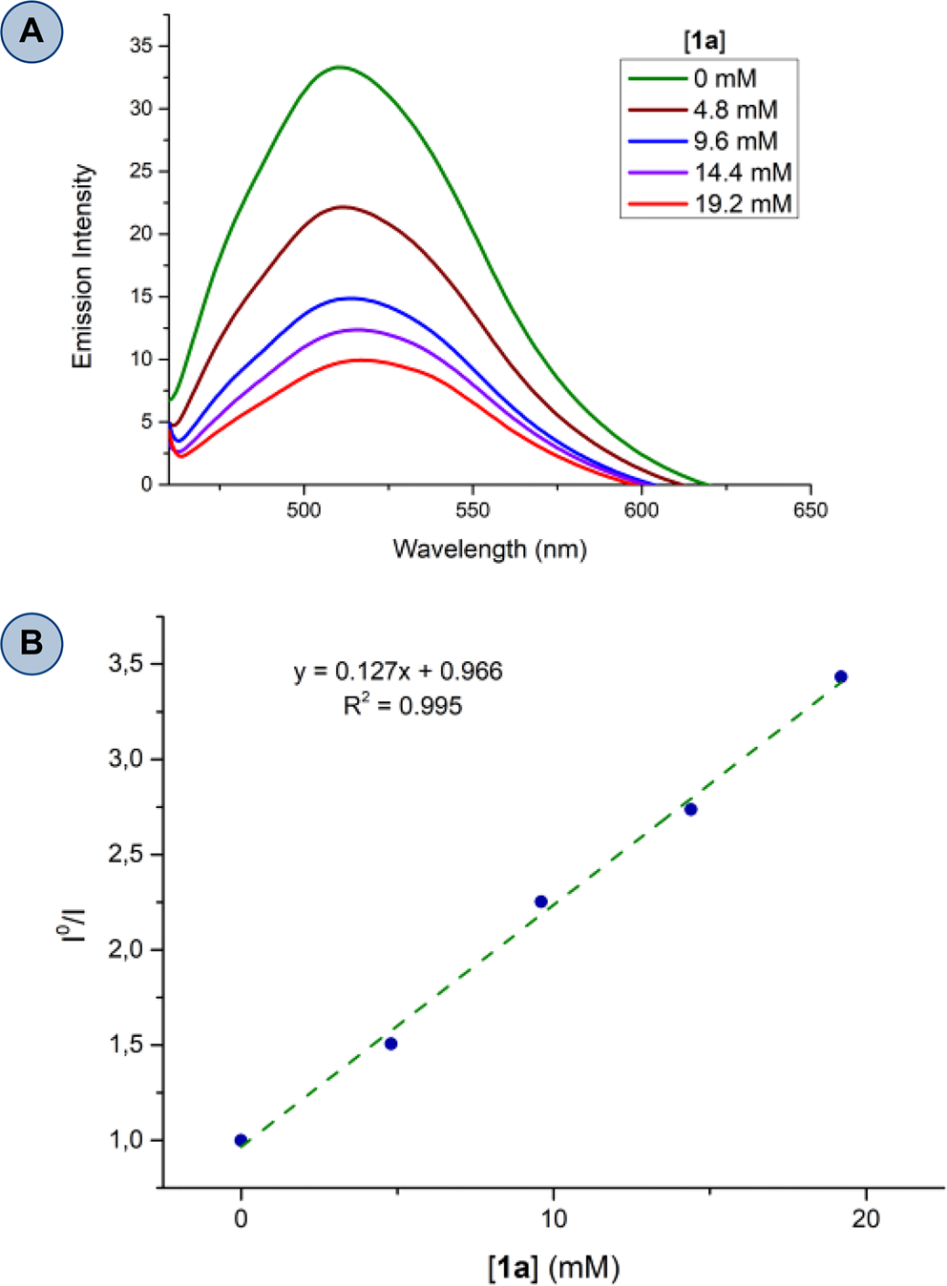
(A) Emission spectra of different DCM solutions containing 0.02 mM of [Mes-Acr-Me][BF_4_] and varying amounts of 3,4-dihydroquinoxalin-2-one 1a. (B) Stern–Volmer plot of I^0^/I vs [1a]. Determination of *K*_SV_ through linear regression.

With all this information, we were able to postulate a plausible reaction mechanism for our photochemical protocol (Scheme 4). Dihydroquinoxalin-2-one **1a**, can be engaged in a single electron transfer (SET) with the excited state form of [Mes-Acr-Me]*^+^ which results after the irradiation with 455 nm light. The SET results in the formation of the corresponding radical cation **I**, which can suffer the loss of a proton at its α position to generate the nucleophilic α-amino radical **II**. This carbon centered radical **II** is nucleophilic enough to react with the electrophilic exocyclic carbon of *para*-quinone methide **2a** in a 1,6-fashion. The product of this radical 1,6-addition may be O-centered radical **III**. Taking into account the oxidative potential of the radical intermediate [Mes-Acr-Me]^●^ (E_1/2_ = −0.57 V),^50^ the phenoxyl radical **III** could readily oxidize it, *via* SET, into [Mes-Acr-Me]^+^,^51,52^ and yield alkoxide **IV**. Finally, a proton transfer over **IV** affords the desired product **3aa**.

**Scheme 4:**
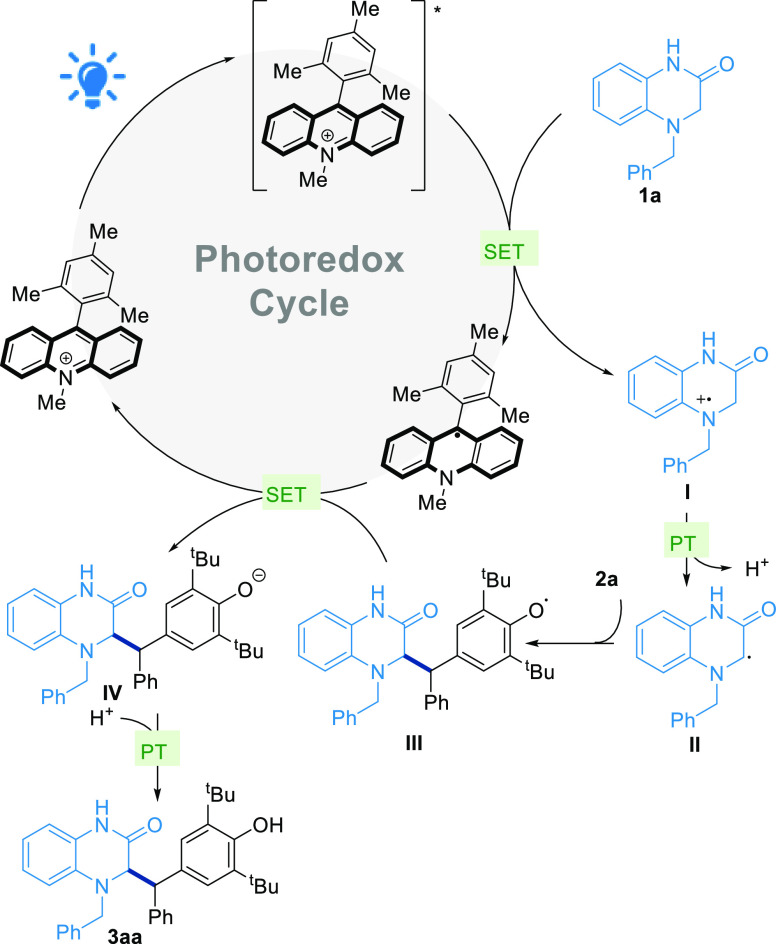
Mechanistic Hypothesis for the Photochemical 1,6-Radical Addition for the Synthesis of 3

In summary, we have developed a 1,6- radical addition of 3,4-dihydroquinoxalin-2-one derivatives with several *para*-quinone methides using visible-light organophotoredox catalysis. Our methodology provides a rapid and efficient access to functionalized phenols bearing a dihydroquinoxalin-2-one moiety under mild reaction conditions and simple operational protocol using the irradiation of HP single LED of 455 nm. Also a series of experiments have been carried out in order to gain insights into the reaction mechanism.

## Data Availability

The data underlying this study are available in the published article and its Supporting Information.

## Supporting Information Available

Complete experimental procedures, photochemical setup, quantum yield determination, characterization of new products and ^1^H and ^13^C NMR spectra for all compounds (PDF)

## Abbreviations

HP: high power
LED: light-emitting diode
4-CzIPN: 2,4,5,6-tetrakis(9*H*-carbazol-9-yl) isophthalonitrile
DMF: *N*,*N*-dimethylformamide
DCM: dichloromethane
DCE: 1,2-dichloroethane
TEMPO: 2,2,6,6-tetramethylpiperidine 1-oxyl
