# Supplementary material for: Organophotoredox 1,6-Addition of 3,4-Dihydroquinoxalin-2-ones to para-Quinone Methides Using Visible Light
Source: ACS Org Inorg Au. 2023 Jan 20;3(3):130–5. doi: 10.1021/acsorginorgau.2c00064 (PMC10251499; doi:10.1021/acsorginorgau.2c00064)

Organophotoredox 1,6-Addition of 3,4-Dihydroquinoxalin-2-ones to *para*-Quinone Methides using Visible Light

Jaume Rostoll-Berenguer,[a] Víctor García-García,[a] Gonzalo Blay,[a] José R. Pedro,*[a] Carlos Vila*[a].

[a]Departament de Química Orgànica, Facultat de Química, Universitat de València, Dr. Moliner 50, 46100 Burjassot, València (Spain). Email: jose.r.pedro@uv.es; carlos.vila@uv.es

General Experimental Methods

Reaction Flasks, Reagents and Substrates

- Photochemical reactions were carried out in 10 mL culture tubes under argon unless otherwise indicated.
- Commercial reagents were used as purchased.
- DCM was degassed by Ar bubbling and stored over 3Å MS for 48 h at least. Prior to use, DCM was bubbled with Ar for 10 min.
- All photocatalysts were commercially available.
- 4-Substituted-3,4-dihydroquinoxalin-2-ones **1a**-**1i** were prepared following reported procedures.[1-5]
- *p*-Quinone methides **2a**-**2j** were prepared following reported procedures.[6]

Chromatographic Methods

- Reactions were monitored by TLC analysis using Merck Silica Gel 60 F-254 thin layer plates and these are visualized using both a UV lamp (254 nm) and then a CAM solution (10 g of $Ce(SO_4)_2$, 25 g of phosphomolybdic acid and 80 mL of concentrated H_2SO_4, then diluted until 1 L with deionized water).
- Flash column chromatography was performed on Merck Silica Gel 60, 0.040-0.063 mm as stationary phase.
- Mobile phases were prepared by mixing solvents (hexanes, EtOAc, DCM), which have technical quality or higher.

Nuclear Magnetic Resonance (NMR)

- NMR spectra were run in a Bruker Avance 300 DPX at 300 MHz for ^{1}H, 282 MHz for ^{19}F and 75 MHz for ^{13}C using residual nondeuterated solvent as internal standard ($CHCl_3$: δ 7.26 and δ 77.00 ppm respectively, MeOH: δ 3.34 ppm and δ 49.87 ppm respectively, acetone-d_6): δ 2.05 ppm and δ 29.84 ppm respectively, DMSO-d_6: δ 2.5 and δ 39.52 ppm respectively).
- Some spectra were run using a Bruker Acance 500 DPX at 500 MHz for ^{1}H and 126 MHz for ^{13}C.
- Chemical shifts (δ) are given in ppm and coupling constants (*J*) in Hz.
- The carbon multiplicity was established by DEPT experiments.

High Resolution Mass Spectrometry (HRMS)

- High resolution mass spectra (HRMS-ESI) were recorded on an AB SCIEX Triple TOFTM spectrometer equipped with an electrospray source with a capillary voltage of 4.5 kV (ESI).

Fourier-transform Infrared spectroscopy (FTIR)

- Fourier-transform Infrared spectroscopy (FTIR) were recorded on an Agilent Carry 630 FTIR spectrometer.

Synthetic Procedures and Characterization

Specific Procedure 1 (SP-1) for the synthesis of indomethacin-derived *para*-quinone methide 2l

In a 50 mL round bottomed flask was placed commercially available indomethacin (394 mg, 1.1 mmol, 1 equiv.). It was dissolved in DCM (10 mL) and the resulting solution was cooled down to 0 °C. Then, a catalytic amount of DMF (2 drops) was added. Thereafter, oxalyl chloride (121 μL, 1.43 mmol, 1.1 equiv.) was added dropwise at that temperature and then the resulting solution was stirred for 3 hours at room temperature. After this time, the reaction mixture was evaporated to dryness under reduced pressure. Indomethacin acid chloride (398 mg, 1.1 mmol, 99% yield) was obtained as a brown solid, which was directly used in the next step without further purification.

In a 50 mL round bottomed flask was placed 2,6-di-*tert*-butyl-4-(2-hydroxybenzylidene)cyclohexa-2,5-dien-1-one (310 mg, 1 mmol, 1 equiv.) and was dissolved in DCM (10 mL). Then, Et_3N (0.17 mL, 1.2 mmol, 1.2 equiv.) was added. The resulting reaction mix- ture was cooled down to 0 °C, and a solution of indomethacin acid chloride (398 mg, 1.1 mmol, 1.1 equiv.) in DCM (5 mL) was added dropwise. The reaction was further stirred for 2 hours at 0 °C. After this time, the solution was partitioned with H_2O and separated. The aqueous phase was further extracted with DCM (x2) and the combined organic layers were dried over $MgSO_4$, filtered and evaporated under reduced pressure. The oily residue was purified by column chromatography using hexane:DCM 1:1 as eluent to afford the *p*-quinone methide **2l** (453 mg, 0.70 mmol, 70% yield) as a bright yellow solid (m.p. 93-95ºC).

2-((3,5-Di-*tert*-butyl-4-oxocyclohexa-2,5-dien-1-ylidene)methyl)phenyl 2-(1-(4-chlorobenzoyl)-5-methoxy-2-methyl-1*H*-indol-3-yl)acetate (2l)

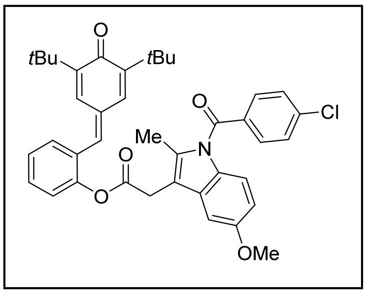

^{1}H-NMR (300 MHz, $CDCl_3$) δ 7.64 (d, J=8.7 Hz, 2H), 7.51–7.27 (m, 6H), 7.22–7.13 (m, 1H), 7.03 (d, J=2.4 Hz, 1H), 6.96–6.90 (m, 1H), 6.86 (s, 1H), 6.77–6.64 (m, 2H), 3.92 (s, 2H), 3.77 (s, 3H), 2.43 (s, 3H), 1.27 (s, 9H), 1.26 (s, 9H); **^{13}C{^{1}H}-NMR (75 MHz, $CDCl_3$)** δ 186.5 (C) 168.8 (C), 168.2 (C), 156.1 (C), 149.5 (C), 149.3 (C), 148.1 (C), 139.5 (C), 136.2 (C), 135.8 (CH), 134.2 (CH), 133.6 (C), 133.2 (C), 132.0 (CH), 131.2 (CH), 130.8 (C), 130.3 (C), 130.3 (CH), 129.1

(CH), 128.7 (C), 127.8 (CH), 126.1 (CH), 122.7 (CH), 115.1 (CH), 111.9 (CH), 111.6 (C), 101.1 (CH), 55.6 (CH_3), 35.4 (CH_2), 34.9 (C), 30.5 (C), 29.5 (CH_3), 29.4 (CH_3), 13.4 (CH_3); **HRMS (ESI/Q-TOF)** *m/z* $[M + H]^+$ calcd for $C_{40}H_{41}ClNO_5^+$ 650.2668, found 650.2671. **FTIR (neat)** cm^{-1} : 2952 (w), 1755 (m), 1684 (s), 1613 (s), 1315 (s), 1215 (s), 1118 (s), 753 (s).

General Procedure 1 (GP-1) for the Photocatalytic Reaction between 3,4-dihydro-quinoxalin-2-ones 1 and ara*p*-quinone methides 2

In an ovendried 10 mL culture tube, the corresponding 3,4-dihydroquinoxalin-2-one (**1**, 0.15 mmol, 0.15 equiv.), the corresponding *p*-quinone methide (**2**, 0.1 mmol, 1 equiv.) and [Mes-Acr-Me][BF_4] (2 mg, 5 mol %) were placed. Then, anhydrous and degassed DCM (1 mL) was added, and the solution was bubbled with Ar for 10 minutes at 0 ° to prevent evaporation. The reaction mixture was stirred under the irradiation of a HP Single LED (455 nm) while being cooled with a fan to keep the temperature at approximately 25 ^{o}C. Once the reaction was finished (TLC), the mixture was analyzed by ^{1}H-NMR to determine the diastereomeric ratio. Thereafter, it was purified by column chromatography using hexane:EtOAc mixtures to afford the corresponding compound **3**.

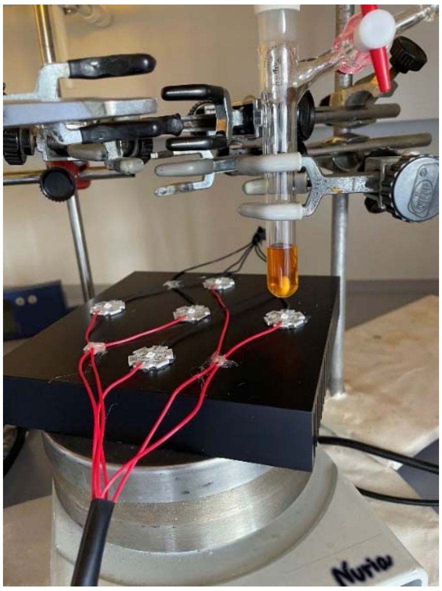

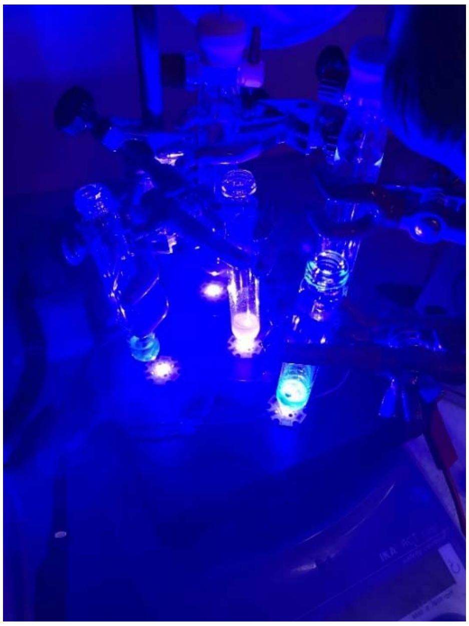

Specific Procedure for the photochemical reaction between 1a and 2a in the presence of TEMPO:

In an ovendried 10 mL culture tube, 3,4-dihydroquinoxalin-2-one **1a** (0.15 mmol, 1.5 equiv.), *p*-quinone methide **2a** (0.1 mmol, 1 equiv.), TEMPO (0.15 mmol, 1.5 equiv.) and

[Mes-Acr-Me][BF_4] (2 mg, 5 mol %) were placed. Then, anhydrous and degassed DCM (1 mL) was added, and the solution was bubbled with Ar for 10 minutes at 0 ºC to prevent evaporation. The reaction mixture was stirred under the irradiation of a HP Single LED (455 nm) while being cooled with a fan to keep the temperature at approximately 25 ºC. After 16 hours, the reaction mixture was removed from the light source, filtered over a small pad of silica (eluting with EtOAc), concentrated and analyzed by ^{1}H-NMR. The crude reaction mixture shown no conversion towards the desired product **3aa**.

Specific Procedure for the 1 mmol-scale reaction between 1a and 2a using sunlight:

In a 50 mL Schlenk flask, 3,4-dihydroquinoxalin-2-one **1a** (357 mg, 1.5 mmol, 1.5 equiv.), *p*-quinone methide **2a** (294 mg, 1 mmol, 1 equiv.) and [Mes-Acr-Me][BF_4] (20 mg, 5 mol %) were placed. Then, anhydrous and degassed DCM (10 mL) was added, and the solution was bubbled with Ar for 10 minutes at 0 ºC to prevent evaporation. The reaction was placed at the upper part of the building in sunny hours (Burjassot, 9th June, 2022 from 10:30 am to 6:30 pm) and stirred under sunlight irradiation. Once the reaction was finished (TLC), the mixture was analyzed by ^{1}H-NMR to determine the diastereomeric ratio. Thereafter, it was purified by column chromatography using hexane:EtOAc mixtures to afford the corresponding compound **3aa** (229 mg, 0.43 mmol, 43% yield).

Deactivation of Silica Gel with Et_3N:

In a 1L-round bottomed flask was placed silica gel (approximately filling half of the volume) and it was suspended with DCM. Then Et_3N (10 mL) were added in one portion and the resulting suspension was stirred vigorously. Thereafter, volatiles were removed under reduced pressure to obtain a powdery material, which was further dried under vacuum. The obtained deactivated Silica Gel was employed as stationary phase for the purification of compounds **3**.

Photon Flux of HP Single LED (455 nm)

In a previous work,[7] we determined the photon flux of HP Single LED (455 nm) by ferrioxalate actinometry, and it was found to be $4.18 \cdot 10^{-7}$ einsteins/s.

4-Benzyl-3-((3,5-di-*tert*-butyl-4-hydroxyphenyl)(phenyl)methyl)-3,4-dihydroquinoxalin-2(1*H*)-one (3aa)

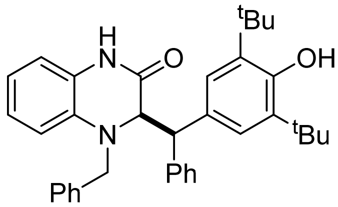

Using 4-benzyl-3,4-dihydroquinoxalin-2-one (**1a**, 38.4 mg, 0.15 mmol, 1.5 equiv.) and 4-benzylidene-2,6-di-*tert*-butylcyclohexa-2,5-dien-1-one (**2a**, 29.4 mg, 0.1 mmol, 1 equiv.), according to GP-1, compound **3aa** (52.5 mg, 0.099 mmol, 99% yield) was obtained as a mixture of diastereomers (1:1 dr) that were separated by column chromatography using hexane:EtOAc mixtures (from 9:1 to 8:2).

Characterization of **3aa'**: yellow oil; **^{1}H-NMR (300 MHz, CDCl$_3$)** δ 8.33 (s, 1H), 7.31–7.27 (m, 5H), 7.25–7.16 (m, 3H), 7.05 (dd, *J*=7.5, 2.0 Hz, 2H), 7.00 (s, 2H), 6.93 (td, *J*=7.7, 1.5 Hz, 1H), 6.76 (td, *J*=7.6, 1.3 Hz, 1H), 6.61 (td, *J*=7.8, 1.4 Hz, 2H), 5.00 (s, 1H), 4.59 (dd, *J*=8.1, 0.8 Hz, 1H), 4.23 (d, *J*=15.4 Hz, 1H), 4.08 (d, *J*=8.1 Hz, 1H), 3.76 (d, *J*=15.5 Hz, 1H), 1.33 (s, 18H). **^{13}C{^{1}H}-NMR (75 MHz, CDCl$_3$)** δ 165.5 (C), 152.5 (C), 142.4 (C), 137.0 (C), 135.1 (C), 133.6 (C), 129.8 (C), 128.8 (C), 128.8 (CH), 128.52 (CH), 128.48 (CH), 127.5 (CH), 127.3 (CH), 126.7 (CH), 125.7 (CH), 123.9 (CH), 119.2 (CH), 115.2 (CH), 114.5 (CH), 66.8 (CH), 54.0 (CH$_2$), 53.0 (CH), 34.3 (C), 30.3 (CH$_3$). **HRMS (ESI/Q-TOF)** *m/z* [M + H]$^+$ calcd for $C_{36}H_{41}N_2O_2^+$ 533.3163, found 533.3179. **FTIR (neat)** cm^{-1} : 3625 (w), 2955 (w), 1677 (s), 1431 (m), 742 (s), 697 (s).

Characterization of **3aa"**: yellow oil; **^{1}H-NMR (300 MHz, CDCl$_3$)** δ 8.40 (s, 1H), 7.40–7.32 (m, 2H), 7.29–7.11 (m, 6H), 7.03–6.93 (m, 5H), 6.83 (td, *J*=7.6, 1.2 Hz, 1H), 6.70 (dd, *J*=7.7, 1.4 Hz, 1H), 6.53 (dd, *J*=8.1, 1.2 Hz, 1H), 5.19 (s, 1H), 4.51 (dd, *J*=10.5, 1.1 Hz, 1H), 4.06–3.93 (m, 2H), 3.48 (d, *J*=15.3 Hz, 1H), 1.43 (s, 18H). **^{13}C{^{1}H}-NMR (75 MHz, CDCl$_3$)** δ 165.7 (C), 153.1 (C), 140.0 (C), 137.1 (C), 136.1 (C), 133.5 (C), 131.6 (C), 128.8 (CH), 128.6 (CH), 128.2 (CH), 127.44 (CH), 127.38 (CH), 126.91 (C), 126.89 (CH), 125.5 (CH), 123.9 (CH), 119.2 (CH), 115.6 (CH), 114.9 (CH), 66.3 (CH), 54.0 (CH$_2$), 52.1 (CH), 34.4 (C), 30.4 (CH$_3$); **HRMS (ESI/Q-TOF)** *m/z* [M + H]$^+$ calcd for $C_{36}H_{41}N_2O_2^+$ 533.3163, found 533.3168. **FTIR (neat)** cm^{-1} : 3630 (w), 2952 (w), 1679 (s), 1429 (m), 741 (s), 697 (s).

3-((3,5-Di-*tert*-butyl-4-hydroxyphenyl)(phenyl)methyl)-4-(4-methoxybenzyl)-3,4-dihydroquinoxalin-2(1*H*)-one (3ba)

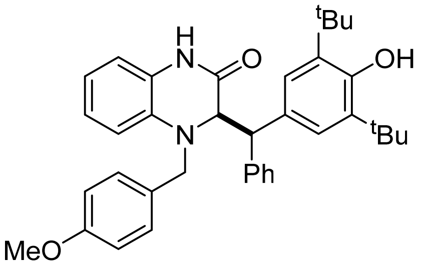

Using 4-(4-methoxybenzyl)-3,4-dihydroquinoxalin-2(1*H*)-one (**1b**, 40.2 mg, 0.15 mmol, 1.5 equiv.) and 4-benzylidene-2,6-di-*tert*-butylcyclohexa-2,5- dien-1-one (**2a**, 29.4 mg, 0.1 mmol, 1 equiv.), according to GP-1, compound **3ba** (55.7 mg, 0.099 mmol, 99% yield) was obtained as a mixture of diastereomers (1:1 dr) that were separated by column chromatography using hexane:EtOAc mixtures (from 9:1 to 8:2).

Characterization of **3ba'**: yellow oil; **^{1}H-NMR (300 MHz, CDCl$_3$)** δ 8.08 (sa, 1H), 7.29–7.21 (m, 5H), 6.99–6.94 (m, 4H), 6.92 (dd, J = 8.0, 1.4 Hz, 1H), 6.81–6.71 (m, 3H), 6.61 (ddd, J=7.7, 6.3, 1.3 Hz, 2H), 4.99 (s, 1H), 4.57 (d, J=8.0 Hz, 1H), 4.19 (d, J=15.0 Hz, 1H), 4.06 (d, J=8.0 Hz, 1H), 3.75 (s, 3H), 3.69 (d, J=15.0 Hz, 1H), 1.33 (s, 18H). **^{13}C{^{1}H}-NMR (75 MHz, CDCl$_3$)** δ 165.5 (C), 158.8 (C), 152.5 (C), 142.4 (C), 135.1 (C), 133.8 (C), 129.8 (C), 128.83 (CH), 128.75 (C), 128.4 (CH), 126.9 (C), 126.6 (CH), 125.7 (CH), 123.8 (CH), 119.1 (CH), 115.1 (CH), 114.6 (CH), 113.9 (CH), 66.2 (CH), 55.2 (CH), 53.4 (CH$_2$), 52.8 (CH$_3$), 34.2 (C), 30.3 (CH$_3$) **HRMS (ESI/Q-TOF)** *m/z* [M + H]$^+$ calcd for $C_{37}H_{43}N_2O_3^+$ 563.3268, found 563.3252. **FTIR (neat)** cm^{-1} : 3630 (w), 2952 (w), 1669 (s), 1610 (s), 1248 (s), 745 (s), 697 (s).

Characterization of **3ba"**: yellow oil; **^{1}H-NMR (300 MHz, CDCl$_3$)** δ 8.39 (s, 1H), 7.34 (d, J=7.0 Hz, 1H), 7.28–7.20 (m, 3H), 7.20–7.07 (m, 1H), 7.04–6.95 (m, 3H), 6.90 (d, J=8.7 Hz, 2H), 6.83 (td, J=7.6, 1.2 Hz, 1H), 6.76 (d, J = 8.7 Hz, 2H), 6.68 (dd, J=7.7, 1.5 Hz, 1H),6.56 (d, J=7.6 Hz, 1H), 5.18 (s, 1H), 4.48 (dd, J = 10.5, 1.0 Hz, 1H), 4.0–3.85 (m, 2H), 3.76 (s, 3H), 3.38 (d, J=14.8 Hz, 1H), 1.43 (s, 18H). **^{13}C{^{1}H}-NMR (75 MHz, CDCl$_3$)** δ 165.6 (C), 158.9 (C), 153.0 (C), 140.0 (C), 136.0 (C), 133.7 (C), 131.6 (C), 128.9 (C), 128.75 (CH) 128.73 (CH), 128.2 (CH), 126.9 (C), 126.8 (CH), 125.4 (CH), 123.8 (CH), 119.1 (CH), 115.5 (CH), 114.9 (CH), 113.9 (CH), 65.5 (CH), 55.2 (CH), 53.3 (CH$_2$), 52.0 (CH$_3$), 34.4 (C), 30.3 (CH$_3$); **HRMS (ESI/Q-TOF)** *m/z* [M + H]$^+$ calcd for $C_{37}H_{43}N_2O_3^+$ 563.3268, found 563.3271. **FTIR (neat)** cm^{-1} : 3619 (w), 2955 (w), 1677 (s), 1509 (s), 1244 (s), 745 (s), 695 (s).

3-((3,5-Di-tert-butyl-4-hydroxyphenyl)(phenyl)methyl)-4-methyl-3,4-dihydroquinoxalin-2(1*H*)-one (3ca)

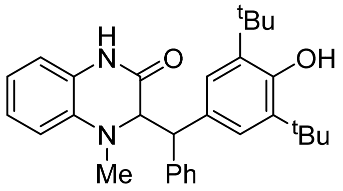

Using 4-methyl-3,4-dihydroquinoxalin-2(1*H*)-one (**1c**, 24.3 mg, 0.15 mmol, 1.5 equiv.) and 4-benzylidene-2,6-di-*tert*-butylcyclohexa-2,5-dien-1-one (**2a**, 29.4 mg, 0.1 mmol, 1 equiv.), according to GP- 1, compound **3ca** (41.5 mg, 0.091 mmol, 91% yield) was obtained as a mixture of diastereomers (1:1 dr) that can be separated by column chromatography using hexane:EtOAc mixtures (from 9:1 to 8:2).

Characterization of **3ca'**: yellow oil; **^{1}H NMR (500 MHz, CDCl$_3$)** δ 8.44 (s, 1H), 7.36 (d, *J*=7.5 Hz, 2H), 7.32–7.24 (m, 2H), 7.20 (t, *J*=7.3 Hz, 1H), 7.04–6.93 (m, 3H), 6.72 (t, *J*=7.4 Hz, 1H), 6.53 (t, *J*=7.6 Hz, 2H), 4.98 (s, 1H), 4.59 (d, *J*=7.2 Hz, 1H), 4.07 (d, *J*=7.2 Hz, 1H), 2.60 (s, 3H), 1.32 (s, 18H); **^{13}C{^{1}H} NMR (126 MHz, CDCl$_3$)** δ 165.2 (C), 152.4 (C), 142.6 (C), 135.1 (C), 134.5 (C), 129.9 (C), 128.7 (CH), 128.4 (CH), 126.6 (CH), 126.1 (C), 125.6 (CH), 123.9 (CH), 118.3 (CH), 114.7 (CH), 112.4 (CH), 68.8 (CH), 52.7 (CH$_3$), 37.9 (CH), 34.2 (C), 30.2 (CH$_3$); **HRMS (ESI/Q-TOF)** *m/z* [M + H]$^+$ calcd for $C_{30}H_{37}N_2O_2^+$ 457.2850, found 457.2856. FTIR (neat) cm-1 : 3625 (w), 2955 (w), 1677 (s), 1431 (m), 742 (s), 697 (s). **FTIR (neat)** cm^{-1} : 3630 (w), 2952 (m), 2922 (m), 1674 (s), 1613 (s), 1360 (m), 697 (s).

Characterization of **3ca''**: yellow oil; **^{1}H NMR (500 MHz, CDCl$_3$)** δ 8.32 (s, 1H), 7.37 (d, *J*=7.4 Hz, 2H), 7.29–7.22 (m, 2H), 7.16 (t, *J*=7.3 Hz, 1H), 7.07–6.98 (m, 3H), 6.78 (t, *J*=7.6 Hz, 1H), 6.64 (d, *J*=7.6 Hz, 1H), 6.44 (d, *J*=7.9 Hz, 1H), 5.11 (s, 1H), 4.52 (d, *J*=9.7 Hz, 1H), 3.94 (d, *J*=9.8 Hz, 1H), 2.36 (s, 3H), 1.39 (s, 18H); **^{13}C{^{1}H} NMR (126 MHz, CDCl$_3$)** δ 165.2 (C), 152.9 (C), 140.2 (C), 136.1 (C), 134.3 (C), 131.6 (C), 128.6 (CH), 128.2 (CH), 126.7 (CH), 126.0 (C), 125.3 (CH), 124.0 (CH), 118.3 (CH), 115.1 (CH), 112.9 (CH), 68.9 (CH), 52.4 (CH3), 38.4 (CH), 34.3 (C), 30.3 (CH$_3$); **HRMS (ESI/Q-TOF)** *m/z* [M + H]$^+$ calcd for $C_{30}H_{37}N_2O_2^+$ 457.2850, found 457.2854. **FTIR (neat)** cm^{-1} : 3630 (w), 2959 (w), 2870 (w), 1677 (s), 1613 (s), 1360 (m), 745 (s), 697 (s).

Methyl 2-(2-((3,5-di-*tert*-butyl-4-hydroxyphenyl)(phenyl)methyl)-3-oxo-3,4-dihydroquinoxalin-1(2*H*)-yl)acetate (3da)

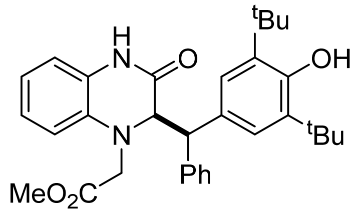

Using methyl 2-(3-oxo-3,4-dihydroquinoxalin-1(2*H*)-yl) acetate (**1d**, 33.0 mg, 0.15 mmol, 1.5 equiv.) and 4-benzylidene-2,6-di-*tert*-butylcyclohexa-2,5-dien-1- one (**2a**, 29.4 mg, 0.1 mmol, 1 equiv.), according to GP- 1, compound **3da** (41.7 mg, 0.081 mmol, 81% yield, yellow oil) was obtained as a mixture of diastereomers (1:1 dr) that cannot be separated by column chromatography using hexane:EtOAc mixtures (from 9:1 to 8:2).

^{1}H-NMR (300 MHz, CDCl$_3$) δ 8.97 (s, 1H), 8.86 (s, 1H), 7.39 (d, *J*=7.1 Hz, 2H), 7.31–7.13 (m, 8H), 7.10 (s, 2H), 7.00 (s, 2H), 6.99–6.91 (m, 2H), 6.85-6.75 (m, 2H), 6.67 (dd, *J*=7.7, 1.4 Hz, 1H), 6.59-6.55 (m, 2H), 6.48 (dd, *J*=8.1, 1.1 Hz, 1H), 5.14 (s, 1H), 5.04 (s, 1H), 4.55-4.51 (m, 2H), 4.10 (d, *J*=8.1 Hz, 1H), 3.96 (d, *J*=9.7 Hz, 1H), 3.84 (d, *J*=18.0 Hz, 1H), 3.69 (d, *J*=17.8 Hz, 1H), 3.59 (s, 6H), 3.42 (d, *J*=18.0 Hz, 1H), 3.17 (d, *J*=17.9 Hz, 1H), 1.38 (s, 18H), 1.36 (s, 18H). **^{13}C{^{1}H}-NMR (75 MHz, CDCl$_3$)** δ 170.5 (C), 170.2 (C), 165.5 (C), 165.3 (C), 152.9 (C), 152.5 (C), 141.7 (C), 139.9 (C), 136.0 (C), 135.2 (C), 132.6 (C), 132.4 (C), 131.0 (C), 129.7 (C), 128.73 (CH), 128.68 (CH), 128.4 (CH), 128.2 (CH), 127.19 (C), 127.12 (C), 126.8 (CH), 126.7 (CH), 125.6 (CH), 125.3 (CH), 123.8 (CH), 119.9 (CH), 115.9 (CH), 115.7 (CH), 114.6 (CH), 114.5 (CH), 68.3 (CH), 67.8 (CH), 53. (CH$_2$), 53.7 (CH), 53.0 (CH$_2$), 52.9 (CH), 52.0 (2xCH$_3$), 34.3 (C), 34.2 (C), 30.3 (CH$_3$); **HRMS (ESI/Q-TOF)** *m/z* [M + H]$^+$ calcd for $C_{32}H_{39}N_2O_4^+$ 515.2904, found 515.2909. **FTIR (neat)** cm^{-1} : 3630 (w), 2950 (m), 1760 (s), 1684 (s), 1610 (m), 747 (s), 695 (s).

4-((3,5-Di-*tert*-butyl-4-hydroxyphenyl)(phenyl)methyl)-3,4-dihydroquinoxalin-2(1*H*)-one (4ea)

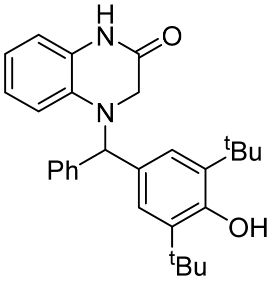

Using 3,4-dihydroquinoxalin-2(1*H*)-one (**1e**, 22.2 mg, 0.15 mmol, 1.5 equiv.) and 4-benzylidene-2,6-di-*tert*-butylcyclohexa-2,5-dien-1-one (**2a**, 29.4 mg, 0.1 mmol, 1 equiv.), according to GP-1, compound **4ea** (19.5 mg, 0.044 mmol, 44% yield, yellow oil) was obtained after column chromatography using hexane:EtOAc mixtures (from 9:1 to 8:2).

^{1}H-NMR (300 MHz, $CDCl_3$) δ 8.73 (s, 1H), 7.38–7.27 (m, 5H), 6.97 (d, *J*=0.6 Hz, 2H), 6.89 (ddd, *J*=8.2, 6.3, 2.6 Hz, 1H), 6.81–6.70 (m, 3H), 5.93 (s, 1H), 5.22 (s, 1H), 3.77 (d, *J*=2.5 Hz, 2H), 1.36 (s, 18H). **$^{13}C\{^1H\}$-NMR (75 MHz, $CDCl_3$)** δ 167.5 (C), 153.3 (C), 139.9 (C), 135.9 (C), 135.5 (C), 129.1 (C), 128.5 (CH), 128.3 (CH), 127.3 (CH), 126.5 (C), 125.9 (CH), 124.0 (CH), 118.8 (CH), 115.5 (CH), 114.0 (CH), 66.1 (CH), 48.7 (CH_2), 34.3 (C), 30.3 (CH_3); **HRMS (ESI/Q-TOF)** *m/z* $[M+H]^+$ calcd for $C_{29}H_{35}N_2O_2^+$ 443.2693, found 443.2697. **FTIR (neat)** cm^{-1} : 3630 (w), 2955 (m), 1680 (s), 741 (s), 697 (s).

4-Benzyl-3-((3,5-di-*tert*-butyl-4-hydroxyphenyl)(phenyl)methyl)-7-methyl-3,4-dihydroquinoxalin-2(1*H*)-one (3fa)

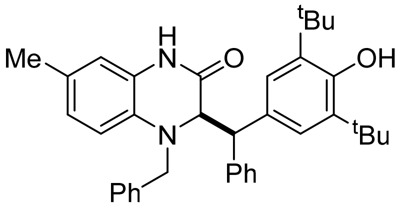

Using 4-benzyl-7-methyl-3,4-dihydroquinoxalin-2(1*H*)-one (**1f**, 37.4 mg, 0.15 mmol, 1.5 equiv.) and 4-benzylidene-2,6-di-*tert*-butylcyclohexa-2,5-dien-1-one (**2a**, 29.4 mg, 0.1 mmol, 1 equiv.), according to GP- 1, compound **3fa** (51.9 mg, 0.095 mmol, 95% yield) was obtained as a mixture of diastereomers (1.5:1 dr) that were separated by column chromatography using hexane:EtOAc mixtures (from 9:1 to 8:2).

Characterization of **3fa'**: yellow oil; **^{1}H-NMR (300 MHz, $CDCl_3$)** δ 8.38 (s, 1H), 7.29 (m, 4H), 7.25–7.16 (m, 4H), 7.04 (dd, *J*=7.4, 2.1 Hz, 2H), 7.00 (s, 2H), 6.73 (ddd, *J*=8.1, 1.9, 0.8 Hz, 1H), 6.50 (d, *J*=5.9 Hz, 1H), 6.48 (d, *J*=0.7 Hz, 1H), 4.99 (s, 1H), 4.56 (dd, *J*=8.1, 0.9 Hz, 1H), 4.19 (d, *J*=15.4 Hz, 1H), 4.09 (d, *J*=8.1 Hz, 1H), 3.77 (d, *J*=15.4 Hz, 1H), 2.25 (s, 3H), 1.34 (s, 18H). **$^{13}C\{^1H\}$-NMR (75 MHz, $CDCl_3$)** δ 166.0 (C), 152.5 (C), 142.6 (C), 137.2 (C), 135.1 (C), 131.3 (C), 130.4 (C), 129.90 (C), 128.85 (CH), 128.5 (CH), 127.6 (CH), 127.0 (CH), 126.9 (C), 126.6 (CH), 125.7 (CH), 124.4 (CH), 116.0 (CH), 114.8 (CH), 66.9 (CH), 54.2 (CH_2), 52.7 (CH), 34.3 (C), 30.3 (CH_3), 20.6 (CH_3). **HRMS (ESI/Q-TOF)** *m/z* $[M + H]^+$ calcd for $C_{37}H_{43}N_2O_2^+$ 547.3319, found 547.3336. **FTIR (neat)** cm^{-1} : 3593 (w), 2952 (m), 1669 (s), 1613 (s), 693 (s).

Characterization of **3fa"**: yellow oil; **^{1}H-NMR (300 MHz, $CDCl_3$)** δ 8.47 (s, 1H), 7.39–7.33 (m, 2H), 7.33–7.27 (m, 1H), 7.25–7.07 (m, 5H), 7.02 (s, 2H), 6.97 (dd, *J*=7.4, 2.1 Hz, 2H), 6.78 (ddd, *J*=8.1, 2.0, 0.8 Hz, 1H), 6.52 (s, 1H), 6.43 (d, *J*=8.1 Hz, 1H), 5.18 (s, 1H), 4.47 (dd, *J*=10.5, 1.1 Hz, 1H), 3.99-3.93 (m, 2H), 3.47 (d, *J*=15.3 Hz, 1H), 2.30 (s, 3H), 1.43 (s, 18H).**$^{13}C\{^1H\}$-NMR (75 MHz, $CDCl_3$)** δ 166.0 (C), 153.0 (C), 140.1 (C),

137.4 (C), 136.1 (C), 131.7 (C), 131.1 (C), 128.9 (C), 128.6 (CH), 128.5 (CH), 128.1 (CH), 127.4 (CH), 127.3 (CH), 126.9 (C), 126.8 (CH), 125.5 (CH), 124.3 (CH), 116.2 (CH), 115.1 (CH), 66.5 (CH), 54.2 (CH_2), 51.9 (CH), 34.4 (C), 30.3 (CH_3), 20.6 (CH_3); **HRMS (ESI/Q-TOF)** *m/z* $[M + H]^+$ calcd for $C_{37}H_{43}N_2O_2^+$ 547.3319, found 547.3328. **FTIR (neat)** cm^{-1} : 3593 (w), 2952 (m), 1684 (s), 1610 (s), 1360 (m), 693 (s).

4-Benzyl-3-((3,5-di-*tert*-butyl-4-hydroxyphenyl)(phenyl)methyl)-6-fluoro-3,4-dihydroquinoxalin-2(1*H*)-one (3ga)

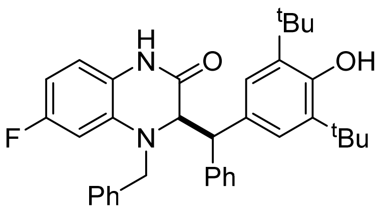

Using 4-benzyl-6-fluoro-3,4-dihydroquinoxalin-2(1*H*)-one (**1g**, 38.4 mg, 0.15 mmol, 1.5 equiv.) and 4-benzylidene-2,6-di-*tert*-butylcyclohexa-2,5-dien-1-one (**2a**, 29.4 mg, 0.1 mmol, 1 equiv.), according to GP-1, compound **3ga** (31.9 mg, 0.058 mmol, 58% yield) was obtained as a mixture of diastereomers (1:1 dr) that were separated by column chromatography using hexane:EtOAc mixtures (from 9:1 to 8:2).

<u>Characterization of **3ga'**:</u> colorless oil; **^{1}H-NMR (300 MHz, $CDCl_3$)** δ 8.44 (d, *J*=18.7 Hz, 1H), 7.32–7.18 (m, 8H), 7.06 (dd, *J*=7.4, 2.1 Hz, 2H), 7.00 (s, 2H), 6.48 (dd, *J*=8.5, 5.6 Hz, 1H), 6.41 (td, *J*=8.3, 2.5 Hz, 1H), 6.30 (dd, *J*=10.7, 2.5 Hz, 1H), 5.01 (s, 1H), 4.60 (d, *J*=7.8 Hz, 1H), 4.16 (d, *J* = 15.6 Hz, 1H), 4.09 (d, *J*=7.8 Hz, 1H), 3.78 (d, *J*=15.5 Hz, 1H), 1.33 (s, 18H). **^{19}F{^{1}H}-NMR (282 MHz, $CDCl_3$)** δ -118.71. **^{13}C{^{1}H}-NMR (75 MHz, $CDCl_3$)** δ 165.3 (C), 152.6 (C), 142.0 (C), 136.2 (C), 135.2 (C), 129.5 (C), 128.6 (CH), 127.6 (CH), 127.4 (CH), 126.8 (CH), 125.6 (CH), 122.8 (d, J_{C-F}=2.3 Hz, C), 115.5 (d, J_{C-F}=10.0 Hz, CH), 105.0 (d, J_{C-F}=23.3 Hz, CH), 101.6 (d, J_{C-F}=27.2 Hz, CH), 66.46 (CH), 53.8 (CH_2), 53.1 (CH), 34.2 (C), 30.2 (CH_3); **HRMS (ESI/Q-TOF)** *m/z* $[M + H]^+$ calcd for $C_{36}H_{40}FN_2O_2^+$ 551.3068, found 551.3072. **FTIR (neat)** cm^{-1} : 3619 (w), 2952 (m), 1684 (s), 1513 (s), 697 (s).

<u>Characterization of **3ga"**:</u> colorless oil; **^{1}H-NMR (300 MHz, $CDCl_3$)** δ 8.32 (s, 1H), 7.36–7.31 (m, 2H), 7.29–7.13 (m, 6H), 6.98 (d, *J*=7.9 Hz, 4H), 6.60 (dd, *J*=8.5, 5.5 Hz, 1H), 6.50 (td, *J*=8.4, 2.6 Hz, 1H), 6.22 (dd, *J*=10.6, 2.6 Hz, 1H), 5.20 (s, 1H), 4.50 (dd, *J*=10.4, 1.0 Hz, 1H), 3.95 (d, *J*=10.4 Hz, 1H), 3.89 (d, *J*=15.3 Hz, 1H), 3.47 (d, *J*=15.3 Hz, 1H), 1.42 (s, 18H). **^{19}F{^{1}H}-NMR (282 MHz, $CDCl_3$)** δ -118.61.**^{13}C{^{1}H}-NMR (75 MHz, $CDCl_3$)** δ 165.0 (C), 153.2 (C), 139.7 (C), 136.4 (C), 136.2 (C), 131.2 (C), 128.7 (CH), 128.3 (CH), 127.6 (CH), 127.3 (CH), 127.0 (CH), 125.4 (CH), 122.9 (d, J_{C-F}=1.8 Hz, C),

115.9 (d, J_{C-F}=9.7 Hz, CH), 105.2 (d, J_{C-F}=23.6 Hz, CH), 102.1 (d, J_{C-F}=27.8 Hz, CH), 66.0 (CH), 54.0 (CH_2), 52.3 (CH), 34.4 (C), 30.3 (CH_3); **HRMS (ESI/Q-TOF)** *m/z* [M + H]$^+$ calcd for $C_{36}H_{40}FN_2O_2^+$ 551.3068, found 551.3071. **FTIR (neat)** cm^{-1} : 3630 (w), 2955 (m), 1684 (s), 1613 (m), 1326 (s), 1114 (s), 697 (s).

4-Benzyl-3-((3,5-di-*tert*-butyl-4-hydroxyphenyl)(phenyl)methyl)-1-methyl-3,4-dihydro-quinoxalin-2(1*H*)-one (3ha)

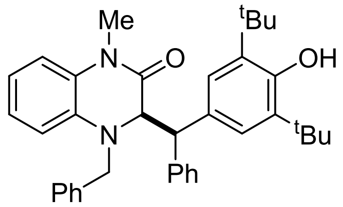

Using 4-benzyl-1-methyl-3,4-dihydroquinoxalin-2(1*H*)-one (**1h**, 37.8 mg, 0.15 mmol, 1.5 equiv.) and 4-benzylidene- 2,6-di-*tert*-butylcyclohexa-2,5-dien-1-one (**2a**, 29.4 mg, 0.1 mmol, 1 equiv.), according to GP-1, compound **3ha** (48.7 mg, 0.089 mmol, 89% yield, yellow oil) was obtained as a mixture of diastereomers (1:1 dr) that cannot be separated by column chromatography using hexane:EtOAc mixtures (from 9:1 to 8:2).

^{1}H-NMR (300 MHz, $CDCl_3$) δ 7.41–7.37 (m, 2H), 7.33–7.27 (m, 5H), 7.25–7.17 (m, 9H), 7.10 (dd, *J*=7.3, 2.3 Hz, 2H), 7.03–6.98 (m, 6H), 6.96-6.91 (m, 2H), 6.87–6.80 (m, 3H), 6.74 (dd, *J*=7.9, 1.5 Hz, 1H), 6.67 (dd, *J*=8.0, 1.4 Hz, 1H), 6.56 (d, *J*=7.7 Hz, 1H), 5.18 (s, 1H), 4.99 (s, 1H), 4.67 (d, *J*=6.0 Hz, 1H), 4.61 (d, *J*=9.9 Hz, 1H), 4.38 (d, *J*=15.1 Hz, 1H), 4.06 (d, *J*=6.0 Hz, 1H), 4.00 (d, *J*=6.4 Hz, 1H), 3.97–3.85 (m, 2H), 3.58 (d, *J*=15.2 Hz, 1H), 3.29 (s, 3H), 3.19 (s, 3H), 1.43 (s, 18H), 1.33 (s, 18H). **^{13}C{^{1}H}-NMR (75 MHz, $CDCl_3$)** δ 164.9 (C), 164.7 (C), 153.0 (C), 152.3 (C), 142.9 (C), 140.2 (C), 137.0 (C), 136.6 (C), 136.0 (C), 134.97 (C), 134.95 (C), 134.8 (C), 131.9 (C), 130.6 (C), 130.5 (C), 130.2 (C), 128.7 (CH), 128.6 (CH), 128.49 (CH), 128.46 (CH), 128.41 (CH), 128.1 (CH), 127.7 (CH), 127.4 (CH), 127.29 (CH), 127.27 (CH), 126.8 (CH), 126.4 (CH), 125.6 (CH), 125.4 (CH), 123.4 (CH), 123.3 (CH), 119.24 (CH), 119.18 (CH), 114.7 (CH), 114.6 (CH), 114.2 (CH), 114.0 (CH), 66.9 (CH), 66.5 (CH), 53.9 (CH_2), 53.5 (CH_2), 53.1 (CH), 51.9 (CH), 34.4 (C), 34.1 (C), 30.3 (CH_3), 30.2 (CH_3), 29.4 (CH_3), 29.1 (CH_3); **HRMS (ESI/Q-TOF)** *m/z* [M + H]$^+$ calcd for $C_{37}H_{43}N_2O_2^+$ 547.3319, found 547.3327. **FTIR (neat)** cm^{-1} : 3628 (w), 2952 (m), 1682 (s), 1610 (m), 747 (s), 697 (s).

1,4-Dibenzyl-3-((3,5-di-*tert*-butyl-4-hydroxyphenyl)(phenyl)methyl)-7-(trifluoromethyl)-3,4-dihydroquinoxalin-2(1*H*)-one (3ia)

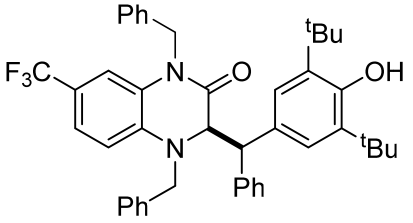

Using 1,4-dibenzyl-7-(trifluoromethyl)-3,4-dihydroquinoxalin-2(1*H*)-one (**1i**, 59.5 mg, 0.15 mmol, 1.5 equiv.) and 4-benzylidene-2,6-di-*tert*-butylcyclohexa- 2,5-dien-1-one (**2a**, 29.4 mg, 0.1 mmol, 1 equiv.), according to GP-1, compound **3ia** (50.8 mg, 0.074 mmol, 74% yield) was obtained as a mixture of diastereomers (1.2:1 dr) that were separated by column chromatography using 95:5 hexane:EtOAc mixture.

Characterization of **3ia'**: yellow oil; **^{1}H-NMR (500 MHz, $CDCl_3$)** δ 7.37–7.13 (m, 12H), 7.10–6.93 (m, 7H), 6.62 (d, *J*=8.4 Hz, 1H), 5.15 (d, *J*=15.9 Hz, 1H), 5.10 (s, 1H), 4.92 (d, *J*=15.9 Hz, 1H), 4.88 (d, *J*=8.5 Hz, 1H), 4.17 (d, *J*=15.6 Hz, 1H), 4.10 (d, *J*=8.5 Hz, 1H), 3.79 (d, *J*=15.6 Hz, 1H), 1.39 (s, 18H); **^{19}F{^{1}H}-NMR (471 MHz, $CDCl_3$)** δ -61.26; **^{13}C{^{1}H}-NMR (126 MHz, $CDCl_3$)** δ 164.1 (C), 152.7 (C), 142.3 (C), 137.6 (C), 136.3 (C), 136.1 (C), 135.4 (C), 129.7 (C), 129.4 (C), 128.7 (CH), 128.7 (CH), 128.6 (CH), 127.6 (CH), 127.3 (CH), 127.3 (CH), 127.0 (CH), 126.7 (2CH), 125.6 (CH), 124.5 (C, q, J_{C-F}=273.8 Hz), 120.9 (CH, q, J_{C-F}=3.6 Hz), 120.6 (C, q, J_{C-F}=32.9 Hz), 113.7 (CH), 112.4 (CH, q, J_{C-F}=3.9 Hz), 67.2 (CH), 54.1 (CH_2), 53.1 (CH), 46.3 (CH_2), 34.3 (C), 30.3 (CH_3); **HRMS (ESI/Q-TOF)** *m/z* $[M + H]^+$ calcd for $C_{44}H_{46}F_3N_2O_2^+$ 691.3506, found 691.3509. **FTIR (neat)** cm^{-1} : 3630 (w), 2955 (m), 1684 (s), 1610 (m), 1453 (s), 1259 (s), 1114 (s), 697 (s).

Characterization of **3ia"**: yellow oil; **^{1}H-NMR (500 MHz, $CDCl_3$)** δ 7.41 (d, *J*= 7.5 Hz, 2H), 7.32 (t, *J*=7.6 Hz, 2H), 7.26–7.13 (m, 9H), 7.07–6.88 (m, 6H), 6.53 (d, *J*= 8.2 Hz, 1H), 5.52 (d, *J*=15.8 Hz, 1H), 5.20 (s, 1H), 4.78 (d, *J*=10.7 Hz, 1H), 4.59 (d, *J*= 15.8 Hz, 1H), 3.99 (d, *J*=10.7 Hz, 1H), 3.92 (d, *J*=15.5 Hz, 1H), 3.53 (d, *J*=15.5 Hz, 1H), 1.40 (s, 18H); **^{19}F{^{1}H}-NMR (471 MHz, $CDCl_3$)** δ -61.26; **^{13}C{^{1}H}-NMR (126 MHz, $CDCl_3$)** δ 164.1 (C), 153.2 (C), 139.5 (C), 137.5 (C), 136.5 (C), 136.4 (C), 136.2 (C), 131.4 (C), 129.8 (C), 128.9 (CH), 128.8 (CH), 128.7 (CH), 128.3 (CH), 127.7 (CH), 127.4 (CH), 127.3 (CH), 127.1 (CH), 126.8 (CH), 126.1 (C, J_{C-F} = 271.0 Hz), 125.2 (CH), 120.8 (C, q, J_{C-F} = 32.6 Hz), 120.8 (CH, q, J_{C-F} = 3.6 Hz), 114.1 (CH), 112.4 (CH, q, J_{C-F} = 3.7 Hz), 66.5 (CH), 54.2 (CH_2), 52.1 (CH), 46.6 (CH_2), 34.4 (C), 30.3 (CH_3); **HRMS (ESI/Q-TOF)** *m/z* $[M + H]^+$ calcd for$C_{44}H_{46}F_3N_2O_2^+$ 691.3506, found 691.3511. **FTIR (neat)** cm^{-1} : 3630 (w), 2955 (m), 1684 (s), 1610 (m), 1453 (s), 1259 (s), 1110 (s), 697 (s).

4-Benzyl-3-((3,5-di-*tert*-butyl-4-hydroxyphenyl)(2-methoxyphenyl)methyl)-3,4-dihydroquinoxalin-2(1*H*)-one (3ab)

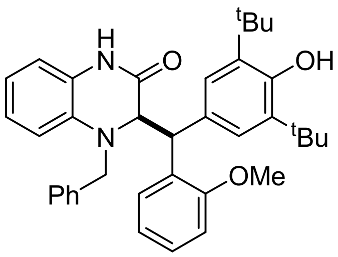

Using 4-benzyl-3,4-dihydroquinoxalin-2-one (**1a**, 38.4 mg, 0.15 mmol, 1.5 equiv.) and 2,6-di-*tert*-butyl-4-(2-methoxybenzylidene)cyclohexa-2,5-dien-1-one (**2b**, 32.4 mg, 0.1 mmol, 1 equiv.), according to GP-1, compound **3ab** (54.6 mg, 0.097 mmol, 97% yield) was obtained as a mixture of diastereomers (1.5:1 dr) that were separated by column chromatography using hexane:EtOAc mixtures (from 9:1 to 8:2).

Characterization of **3ab'**: yellow oil; **^{1}H-NMR (300 MHz, CDCl$_3$)** δ 8.22 (s, 1H), 7.42 (dd, *J*=7.6, 1.7 Hz, 1H), 7.25–7.16 (m, 4H), 7.10 (dd, *J*=7.6, 1.9 Hz, 2H), 7.05 (s, 2H), 6.91 (td, *J*=7.5, 1.2 Hz, 1H), 6.87–6.79 (m, 2H), 6.69 (td, *J*=7.5, 1.3 Hz, 1H), 6.57 (dd, *J*=7.7, 1.5 Hz, 1H), 6.49 (d, *J*=8.3 Hz, 1H), 4.94 (s, 1H), 4.82 (dd, *J*=7.4, 0.9 Hz, 1H), 4.53 (d, *J*=7.4 Hz, 1H), 4.29 (d, *J*=15.5 Hz, 1H), 4.05 (d, *J*=15.6 Hz, 1H), 3.67 (s, 3H), 1.32 (s, 18H); **^{13}C{^{1}H}-NMR (75 MHz, CDCl$_3$)** δ 166.2 (C), 157.0 (C), 152.2 (C), 137.3 (C), 134.7 (C), 133.8 (C), 130.7 (C), 130.2 (CH), 129.6 (C), 128.4, (CH), 127.7 (CH), 127.4, (CH) 127.1 (CH), 126.8 (C), 126.2, (CH) 123.5 (CH), 120.7 (CH), 118.8 (CH), 114.9 (CH), 114.1 (CH), 110.7 (CH), 66.0 (CH), 55.2 (CH), 53.3 (CH$_2$), 47.0 (CH$_3$), 34.2 (C), 30.3 (CH$_3$); **HRMS (ESI/Q-TOF)** *m/z* $[M + H]^+$ calcd for $C_{37}H_{43}N_2O_3^+$ 563.3268, found 563.3267. **FTIR (neat)** cm^{-1} : 3626 (w), 2955 (m), 1673 (s), 1610 (m), 1435 (s), 1110 (s), 697 (s).

Characterization of **3ab"**: yellow oil; **^{1}H-NMR (300 MHz, CDCl$_3$)** δ 8.57 (s, 1H), 7.44 (dd, *J*=7.6, 1.3 Hz, 1H), 7.24–7.18 (m, 2H), 7.16–7.11 (m, 1H), 7.10 (s, 2H), 7.06–6.98 (m, 2H), 6.96 (dd, *J*=7.5, 1.6 Hz, 1H), 6.89 (td, *J*=7.9, 1.4 Hz, 1H), 6.80 (td, *J*=7.5, 1.2 Hz, 1H), 6.76–6.67 (m, 2H), 6.48 (d, *J*=7.8 Hz, 1H), 5.16 (s, 1H), 4.76 (d, *J*=10.4 Hz, 1H), 4.42 (d, *J*=10.5 Hz, 1H), 3.96 (d, *J*=15.5 Hz, 1H), 3.65 (s, 3H), 3.53 (d, *J*=15. Hz, 1H), 1.43 (s, 18H); **^{13}C{^{1}H}-NMR (75 MHz, CDCl$_3$)** δ 166.3 (C), 157.3 (C), 152. (C), 137.4 (C), 135.8 (C), 133.5 (C), 131.8 (C), 129.8 (CH), 128.6 (C), 128.4 (CH), 127.8(CH), 127.4 (CH), 127.2 (CH), 127.0 (C), 126.1 (CH), 123.6 (CH), 120.2 (CH), 118.8(CH), 115.3 (CH), 114.6 (CH), 110.9 (CH), 65.4 (CH), 55.2 (CH+CH$_3$), 54.0 (CH$_2$), 34.4 (C), 30.4 (CH$_3$); **HRMS (ESI/Q-TOF)** *m/z* $[M + H]^+$ calcd for $C_{37}H_{43}N_2O_3^+$ 563.3268, found 563.3271. **FTIR (neat)** cm^{-1} : 3630 (w), 2952 (m), 1677 (s), 1610 (m), 1235 (m), 742 (s).

4-Benzyl-3-((3,5-di-*tert*-butyl-4-hydroxyphenyl)(3-methoxyphenyl)methyl)-3,4-dihydroquinoxalin-2(1*H*)-one (3ac)

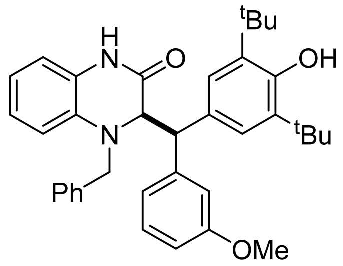

Using 4-benzyl-3,4-dihydroquinoxalin-2-one (**1a**, 38.4 mg, 0.15 mmol, 1.5 equiv.) and 2,6-di-*tert*-butyl-4-(3-methoxybenzylidene)cyclohexa-2,5-dien-1-one (**2c**, 32.4 mg, 0.1 mmol, 1 equiv.), according to GP-1, compound **3ac** (54.6 mg, 0.097 mmol, 97% yield) was obtained as a mixture of diastereomers (1.4:1 dr) that were separated by column chromatography using hexane:EtOAc mixtures (from 9:1 to 8:2).

Characterization of **3ac'**: yellow oil; **^{1}H-NMR (300 MHz, $CDCl_3$)** δ 8.41 (s, 1H), 7.25–7.13 (m, 4H), 7.07 (dd, *J*=7.6, 1.9 Hz, 3H), 6.93 (td, *J*=7.7, 1.4 Hz, 2H), 7.01 (s, 2H), 6.89–6.83 (m, 2H), 6.82–6.69 (m, 2H), 6.67–6.56 (m, 2H), 5.00 (s, 1H), 4.58 (dd, *J*=8.1, 0.9 Hz, 1H), 4.28 (d, *J*=15.4 Hz, 1H), 4.05 (d, *J*=8.1 Hz, 1H), 3.78 (d, *J*=15.3 Hz, 1H), 3.75 (s, 1H), 1.34 (s, 18H). **^{13}C{^{1}H}-NMR (75 MHz, $CDCl_3$)** δ 165.6 (C), 159.6 (C), 152.5 (C), 143.9 (C), 136.9 (C), 135.1 (C), 133.7 (C), 129.6 (C), 129.4 (CH), 128.5 (CH), 127.53 (CH), 127.3 (CH), 126.9 (C), 125.6 (CH), 123.8 (CH), 121.2 (CH), 119.1 (CH), 115.2 (CH), 114.5 (CH), 114.1 (CH), 112.7 (CH), 66.6 (CH), 55.1 (CH), 53.9 (CH_2), 52.9 (CH_3), 34.2 (C), 30.3 (CH_3); **HRMS (ESI/Q-TOF)** *m/z* $[M + H]^+$ calcd for $C_{37}H_{43}N_2O_3^+$ 563.3268, found 563.3264. **FTIR (neat)** cm^{-1} : 3630 (m), 3358 (w), 2955 (m), 1692 (s), 1606 (m), 1435 (m), 1252 (m), 1114 (s), 742 (s), 697 (s).

Characterization of **3ac"**: yellow oil; **^{1}H-NMR (300 MHz, $CDCl_3$)** δ 8.25 (s, 1H), 7.24–7.15 (m, 4H), 7.03–6.91 (m, 6H), 6.86 (t, *J*=2.1 Hz, 1H), 6.82 (td, *J*=7.6, 1.2 Hz, 1H), 6.73–6.66 (m, 2H), 6.51 (dd, *J*=8.1, 1.2 Hz, 1H), 5.18 (s, 1H), 4.49 (dd, *J*=10.4, 1.0 Hz, 1H), 4.03–3.90 (m, 2H), 3.71 (s, 3H), 3.49 (d, *J*=15.4 Hz, 1H), 1.42 (s, 18H).**^{13}C{^{1}H}-NMR (75 MHz, $CDCl_3$)** δ 165.5 (C), 159.3 (C), 153.1 (C), 141.5 (C), 137.1 (C), 136.1 (C), 133.5 (C), 131.4 (C), 129.1 (CH), 128.6 (CH), 127.4 (CH), 127.4 (CH), 126.9 (C), 125.5 (CH), 123.9 (CH), 121.0 (CH), 119.2 (CH), 115.5 (CH), 115.1 (CH), 114.9 (CH), 112.1 (CH), 66.4 (CH), 55.1 (CH), 54.1 (CH_2), 52.2 (CH_3), 34.4 (C), 30.4 (CH_3); **HRMS (ESI/Q-TOF)** *m/z* $[M + H]^+$ calcd for $C_{37}H_{43}N_2O_3^+$ 563.3268, found 563.3268. **FTIR (neat)** cm^{-1} : 3615 (s), 2952 (m), 1684 (s), 1606 (m), 1256 (m), 741 (s), 697 (s).

4-Benzyl-3-((3,5-di-*tert*-butyl-4-hydroxyphenyl)(4-methoxyphenyl)methyl)-3,4-dihydroquinoxalin-2(1*H*)-one (3ad)

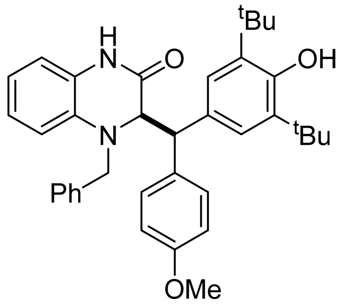

Using 4-benzyl-3,4-dihydroquinoxalin-2-one (**1a**, 38.4 mg, 0.15 mmol, 1.5 equiv.) and 2,6-di-*tert*-butyl-4-(4-methoxybenzylidene)cyclohexa-2,5-dien-1-one (**2d**, 32.4 mg, 0.1 mmol, 1 equiv.), according to GP-1, compound **3ac** (48.4 mg, 0.086 mmol, 86% yield) was obtained as a mixture of diastereomers (1:1 dr) that were separated by column chromatography using hexane:EtOAc mixtures (from 9:1 to 8:2).

Characterization of **3ad'**: yellow oil; **^{1}H-NMR (300 MHz, CDCl$_3$)** δ 8.27 (s, 1H), 7.25–7.19 (m, 3H), 7.17 (d, *J*=8.7 Hz, 2H), 7.07 (dd, *J*=7.6, 1.9 Hz, 2H), 6.99 (s, 2H), 6.92 (td, *J*=7.7, 1.5 Hz, 1H), 6.83 (d, *J* = 8.7 Hz, 2H), 6.74 (td, *J*=7.5, 1.3 Hz, 1H), 6.60 (dd, *J*=7.7, 1.4 Hz, 2H), 4.99 (s, 1H), 4.54 (dd, *J*=8.1, 0.9 Hz, 1H), 4.27 (d, *J*=15.4 Hz, 1H), 4.04 (d, *J*=8.1 Hz, 1H), 3.81-3.76 (m, 4H), 1.34 (s, 18H). **^{13}C{^{1}H}-NMR (75 MHz, CDCl$_3$)** δ 165.8 (C), 158.4 (C), 152.4 (C), 137.0 (C), 135.1 (C), 134.4 (C), 133.7 (C), 132.2 (CH), 129.8 (CH), 128.5 (CH), 127.5 (CH), 127.3 (C), 126.8 (CH), 125.5 (CH), 123.85 (CH), 119.0 (CH), 115.2 (CH), 114.41 (CH), 114.37 (CH), 113.8 (CH), 66.9 (CH), 55.3 (CH), 54.0 (CH$_2$), 52.0 (CH$_3$), 34.2 (C), 30.3 (CH$_3$); **HRMS (ESI/Q-TOF)** *m/z* [M + H]$^+$ calcd for $C_{37}H_{43}N_2O_3^+$ 563.3268, found 563.3266. **FTIR (neat)** cm^{-1} : 3630 (w), 2952 (m), 1684 (s), 1595 (s), 1505 (s), 1252 (m), 1174 (s).

Characterization of **3ad"**: yellow oil; **^{1}H-NMR (300 MHz, CDCl$_3$)** δ 8.16 (s, 1H), 7.28 (d, *J*=2.1 Hz, 1H), 7.26–7.17 (m, 3H), 7.01–6.92 (m, 6H),6.84 (dd, *J*=7.6, 1.3 Hz, 1H), 6.79 (d, *J*=8.8 Hz, 2H), 6.70 (dd, *J*=7.7, 1.5 Hz, 1H), 6.52 (dd, *J*=8.1, 1.2 Hz, 1H), 5.17 (s, 1H), 4.45 (dd, *J*=10.6, 1.0 Hz, 1H), 4.06–3.84 (m, 2H), 3.71 (s, 3H), 3.46 (d, *J*=15.3 Hz, 1H), 1.42 (s, 18H). **^{13}C{^{1}H}-NMR (75 MHz, CDCl$_3$)** δ 165.6 (C), 158.3 (C), 153.0 (C), 137.1 (C), 136.1 (C), 133.5 (C), 132.2 (C), 132.0 (C), 129.6 (CH), 128.5 (CH), 127.4 (CH), 127.3 (CH), 126.9 (C), 125.3 (CH), 123.9 (CH), 119.2(CH), 115.4 (CH), 114.9 (CH), 113.6 (CH), 66.4 (CH) , 55.1 (CH), 53.9 (CH$_2$), 51.2 (CH$_3$), 34.4 (C), 30.3 (CH$_3$); **HRMS (ESI/Q-TOF)** *m/z* [M + H]$^+$ calcd for $C_{37}H_{43}N_2O_3$ $^+$ 563.3268, found 563.3265. **FTIR (neat)** cm^{-1} : 3630 (w), 2952 (m), 1684 (s), 1595 (s), 1505 (s), 1252 (s), 745 (m), 697 (m).

4-Benzyl-3-((2-bromophenyl)(3,5-di-*tert*-butyl-4-hydroxyphenyl)methyl)-3,4-dihydro-quinoxalin-2(1*H*)-one (3ae)

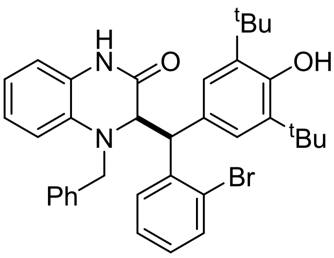

Using 4-benzyl-3,4-dihydroquinoxalin-2-one (**1a**, 38.4 mg, 0.15 mmol, 1.5 equiv.) and 4-(2-bromobenzylidene)- 2,6-di-*tert*-butylcyclohexa-2,5-dien-1-one (**2e**, 37.3 mg, 0.1 mmol, 1 equiv.), according to GP-1, compound **3ae** (54.4 mg, 0.089 mmol, 89% yield, yellow oil) was obtained as a mixture of diastereomers (1.4:1 dr) that cannot be separated by column chromatography using hexane:EtOAc mixtures (from 9:1 to 8:2).

^{1}H-NMR (300 MHz, $CDCl_3$) δ 8.45 (s, 1H), 8.38 (s, 1H), 7.92 (dd, *J*=7.9, 1.6 Hz, 1H), 7.75 (dd, *J*=7.9, 1.6 Hz, 1H), 7.51 (dd, *J*=8.0, 1.3 Hz, 1H), 7.39 (dd, *J*=8.0, 1.3 Hz, 1H), 7.31 (td, *J*=7.6, 1.4 Hz, 3H), 7.25–7.16 (m, 4H), 7.12 (dd, *J*=6.9, 2.6 Hz, 2H), 7.10–7.06 (m, 1H), 7.03–6.99 (m, 4H), 6.99–6.94 (m, 3H), 6.94–6.89 (m, 2H), 6.85 (td, *J*=7.5, 1.2 Hz, 1H), 6.78 (d, *J*=1.5 Hz, 1H), 6.77–6.74 (m, 1H), 6.71 (d, *J*=7.3 Hz, 2H), 6.54 (d, *J*=7.9 Hz, 1H), 6.45 (dd, *J*=8.0, 1.4 Hz, 1H), 5.20 (sa, 1H), 4.97 (sa, 1H), 4.74 (d, *J*=5.3 Hz, 1H), 4.63 (d, *J*=10.7 Hz, 1H), 4.58–4.44 (m, 3H), 4.07 (d, *J*=14.9 Hz, 1H), 4.00 (d, *J*=15.3 Hz, 1H), 3.48 (d, *J*=15.2 Hz, 1H), 1.42 (s, 18H), 1.28 (s, 18H). **$^{13}C\{^1H\}$-NMR (75 MHz, $CDCl_3$)** δ 166.2 (C), 165.4 (C), 153.1 (C), 152.4 (C), 142.0 (C), 138.9 (C), 137.1 (C), 136.4 (C), 136.0 (C), 134.9 (C), 133.8 (C), 133.4 (C), 133.0 (CH), 132.9 (CH), 130.02 (CH), 129.96 (C), 129.6 (CH), 128.6 (CH), 128.5 (CH), 128.3 (C), 128.2 (CH), 127.9 (CH), 127.8 (CH), 127.7 (CH), 127.41 (CH), 127.37 (CH), 127.0 (C), 126.92 (CH), 126.88 (C), 126.1 (CH), 125.9 (CH), 125.0 (C), 123.8 (CH), 119.5 (CH), 119.4 (CH), 115.6 (CH), 115.0 (CH), 114.9 (CH), 114.3 (CH), 66.3 (CH), 65.5 (CH), 54.0 (CH_2), 53.3 (CH_2), 51.7 (CH), 49.3 (CH), 34.4 (C), 34.1 (C), 30.3 (CH_3), 30.1 (CH_3); **HRMS (ESI/Q-TOF)** *m/z* $[M + H]^+$ calcd for $C_{36}H_{40}BrN_2O_2{}^+$ 611.2268, found 611.2271. **FTIR (neat)** cm^{-1} : 3630 (w), 2959 (m), 1669 (s), 1613 (s), 1021 (m), 749 (s), 693 (s).

4-Benzyl-3-((4-chlorophenyl)(3,5-di-*tert*-butyl-4-hydroxyphenyl)methyl)-3,4-dihydro-quinoxalin-2(1*H*)-one (3af)

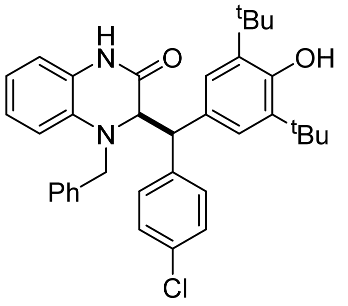

Using 4-benzyl-3,4-dihydroquinoxalin-2-one (**1a**, 38.4 mg, 0.15 mmol, 1.5 equiv.) and 2,6-di-*tert*-butyl-4-(4-chlorobenzylidene)cyclohexa-2,5-dien-1-one (**2f**, 32.8 mg, 0.1 mmol, 1 equiv.), according to GP-1, compound **3af** (54.4 mg, 0.096 mmol, 96% yield) was obtained as a mixture of diastereomers (1:1 dr) that were separated by column chromatography using hexane:EtOAc mixtures (from 9:1 to 8:2).

Characterization of **3af'**: yellow oil; **^{1}H-NMR (300 MHz, CDCl$_3$)** δ 8.26 (s, 1H), 7.28–7.15 (m, 7H), 7.08 (dd, *J*=7.4, 2.0 Hz, 2H), 6.99–6.89 (m, 3H), 6.76 (td, *J*=7.6, 1.3 Hz, 1H), 6.66 (d, *J*=8.0 Hz, 1H), 6.59 (dd, *J*=7.7, 1.5 Hz, 1H), 5.03 (s, 1H), 4.54 (dd, *J*=7.6, 0.9 Hz, 1H), 4.35 (d, *J*=15.2 Hz, 1H), 4.06 (d, *J*=7.6 Hz, 1H), 3.82 (d, *J*=15.2 Hz, 1H), 1.33 (s, 18H). **^{13}C{^{1}H}-NMR (75 MHz, CDCl$_3$)** δ 165.4 (C), 152.7 (C), 144.4 (C), 136.6 (C), 135.3 (C), 134.1 (C), 133.6 (C), 129.7 (CH), 129.2 (CH), 129.0 (C), 128.6 (CH), 127.7 (CH), 127.5 (CH), 126.9 (CH), 125.7 (CH), 124.0 (CH), 119.5 (CH), 115.2 (CH), 114.7 (CH), 66.1 (CH), 54.2 (CH$_2$), 52.7 (CH), 34.3 (C), 30.2 (CH$_3$); **HRMS (ESI/Q-TOF)** *m/z* [M + H]$^+$ calcd for $C_{36}H_{40}ClN_2O_2^+$ 567.2773, found 567.2770. **FTIR (neat)** cm^{-1} : 3623 (w), 2955 (m), 1688 (s), 1505 (m), 1431 (m), 741 (s), 700 (s).

Characterization of **3af''**: yellow oil; **^{1}H-NMR (300 MHz, CDCl$_3$)** δ 8.28 (s, 1H), 7.37–7.30 (m, 1H), 7.25–7.09 (m, 6H), 7.02–6.92 (m, 5H), 6.84 (td, *J*=7.6, 1.2 Hz, 1H), 6.69 (dd, *J*=7.7, 1.5 Hz, 1H), 6.53 (d, *J*=7.9 Hz, 1H), 5.22 (s, 1H), 4.47 (dd, *J*=10.4, 1.1 Hz, 1H), 4.04–3.84 (m, 2H), 3.46 (d, *J*=15.3 Hz, 1H), 1.42 (s, 18H). **^{13}C{^{1}H}-NMR (75 MHz, CDCl$_3$)** δ 165.4 (C), 153.3 (C), 142.04 (C), 136.9 (C), 136.3 (C), 133.9 (C), 133.3 (C), 130.8 (C), 129.3 (CH), 128.6 (CH), 127.4 (CH), 127.4 (CH), 127.1 (CH), 126.7 (C), 126.6 (CH), 125.4 (CH), 124.0 (CH), 119.4 (CH), 115.6 (CH), 114.9 (CH), 65.9 (CH), 54.0 (CH$_2$), 51.7 (CH), 34.4 (C), 30.3 (CH$_3$); **HRMS (ESI/Q-TOF)** *m/z* [M + H]$^+$ calcd for $C_{36}H_{40}ClN_2O_2^+$ 567.2773, found 567.2775. **FTIR (neat)** cm^{-1} : 3630 (w), 2955 (m), 1673 (s), 1610 (s), 745 (s), 697 (s).

3-((1-Benzyl-3-oxo-1,2,3,4-tetrahydroquinoxalin-2-yl)(3,5-di-tert-butyl-4-hydroxyphenyl)methyl)benzonitrile (3ag)

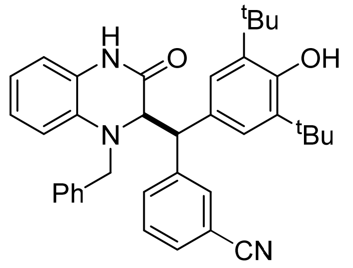

Using 4-benzyl-3,4-dihydroquinoxalin-2-one (**1a**, 38.4 mg, 0.15 mmol, 1.5 equiv.) and 3-((3,5-di-*tert*-butyl-4-oxocyclohexa-2,5-dien-1-ylidene)methyl)benzonitrile (**2g**, 31.9 mg, 0.1 mmol, 1 equiv.), according to GP-1, compound **3ag** (54.8 mg, 0.098 mmol, 98% yield) was obtained as a mixture of diastereomers (1:1 dr) that can be separated by column chromatography using hexane:EtOAc mixtures (from 9:1 to 8:2).

Characterization of **3ag"**: yellow oil, m.p.: 206-210 ºC; **^{1}H NMR (500 MHz, CDCl$_3$)** δ 8.71 (s, 1H), 7.58 (d, *J*=7.9 Hz, 1H), 7.55–7.49 (m, 2H), 7.36 (t, *J*=7.7 Hz, 1H), 7.30–7.21 (m, 3H), 7.15–7.06 (m, 2H), 6.95 (t, *J*=7.4 Hz, 1H), 6.89 (s, 2H), 6.75 (t, *J*=7.4 Hz, 1H), 6.69 (d, *J*=8.0 Hz, 1H), 6.55 (d, *J*=7.4 Hz, 1H), 5.05 (s, 1H), 4.54 (d, *J*=6.5 Hz, 1H), 4.42 (d, *J*=15.0 Hz, 1H), 4.15 (d, *J*=6.5 Hz, 1H), 3.93 (d, *J*=15.0 Hz, 1H), 1.31 (s, 18H); **^{13}C{^{1}H} NMR (126 MHz, CDCl$_3$)** δ 165.7 (C), 152.8 (C), 143.8 (C), 136.2 (C), 135.5 (C), 133.5 (C), 133.2 (CH), 132.6 (CH), 130.2 (CH), 129.1 (CH), 128.7 (CH), 128.4 (C), 127.8 (CH), 127.7 (CH), 126.9 (C), 125.7 (CH), 124.1 (CH), 119.9 (CH), 118.8 (C), 115.2 (CH), 114.8 (CH), 112.3 (C), 65.6 (CH), 54.5 (CH$_2$), 52.7 (CH), 34.2 (C), 30.1 (CH$_3$); **HRMS (ESI/Q-TOF)** *m/z* [M + H]$^+$ calcd for $C_{37}H_{40}N_3O_2^+$ 558.3115, found 558.3118. **FTIR (neat)** cm^{-1} : 3615 (w), 2959 (m), 2232 (m), 1684 (s), 1505 (s), 742 (s).

Characterization of **3ag"**: yellow solid, m.p.: 177-182 ºC; **^{1}H NMR (500 MHz, CDCl$_3$)** δ 8.73 (s, 1H), 7.64 (d, *J*=7.9 Hz, 1H), 7.57 (s, 1H), 7.41 (d, *J*=7.7 Hz, 1H), 7.32 (t, *J*=7.8 Hz, 1H), 7.25–7.20 (m, 3H), 7.05–6.99 (m, 1H), 7.00–6.96 (m, 2H), 6.93 (s, 2H), 6.87 (t, *J*=7.5 Hz, 1H), 6.73 (d, *J*=9.3 Hz, 1H), 6.57 (d, *J*=8.0 Hz, 1H), 5.26 (s, 1H), 4.49 (d, *J*=10.2 Hz, 1H), 4.05 (d, *J* = 15.1 Hz, 1H), 4.00 (d, *J*=10.2 Hz, 1H), 3.48 (d, *J*=15.1 Hz, 1H), 1.43 (s, 18H); **^{13}C{^{1}H} NMR (126 MHz, CDCl$_3$)** δ 165.5 (C), 153.4 (C), 141.6 (C), 136.7 (C), 136.5 (C), 133.2 (C), 133.0 (CH), 132.7 (CH), 130.5 (CH), 130.3 (C), 128.8 (CH), 128.6 (CH), 127.5 (CH), 127.4 (CH), 126.6 (C), 125.3 (CH), 124.2 (CH), 119.6 (CH), 118.8 (C), 115.7 (CH), 115.0 (CH), 112.1 (C), 65.4 (CH), 54.0 (CH$_2$), 51.5 (CH), 34.4 (C), 30.3 (CH$_3$); **HRMS (ESI/Q-TOF)** *m/z* [M + H]$^+$ calcd for $C_{37}H_{40}N_3O_2^+$ 558.3115, found 558.3116. **FTIR (neat)** cm^{-1} : 3619 (w), 2955 (m), 2228 (w), 1677 (s), 1431 (m), 727 (s), 693 (s).

4-Benzyl-3-((3,5-di-*tert*-butyl-4-hydroxyphenyl)(4-nitrophenyl)methyl)-3,4-dihydro-quinoxalin-2(1*H*)-one (3ah)

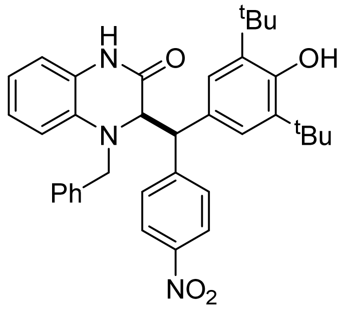

Using 4-benzyl-3,4-dihydroquinoxalin-2-one (**1a**, 38.4 mg, 0.15 mmol, 1.5 equiv.) and 2,6-di-*tert*-butyl-4-(4-nitrobenzylidene)cyclohexa-2,5-dien-1-one (**2h**, 33.9 mg, 0.1 mmol, 1 equiv.), according to GP-1, compound **3ah** (57.6 mg, 0.099 mmol, 99% yield, orange oil) was obtained as a mixture of diastereomers (1:1 dr) that cannot be separated by column chromatography using hexane:EtOAc mixtures (from 9:1 to 8:2).

^{1}H-NMR (300 MHz, $CDCl_3$) δ 9.24 (s, 1H), 9.12 (s, 1H), 8.12 (d, *J*=8.8 Hz, 2H), 8.06 (d, *J*=8.8 Hz, 2H), 7.52–7,44(m, 4H), 7.26–7.22 (m, 6H), 7.14–7.07 (m, 2H), 7.05–6.94 (m, 4H), 6.93 (d, *J*=6.5 Hz, 4H), 6.85 (td, *J*=7.6, 1.2 Hz, 1H), 6.7 (m, *J*=8.0, 1.2 Hz, 3H), 6.58 (dd, *J*=8.2, 1.2 Hz, 1H), 6.47 (dd, *J*=7.7, 1.4 Hz, 1H), 5.28 (s, 1H), 5.06 (s, 1H), 4.61 (d, *J*=6.6 Hz, 1H), 4.54 (dd, *J*=10.4, 0.9 Hz, 1H), 4.44 (d, *J*=15.0 Hz, 1H), 4.24 (d, *J*=6.6 Hz, 1H), 4.07 (dd, *J*=12.8, 2.4 Hz, 2H), 3.97 (d, *J*=15.0 Hz, 1H), 3.47 (d, *J*=15.1 Hz, 1H), 1.43 (s, 18H), 1.31 (s, 18H).**^{13}C{^{1}H}-NMR (75 MHz, $CDCl_3$)** δ 165.8 (C), 165.7 (C), 153.5 (C), 152.9 (C), 150.0 (C), 147.7 (C), 146.6 (C), 146.5 (C), 136.7 (C), 136.5 (C), 136.3 (C), 135.5 (C), 133.4 (C), 133.1 (C), 129.9 (C), 129.63 (CH), 129.58 (CH), 128.7 (CH), 128.6 (CH), 128.2 (C), 127.8 (CH), 127.7 (CH), 127.6 (CH), 127.4 (CH), 126.9 (C), 126.6 (C), 125.7 (CH), 125.4 (CH), 124.2 (CH), 124.1 (CH), 123.5 (CH), 123.23 (CH), 119.8 (CH), 119.6 (CH), 115.7 (CH), 115.3(CH), 115.0 (CH), 114.8 (CH), 65.4 (CH), 65.3 (CH), 54.5 (CH_2), 54.0 (CH_2), 53.0 (CH), 51.8 (CH), 34.4 (C), 34.2 (C), 30.2 (CH_3), 30.1 (CH_3); **HRMS (ESI/Q-TOF)** *m/z* $[M + H]^+$ calcd for $C_{36}H_{40}N_3O_4^+$ 578.3013, found 578.3015. **FTIR (neat)** cm^{-1} : 3630 (w), 2955 (m), 1677 (s), 1417 (m), 1341 (s), 730 (s), 693 (s).

4-Benzyl-3-((2-((*tert*-butyldimethylsilyl)oxy)phenyl)(3,5-di-*tert*-butyl-4-hydroxy-phenyl)methyl)-3,4-dihydroquinoxalin-2(1*H*)-one (3ai)

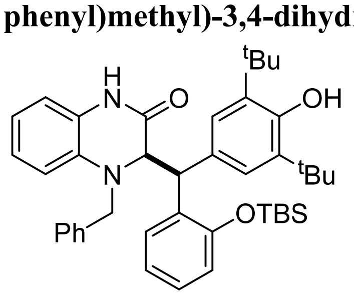

Using 4-benzyl-3,4-dihydroquinoxalin-2-one (**1a**, 38.4 mg, 0.15 mmol, 1.5 equiv.) and 2,6-di-*tert*-butyl-4-(2- ((*tert*-butyldimethylsilyl)oxy)benzylidene)cyclohexa-2,5-dien- 1- one (**2i**, 42.5 mg, 0.1 mmol, 1 equiv.), according to GP- 1, compound

3ai (64.3 mg, 0.097 mmol, 97% yield) was obtained as a mixture of diastereomers (2:1 dr) that were separated by column chromatography using hexane:EtOAc mixtures (from 9:1 to 8:2).

Characterization of **3ai'**: colorless oil; **^{1}H-NMR (300 MHz, CDCl$_3$)** δ 8.15 (s, 1H), 7.68 (dd, *J*=7.7, 1.8 Hz, 1H), 7.11 (m, 3H), 7.07–6.97 (m, 3H), 6.91 (td, *J*=7.5, 1.3 Hz, 1H), 6.83–6.75 (m, 3H), 6.71 (dd, *J*=8.0, 1.3 Hz, 1H), 6.63 (td, *J* = 7.6, 1.3 Hz, 1H), 6.53 (d, *J*=8.3 Hz, 1H), 6.49 (dd, *J*=7.7, 1.5 Hz, 1H), 4.82 (s, 1H), 4.63 (d, *J*=6.4 Hz, 1H), 4.46 (dd, *J*=6.4, 0.8 Hz, 1H), 4.34 (d, *J*=15.2 Hz, 1H), 3.92 (d, *J*=15.2 Hz, 1H), 1.18 (s, 18H), 0.75 (s, 9H), 0.06 (s, 3H), -0.13 (s, 3H). **^{13}C{^{1}H}-NMR (75 MHz, CDCl$_3$)** δ 166.0 (C), 153.1 (C), 152.1 (C), 136.9 (C), 134.7 (C), 133.8 (C), 133.0 (C), 130.1 (C), 129.5 (C), 128.4 (CH), 127.6 (CH), 127.22 (CH), 127.15 (CH), 126.9 (C), 126.0 (CH), 123.9 (CH), 120.9 (CH), 119.3 (CH), 118.3 (CH), 115.0 (CH), 114.2 (CH), 67.3 (CH), 53.3 (CH$_2$), 44.7 (CH), 34.2 (C), 30.2 (CH$_3$), 26.0 (CH$_3$), 18.3 (C), -4.0 (CH$_3$), -4.2 (CH$_3$); **HRMS (ESI/Q-TOF)** *m/z* [M + H]$^+$ calcd for $C_{42}H_{55}N_2O_3Si^+$ 663.3976, found 663.3979. **FTIR (neat)** cm^{-1} : 3630 (w), 2955 (m), 1680 (s), 1610 (m), 1110 (s), 697 (s).

Characterization of **3ai''**: colorless oil; **^{1}H-NMR (300 MHz, CDCl$_3$)** δ 7.81 (s, 1H), 7.69 (dd, *J* =7.4, 2.1 Hz, 1H), 7.25–7.16 (m, 3H), 7.08 – 6.92 (m, 7H), 6.80 (td, *J*=7.5, 1.2 Hz, 1H), 6.77–6.63 (m, 2H), 6.53 (d, *J*=8.2 Hz, 1H), 5.13 (s, 1H), 4.58 (d, *J*=11.2 Hz, 1H), 4.49 (dd, *J*=11.2, 1.0 Hz, 1H), 3.92 (d, *J*=15.4 Hz, 1H), 3.37 (d, *J*= 5.4 Hz, 1H), 1.40 (s, 18H), 0.80 (s, 9H), 0.08 (s, 3H), 0.06 (s, 3H). **^{13}C{^{1}H}-NMR (75 MHz, CDCl$_3$)** δ 165.2 (C), 153.3 (C), 152.9 (C), 137.3 (C), 135.8 (C), 133.5 (C), 131.2 (C), 130.2 (C), 129.3 (CH), 128.5 (CH), 127.4 (CH), 127.3 (CH), 126.7 (C), 125.9 (CH), 123.8 (CH), 120.5 (CH), 119.0 (CH), 118.4 (CH), 115.5 (CH), 115.0 (CH), 66.0 (CH), 54.0 (CH$_2$), 34.4 (C), 30.3 (CH$_3$), 25.9 (CH$_3$), 18.2 (C), -3.6 (CH$_3$), -4.5 (CH$_3$); **HRMS (ESI/Q-TOF)** *m/z* [M + H]$^+$ calcd for $C_{42}H_{55}N_2O_3Si^+$ 663.3976, found 663.3977. **FTIR (neat)** cm^{-1} : 3627 (w), 2955 (m), 1684 (s), 1610 (m), 1114 (s), 693 (s).

4-((1-Benzyl-3-oxo-1,2,3,4-tetrahydroquinoxalin-2-yl)(3,5-di-*tert*-butyl-4-hydroxyphenyl)methyl)phenyl acetate (3aj)

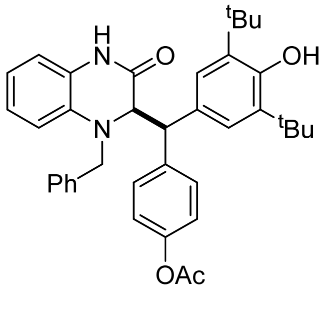

Using 4-benzyl-3,4-dihydroquinoxalin-2-one (**1a**, 38.4 mg, 0.15 mmol, 1.5 equiv.) and 4-((3,5-di-*tert*-butyl-4-oxocyclohexa-2,5-dien-1-ylidene)methyl)phenyl acetate (**2j**, 35.2 mg, 0.1 mmol, 1 equiv.), according to GP- 1, compound **3aj** (50.9 mg, 0.086 mmol, 86% yield) was obtained as a mixture of diastereomers (1:1 dr) that

were separated by column chromatography using hexane:EtOAc mixtures (from 9:1 to 8:2).

Characterization of **3aj'**: yellow solid, m.p.: 195-200 °C; **^{1}H NMR (500 MHz, $CDCl_3$)** δ 8.63 (s, 1H), 7.37–7.17 (m, 5H), 7.08 (d, *J* =7.1 Hz, 2H), 7.01 (d, *J*=8.4 Hz, 2H), 6.97 (s, 2H), 6.92 (t, *J*=7.2 Hz, 1H), 6.75 (t, *J* =7.4 Hz, 1H), 6.65–6.57 (m, 2H), 5.01 (s, 1H), 4.56 (d, *J*=7.8 Hz, 1H), 4.28 (d, *J*=15.4 Hz, 1H), 4.10 (d, *J*=7.8 Hz, 1H), 3.81 (d, *J*=15.3 Hz, 1H), 2.31 (s, 3H), 1.33 (s, 18H); **$^{13}C\{^1H\}$ NMR (126 MHz, $CDCl_3$)** δ 169.4 (C), 165.7 (C), 152.5 (C), 149.4 (C), 140.0 (C), 136.8 (C), 135.2 (C), 133.5 (C), 129.7 (CH), 129.5 (C), 128.5 (CH), 127.5 (CH), 127.3 (CH), 126.9 (C), 125.6 (CH), 123.8 (CH), 121.5 (CH), 119.3 (CH), 115.2 (CH), 114.5 (CH), 66.5 (CH), 54.1 (CH_2), 52.3 (CH), 34.2 (C), 30.2 (CH_3), 21.2 (CH_3); **HRMS (ESI/Q-TOF)** *m/z* $[M + H]^+$ calcd for $C_{38}H_{43}N_2O_4^+$ 591.3217, found 591.3222. **FTIR (neat)** cm^{-1} : 3619 (w), 2952 (m), 1751 (m), 1684 (s), 1505 (m), 1192 (s), 909 (m), 727 (s).

Characterization of **3aj''**: yellow oil; **^{1}H NMR (500 MHz, $CDCl_3$)** δ 8.48 (s, 1H), 7.35 (d, *J*=8.5 Hz, 2H), 7.24–7.11 (m, 3H), 7.01–6.92 (m, 7H), 6.83 (t, *J*=7.5 Hz, 1H), 6.69 (d, *J*=7.6 Hz, 1H), 6.54 (d, *J*=8.0 Hz, 1H), 5.19 (s, 1H), 4.48 (d, *J*=10.3 Hz, 1H), 4.02 (d, *J*=15.3 Hz, 1H), 3.98 (d, *J*=10.3 Hz, 1H), 3.49 (d, *J*=15.2 Hz, 1H), 2.22 (s, 3H), 1.43 (s, 18H); **$^{13}C\{^1H\}$ NMR (126 MHz, $CDCl_3$)** δ 169.1 (C), 165.6 (C), 153.1 (C), 149.4 (C), 137.6 (C), 137.0 (C), 136.2 (C), 133.4 (C), 131.4 (C), 129.7 (CH), 128.5 (CH), 127.4 (CH), 127.4 (CH), 126.9 (C), 125.4 (CH), 123.8 (CH), 121.1 (CH), 119.3 (CH), 115.6 (CH), 114.9 (CH), 66.1 (CH), 54.0 (CH_2), 51.4 (CH), 34.4 (C), 30.3 (CH_3), 21.1 (CH_3); **HRMS (ESI/Q-TOF)** *m/z* $[M + H]^+$ calcd for $C_{38}H_{43}N_2O_4^+$ 591.3217, found 591.3219. **FTIR (neat)** cm^{-1} : 3630 (w), 2955 (m), 1759 (m), 1684 (s), 1505 (m), 1192 (s), 730 (s).

4-Benzyl-3-(1-(3,5-di-*tert*-butyl-4-hydroxyphenyl)ethyl)-3,4-dihydroquinoxalin-2(1*H*)-one (3ak)

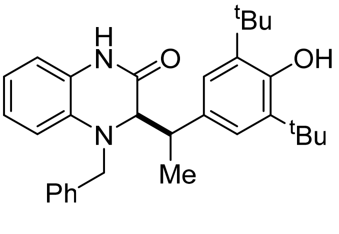

Using 4-benzyl-3,4-dihydroquinoxalin-2-one (**1a**, 38.4 mg, 0.15 mmol, 1.5 equiv.) and 2,6-di-*tert*-butyl-4-ethylidenecyclohexa-2,5-dien-1-one (**2k**, 23.2 mg, 0.1 mmol, 1 equiv.), according to GP- 1, compound **3ak** (46.8 mg, 0.099 mmol, 99% yield, colorless oil) was obtained as a mixture of diastereomers (1.3:1 dr) that were separated by column chromatography using hexane:EtOAc mixtures (from 9:1 to 8:2). Major and minor diastereoisomers are marked with one or two asterisks, respectively.

^{1}H NMR (500 MHz, CDCl$_3$) δ 9.53 (s, 1H*), 9.40 (s, 1H**), 7.35–7.14 (m, 9H), 6.99–6.93 (m, 4H), 6.90 (dd, *J*=7.7, 1.4 Hz, 1H*), 6.87 (s, 2H*), 6.84–6.74 (m, 2H), 6.55 (d, *J*=7.6 Hz, 1H*), 6.53–6.50 (m, 2H**), 6.47 (dd, *J* = 7.6, 1.5 Hz, 1H**), 5.21 (s, 1H*), 5.00 (s, 1H**), 4.67 (d, *J*=15.2 Hz, 1H**), 4.13–4.06 (m, 2H**), 3.97 (d, *J*=15.2 Hz, 1H*), 3.78 (d, *J*=9.8 Hz, 1H*) 3.33 (d, *J*=15.2 Hz, 1H*), 3.28 (dq, *J*=10.1, 7.1 Hz, 1H**), 2.90 (dq, *J*=10.1, 7.1 Hz, 1H*), 1.46 (s, 18H*), 1.42–1.37 (m, 21H**), 1.35 (d, *J*=7.1 Hz, 3H*); ^{13}C NMR (126 MHz, CDCl$_3$) δ 167.5 (C), 167.5 (C), 152.9 (C), 152.5 (C), 137.1 (C), 136.9 (C), 136.1 (C), 135.2 (C), 134.6 (C), 134.0 (C), 133.3 (C), 131.5 (C), 128.7 (CH), 128.4 (CH), 127.5 (CH), 127.4 (2CH), 127.3 (CH), 126.8 (C), 125.8 (C), 124.9 (CH), 124.6 (CH), 123.8 (2CH), 118.8 (CH), 117.9 (CH), 115.6 (CH), 114.8 (CH), 114.4 (CH), 112.2 (CH), 68.1 (CH), 67.6 (CH), 53.8 (CH$_2$), 53.2 (CH$_2$), 42.5 (CH), 39.5 (CH), 34.4 (C), 34.1 (C), 30.4 (CH$_3$), 30.3 (CH$_3$), 17.7 (CH$_3$), 17.7 (CH$_3$); **HRMS (ESI/Q-TOF)** *m/z* [M + H]$^+$ calcd for $C_{31}H_{39}N_2O_2^+$ 471.3006, found 471.3011. **FTIR (neat)** cm^{-1} : 3630 (w), 2952 (m), 1669 (s), 1610 (s), 745 (s), 697 (s).

2-((1-Benzyl-3-oxo-1,2,3,4-tetrahydroquinoxalin-2-yl)(3,5-di-*tert*-butyl-4-hydroxy-phenyl)methyl)phenyl 2-(1-(4-chlorobenzoyl)-5-methoxy-2-methyl-1*H*-indol-3-yl) acetate (3al)

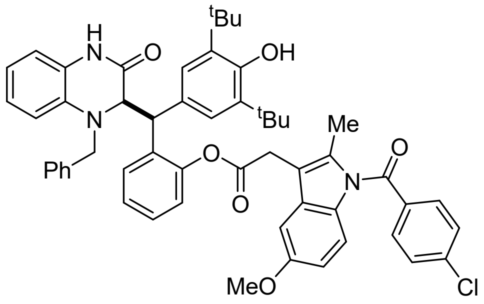

Using 4-benzyl-3,4-dihydroquinoxalin-2-one (**1a**, 38.4 mg, 0.15 mmol, 1.5 equiv.) and 2-((3,5-di-*tert*-butyl-4- oxocyclohexa- 2,5-dien-1-ylidene) methyl)phenyl 2-(1-(4-chlorobenzoyl)-5-methoxy-2-methyl-1*H*-indol-3-yl) acetate (**2l**, 64.9 mg, 0.1 mmol, 1 equiv.), according to GP-1, compound **3al** (70.2 mg, 0.079 mmol, 79% yield) was obtained as a mixture of diastereomers (1:1 dr) that were separated by column chromatography using hexane:EtOAc mixtures (from 9:1 to 8:2).

Characterization of **3al'**: yellow oil; **^{1}H-NMR (300 MHz, CDCl$_3$)** *δ* 8.14 (s, 1H), 7.72–7.67 (m, 1H), 7.64 (d, *J*=8.7 Hz, 1H), 7.45 (d, *J*=8.7 Hz, 1H), 7.24–7.16 (m, 6H), 7.06–7.01 (m, 2H), 7.00–6.92 (m, 6H), 6.86 (dd, *J*=9.0, 0.5 Hz, 1H), 6.76 (td, *J*=7.5, 1.2 Hz, 1H), 6.71 (d, *J*=8.8 Hz, 1H), 6.69–6.61 (m, 2H), 5.00 (bs, 1H), 4.68 (dd, *J*=7.1, 0.8 Hz, 1H), 4.45 (d, *J*=7.1 Hz, 1H), 4.36 (d, *J*=15.4 Hz, 1H), 3.89 (d, *J*= 5.5 Hz, 1H), 3.75 (s, 3H), 3.55 (d, *J*=16.3 Hz, 1H), 3.39 (d, *J*=16.2 Hz, 1H), 2.33 (s, 3H), 1.27 (s, 18H).

^{13}C{^{1}H}-NMR (75 MHz, $CDCl_3$) δ 169.0 (C), 168.2 (C), 165.2 (C), 156.1 (C), 152.5 (C), 148.4 (C), 139.3 (C), 136.7 (C), 136.2 (C), 135.2 (C), 134.1 (C), 133.8 (C), 133.7 (C) 131.2 (CH), 130.8 (C), 130.5 (C), 129.8 (CH), 129.1 (CH), 128.5 (CH), 127.51 (CH), 127.49 (CH), 127.3 (CH), 126.8 (C), 126.1 (CH), 125.7 (CH), 124.0 (CH), 122.6 (CH), 119.3 (C), 115.1 (CH), 115.02 (CH), 114.97 (CH), 111.9 (C), 111.5 (CH), 101.5 (CH), 66.3 (CH), 55.7 (CH), 53.8 (CH_2), 45.3 (CH_3), 34.2 (C), 30.1 (CH_3), 29.7 (CH_2), 13.5 (CH_3); **HRMS (ESI/Q-TOF)** *m/z* $[M + H]^+$ calcd for $C_{55}H_{55}ClN_3O_6^+$ 888.3774, found 888.3778. **FTIR (neat)** cm^{-1} : 3630 (w), 2955 (m), 1759 (m), 1677 (s), 1591 (m), 1312 (s), 1118 (s), 749 (s).

Characterization of **3al''**: yellow oil; **^{1}H-NMR (300 MHz, $CDCl_3$)** δ 8.21 (s, 1H), 7.71–7.67 (m, 1H), 7.64 (d, *J*=8.6 Hz, 2H), 7.46 (d, *J*=8.5 Hz, 2H), 7.24–7.16 (m, 4H), 7.12 (td, *J*=7.8, 1.8 Hz, 1H), 7.06–6.95 (m, 6H), 6.91 (d, *J* = 2.5 Hz, 1H), 6.90–6.79 (m, 4H), 6.73–6.59 (m, 2H), 4.60 (dd, *J* = 10.5, 0.9 Hz, 1H), 4.25 (d, *J*=10.5 Hz, 1H), 4.10 (d, *J*=14.9 Hz, 1H), 3.78 (s, 3H), 3.60–3.36 (m, 3H), 2.28 (s, 3H), 1.42 (s, 18H).**^{13}C{^{1}H}-NMR (75 MHz, $CDCl_3$)** δ 168.6 (C), 168.2 (C), 164.9 (C), 156.0 (C), 153.2 (C), 148.6 (C), 139.3 (C), 136.8 (C), 136.2 (C), 136.1 (C), 133.8 (C), 133.5 (C), 131.2 (C), 131.2 (CH), 130.8 (C), 130.7 (C), 130.5 (C), 129.7 (C), 129.1 (CH), 128.6 (CH), 127.69 (CH), 127.65 (CH), 127.5 (CH), 127.0 (CH), 125.7 (CH), 125.5 (CH), 123.9 (CH), 122.4 (CH), 119.5 (CH), 115.5 (CH), 115.2 (CH), 114.9 (CH), 111.9 (C), 111.5 (CH), 101.5 (CH), 64.7 (CH), 55.7 (CH), 54.1 (CH_2), 34.4 (C), 30.3 (CH_3), 29.9 (CH_2), 29.4 (CH_3), 13.4 (CH_3); **HRMS (ESI/Q-TOF)** *m/z* $[M + H]^+$ calcd for $C_{55}H_{55}ClN_3O_6^+$ 888.3774, found 888.3783. **FTIR (neat)** cm^{-1} : 3630 (w), 2952 (m), 1759 (m), 1677 (s), 1591 (m), 1356 (m), 1312 (s), 1118 (s), 749 (s).

Luminescence Quenching Experiments

DCM over 3Å MS was degassed by sonication and N_2 bubbling simultaneously. The measurement solutions were prepared from stock solutions of **1** (32 mM), and [Mes-Acr-Me][BF_4] (0.4 mM) in anhydrous and degassed DCM. All the emission spectra were obtained using a Jasco FP-750 Spectrofluorometer selecting 450 nm as the excitation wavelength.

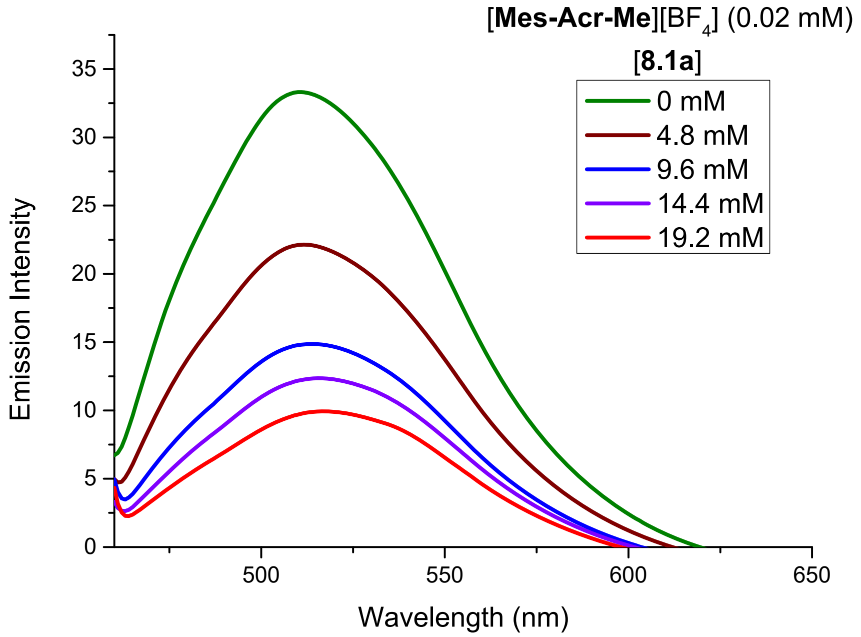

Figure 1: Emission spectra of different DCM solutions containing 0.02 mM of [Mes-Acr-Me][BF_4] and varying amounts of 3,4-dihydroquinoxalin-2-one **1a**.

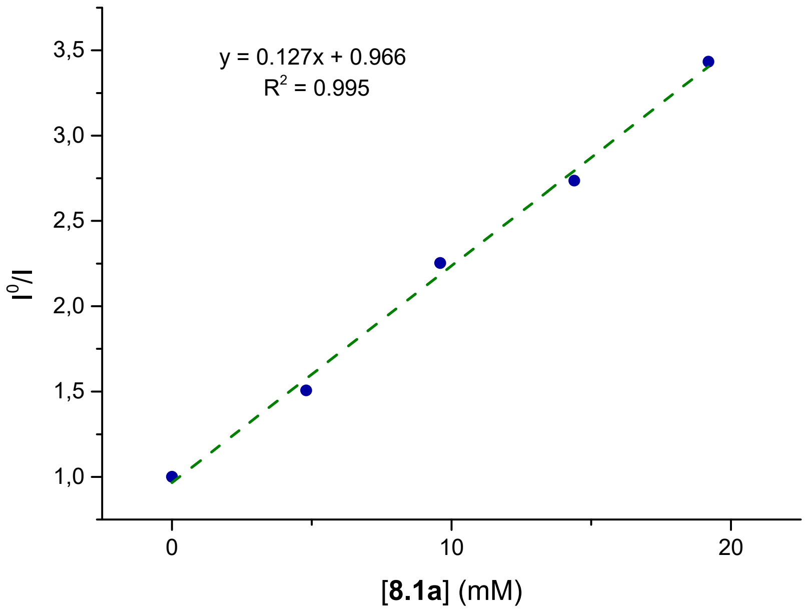

Figure 2: Stern-Volmer plot of I^0/I vs [**1a**]. Determination of K_{SV} through linear regression.

Reactive implications of captodative radical

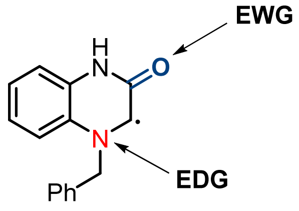

Figure 3: Schematic representation of the captodative radical that is generated via photoredox catalysis.

It is known that a radical is stabilized by electron-withdrawing groups (EWG) and electron-donating groups (EDG). But, when a radical centre has both a EWG and a EDG attached (this radical is called captodative) it is even more stable. A rational explanation for this captodative effect comes from Molecular Orbital Theory (MOT). The following scheme describes the orbital interactions in the formation of a sigma C-N bond between a α-carbonyl radical (stabilized radical due to the EWG) and a nitrogen (EDG).[8]

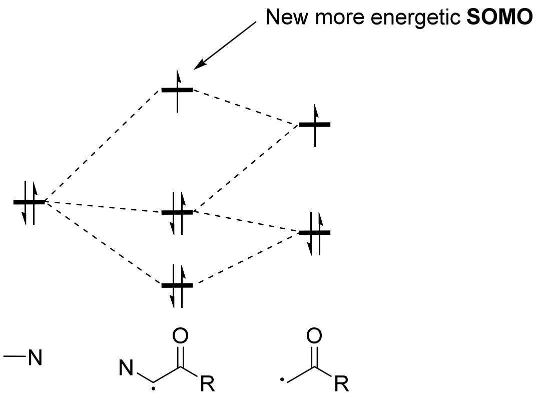

Figure 4 Molecular Orbitals Diagram for the formation of a sigma C-N bond between a nitrogen and a α-carbonyl radical.

The interaction between N and a α-EWG-radical provides a set of new MOs (Figure 4, centre). As can be noted, there is a rise in energy of the SOMO, increasing the nucleophilicity of the radical while achieving an overall stabilization.

Additionally, within the resonance model, radicals featuring both EDG and EWG have additional charge-separate resonance structures (Figure 5) in comparison with radicals bearing either multiple EDG or EWG. This feature also supports its greater stability.

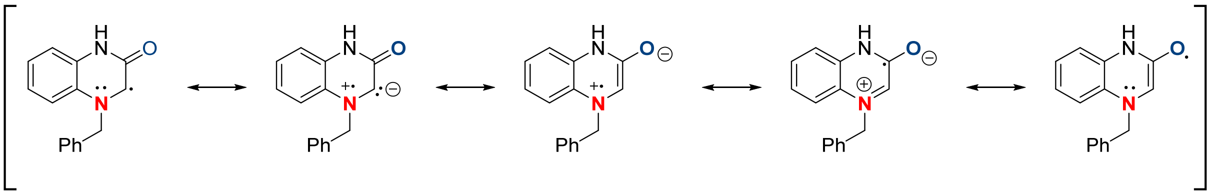

Figure 5: Resonance structures for the radical generated under photoredox conditions

Determination of the Quantum Yield of the photochemical reaction:

With the determined photon flux of the HP Single Blue LED ($4.18 \cdot 10^{-7}$ einsteins/s), the same equation must be employed for the determination of the quantum yield of the photochemical 1,6-addition. For that, the moles of product **3aa** for a given time must be determined.

Following General Procedure 1 (GP-1), the reaction between **1a** and **2a** in the presence of [Mes-Acr-Me][BF_4] was carried out. After 10 minutes, a 0.2 mL aliquot was taken out and filtered through silica and eluted with EtOAc. The moles of product **3aa** were determined by ^{1}H-NMR using 4-methoxyacetophenone as internal standard. This process was repeated taking another aliquot after 20 of irradiation. The kinetic profile of the reaction is shown in Figure .

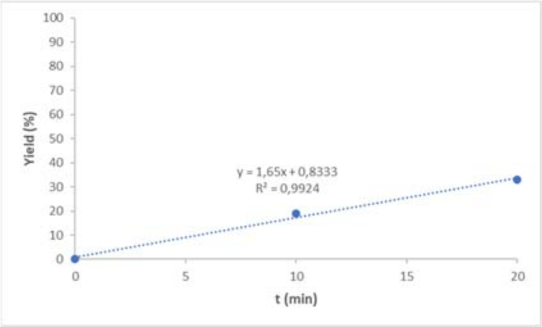

Figure 6: Kinetic profile for the photochemical reaction after 10 and 20 minutes of irradiation.

Then, the following equation was used to determine the quantum yield:

$$Quantum\ yield\ \left(\frac{mol}{einstein}\right) = \frac{\boldsymbol{3aa}\ (mol)}{Photon\ flux\ \left(\frac{einstein}{s}\right) \cdot time\ (s) \cdot F}$$

Where:

- **3aa** (mol) is the amount of **3aa** that has been formed during the irradiation time.
- Time (s) is the irradiation time (in seconds).
- F is the fraction of light absorbed, and it is calculated with $F = 1 - 10^{-A(455\ nm)} = 0.8958$, where A(455 nm) is the absorbance of the reaction at 455 nm (0.982).

Run	Irradiation Time (min)	Quantum Yield
1	10	0.0428
2	20	0.0367
		**Avg.**: 0.040±0.004

References

[1]Bonuga, Y. R.; Ravinder-Nathb, A.; Balramc, B.; Ram, B. *Der Pharma Chemica* **2013**, *5*, 296–300.

[2]Carrër, A.; Brion, J.-D.; Messaoudi, S.; Alami, M. *Org. Lett.* **2013**, *15*, 5606–5609.

[3]Li, D.; Ollevier, T. Eur. *J. Org. Chem*. **2018**, *2019*, 1273–1280.

[4]Qiao, J. X. et al. *J. Med. Chem*. **2013**, *56*, 9275–9295.

[5]Weber Eckard; Keana, J. F. W. US Patent, US5514680, **1996**.

[6]Torán, R.; Vila, C.; Sanz-Marco, A.; Muñoz, M. C.; Pedro, J. R.; Blay, G. *Eur. J. Org. Chem*. **2020**, *5*, 627-630.

[6]Rostoll-Berenguer, J.; Martín-López, M.; Blay, G. Pedro, J. R.; Vila, C.; *J. Org. Chem*. **2022**, 87, 9343-9356.

[8]Fleming, I; Molecular Orbitals and Organic Chemical Reactions, 2009, John Wiley & Sons, pages 67-69

NMR Spectra

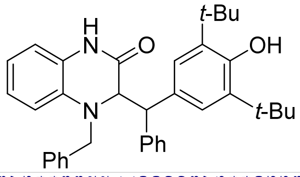

3aa'
^{1}H NMR ($CDCl_3$, 300 MHz)
^{13}C {^{1}H}-NMR ($CDCl_3$, 75 MHz)

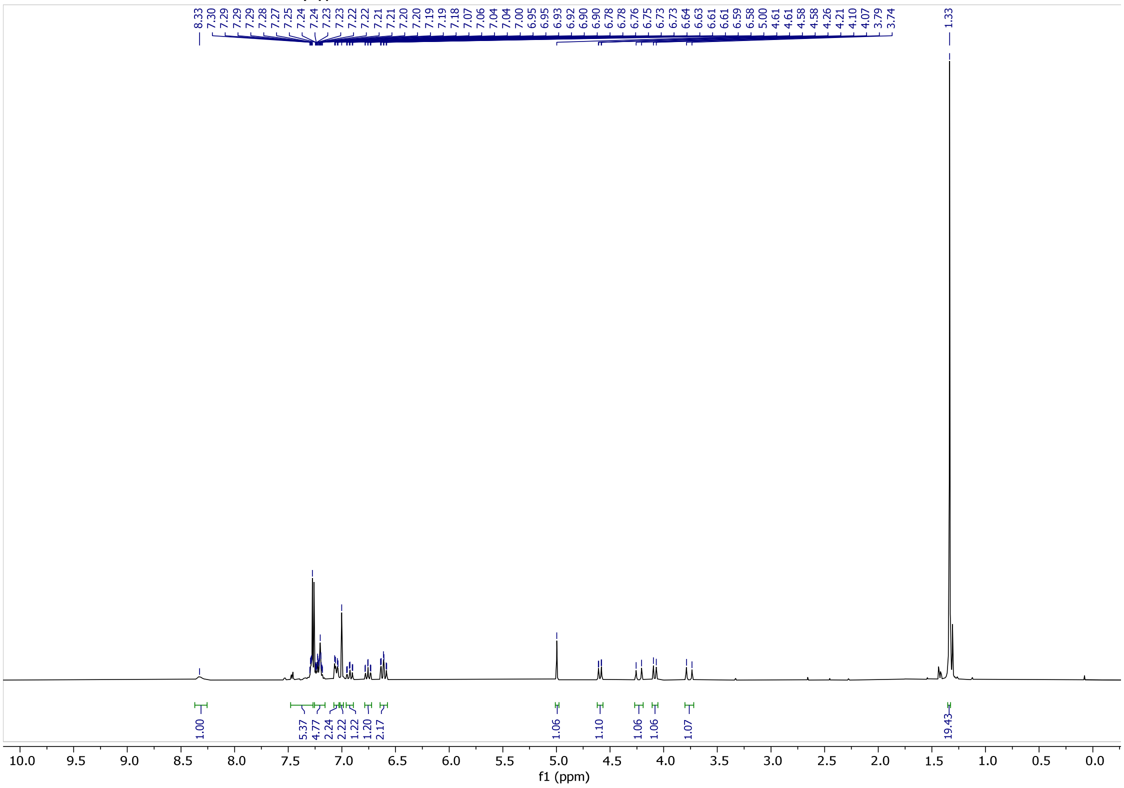

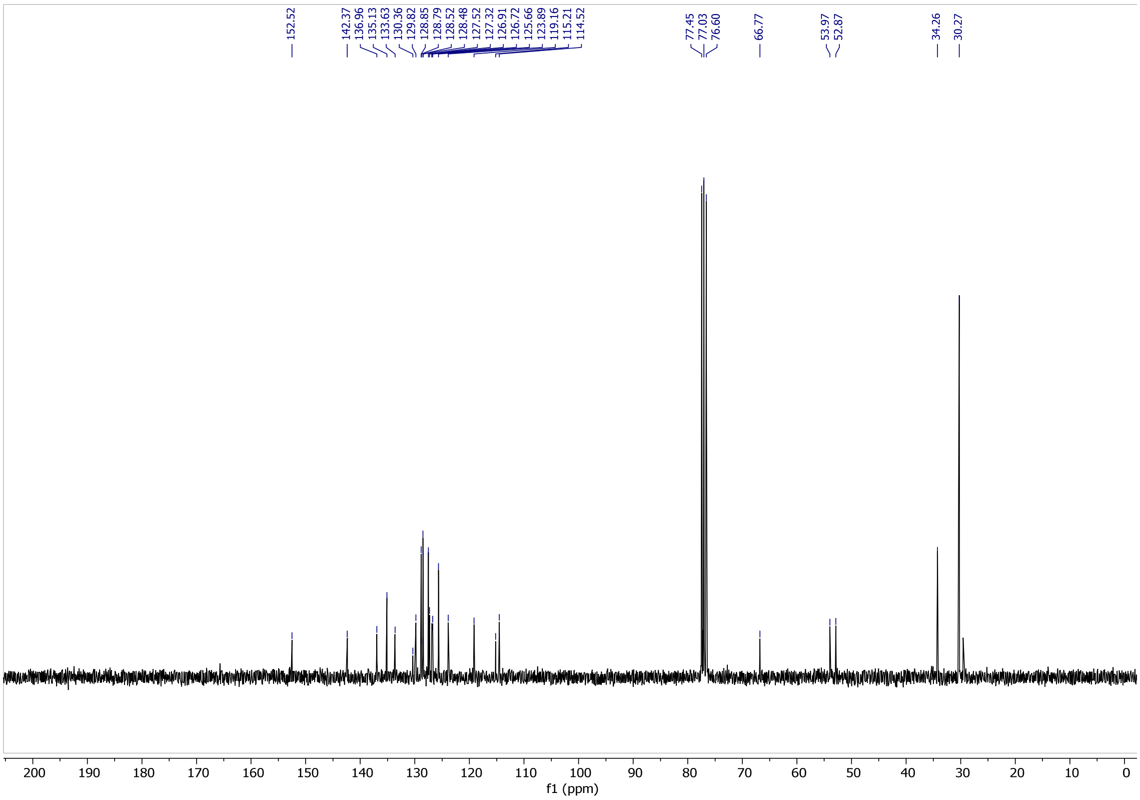

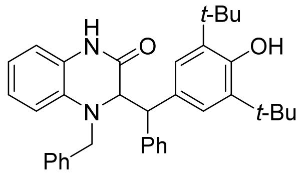

3aa''

^{1}H NMR ($CDCl_3$, 300 MHz)

^{13}C {^{1}H}-NMR ($CDCl_3$, 75 MHz)

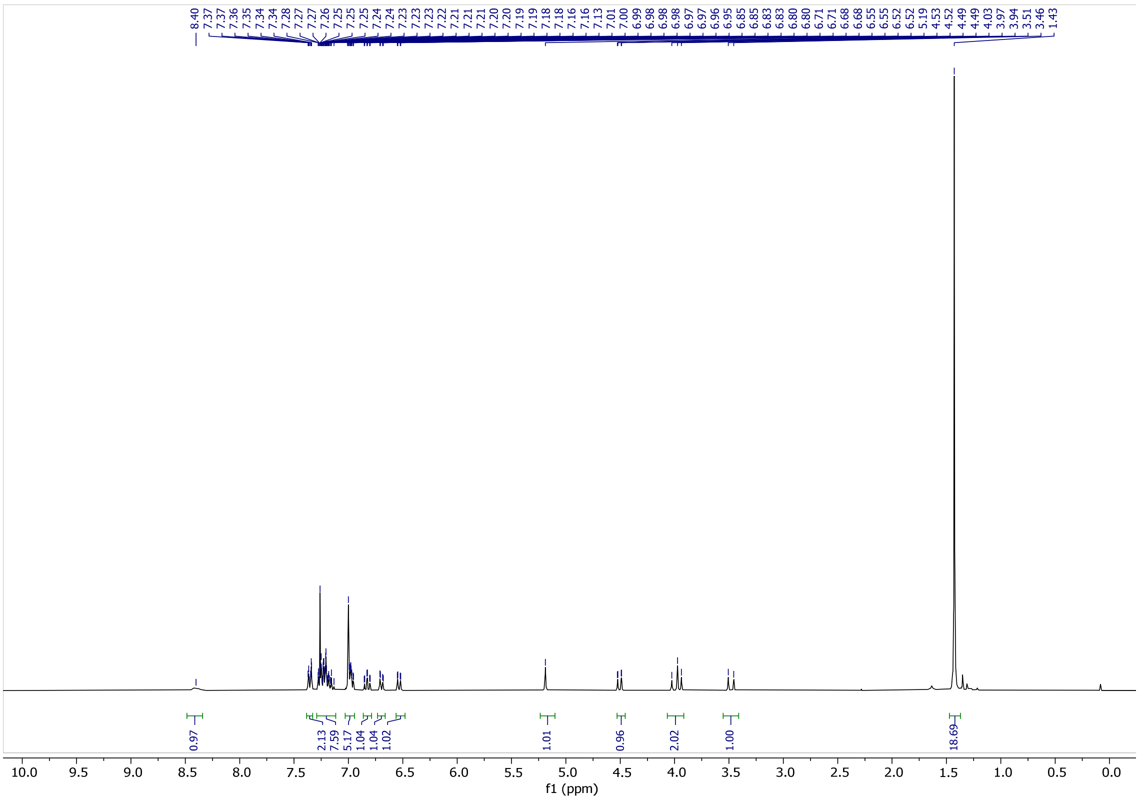

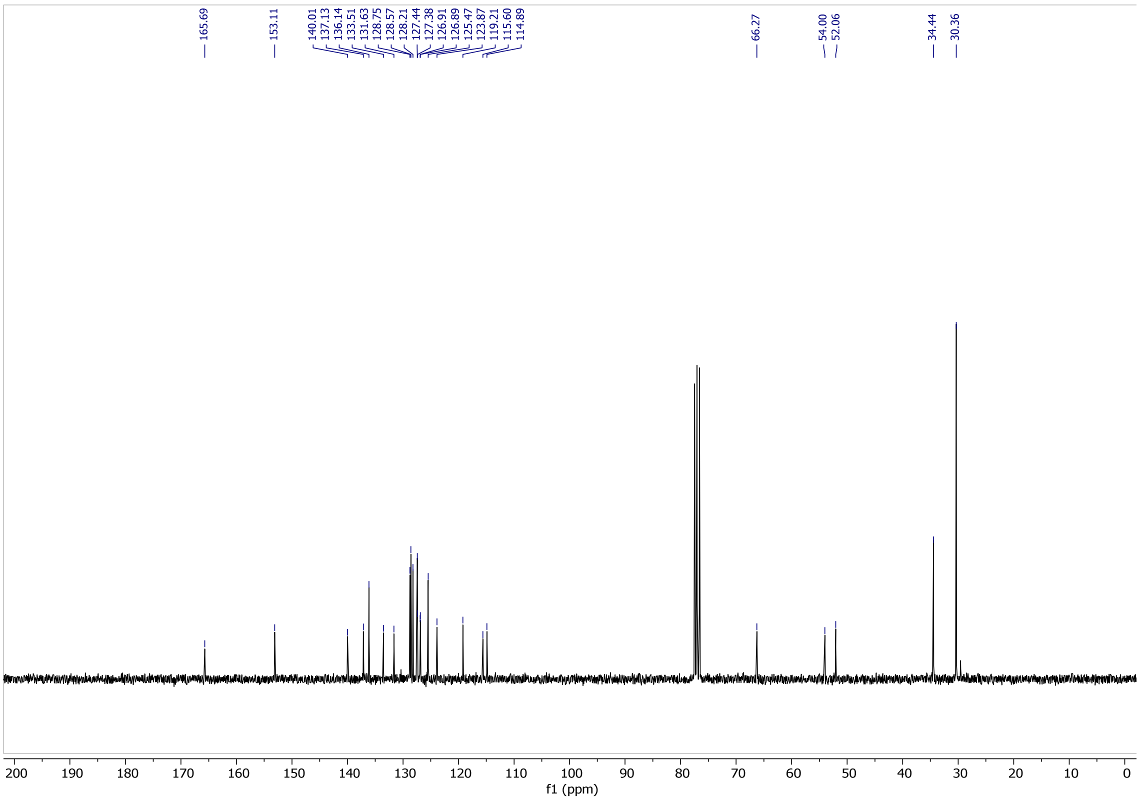

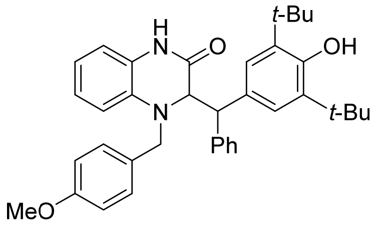

3ba'
^{1}H NMR (CDCl$_3$, 300 MHz)
^{13}C {^{1}H}-NMR (CDCl$_3$, 75 MHz)

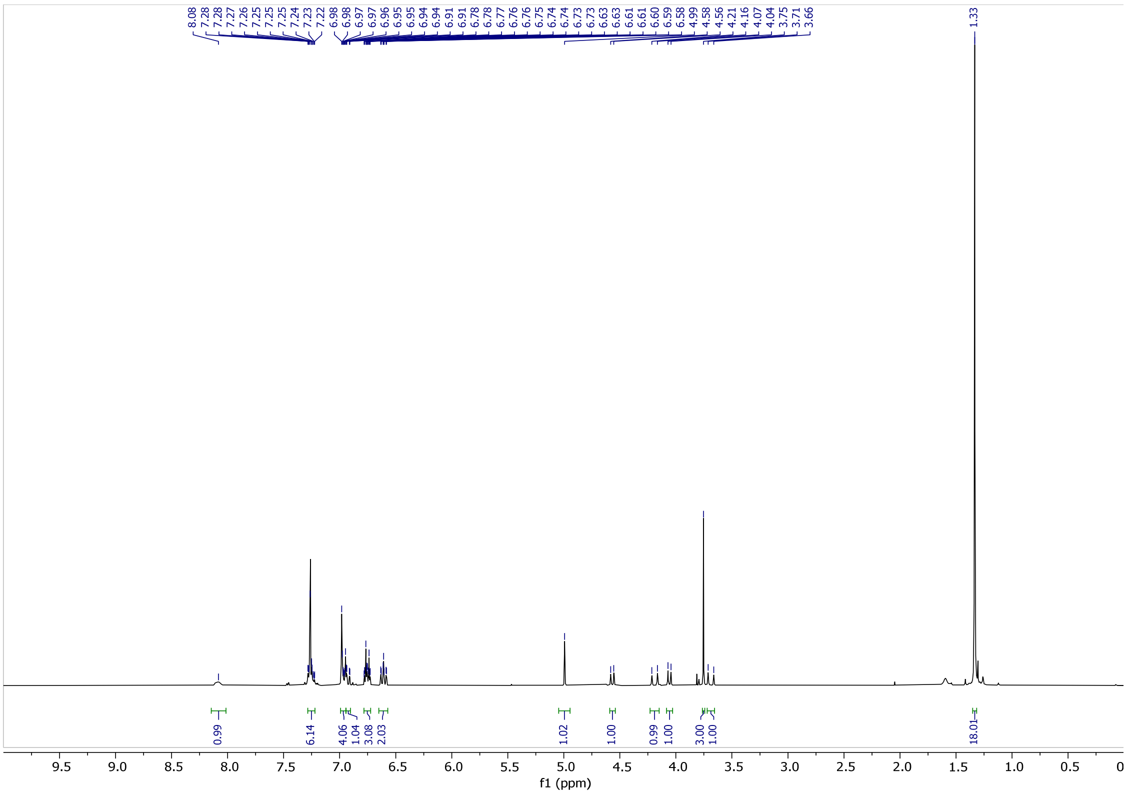

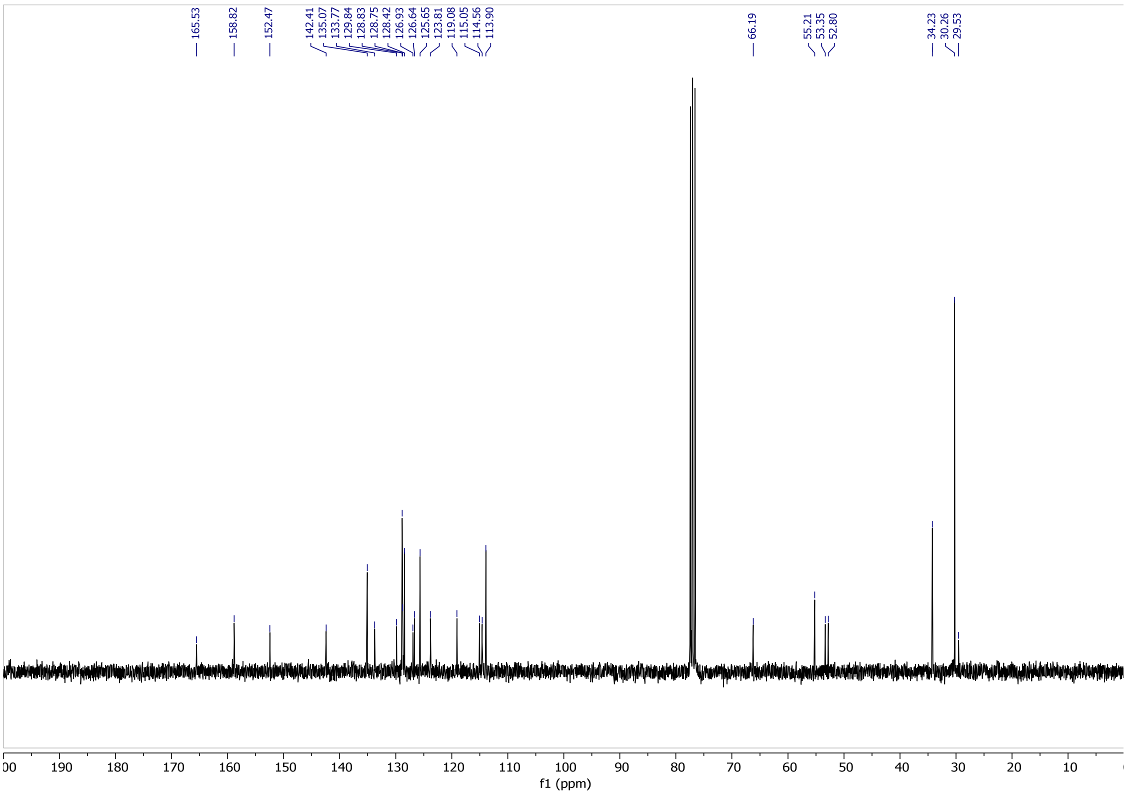

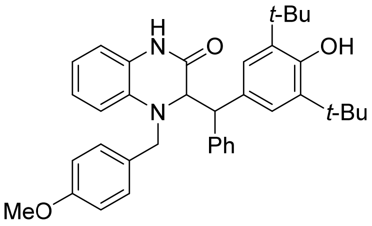

3ba''
^{1}H NMR ($CDCl_3$, 300 MHz)
^{13}C {^{1}H}-NMR ($CDCl_3$, 75 MHz)

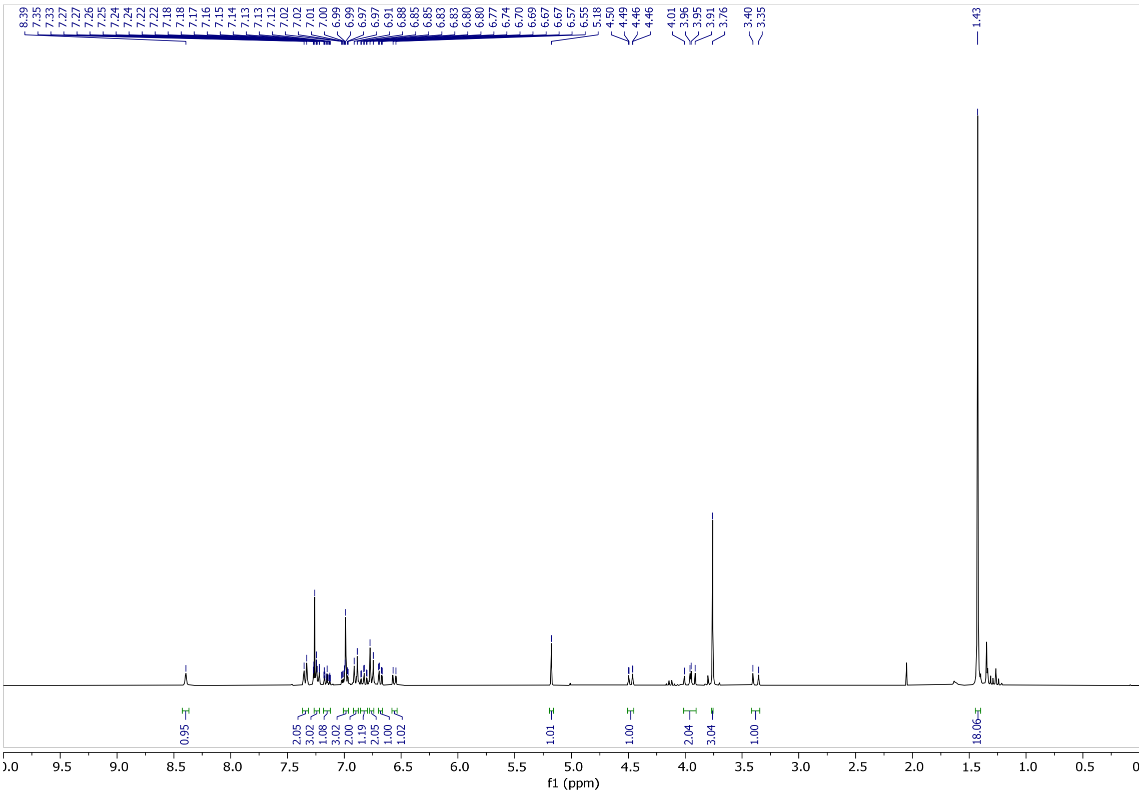

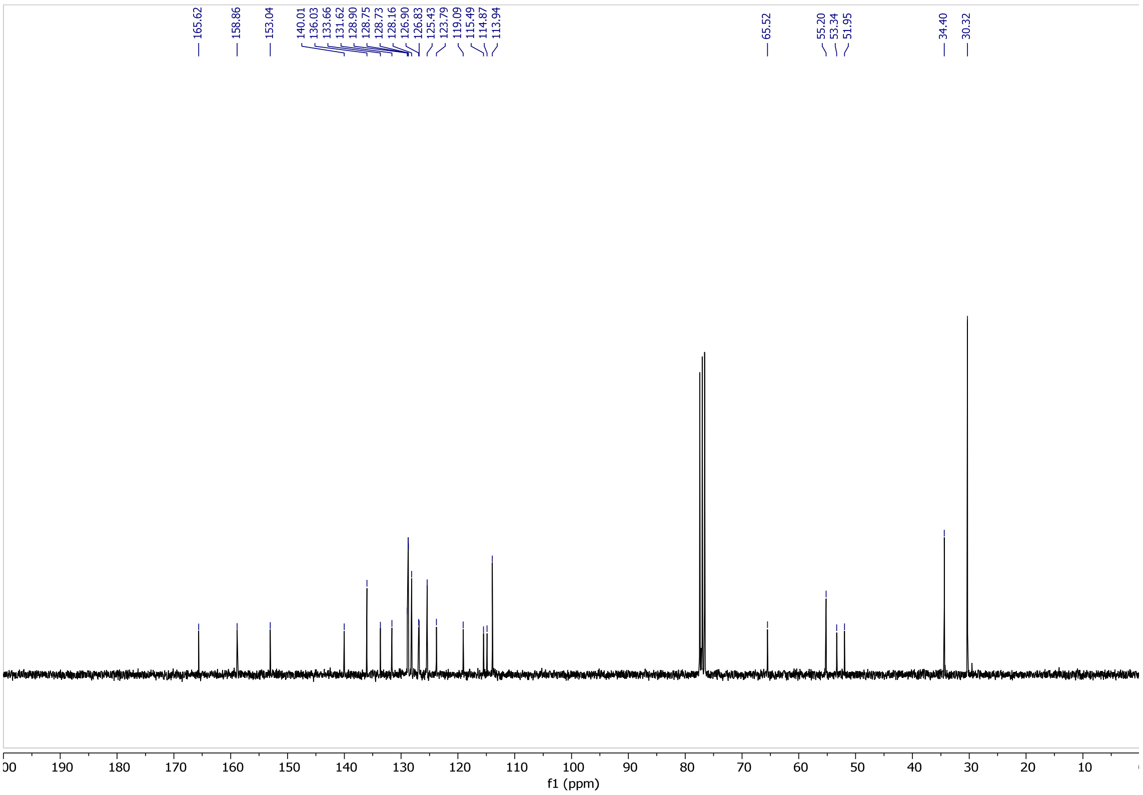

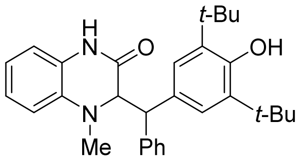

3ca'

^{1}H NMR ($CDCl_3$, 500 MHz)

^{13}C {^{1}H}-NMR ($CDCl_3$, 126 MHz)

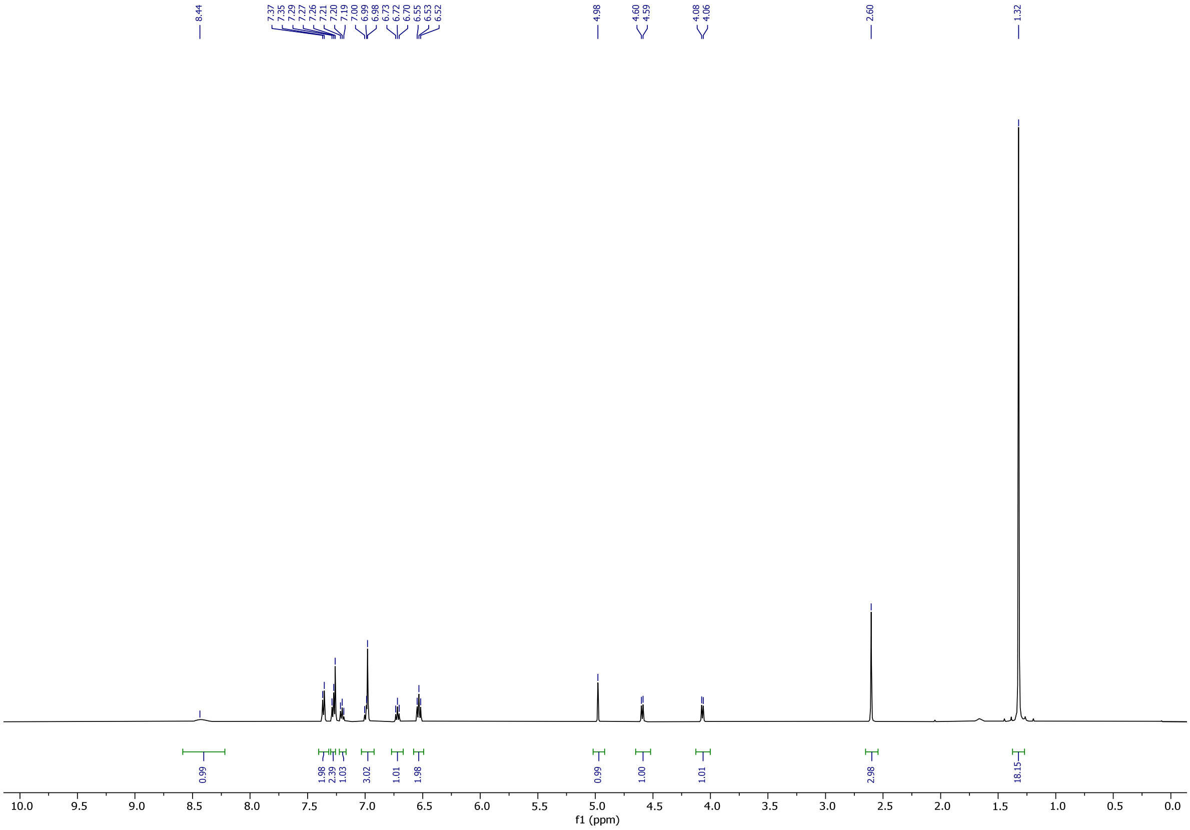

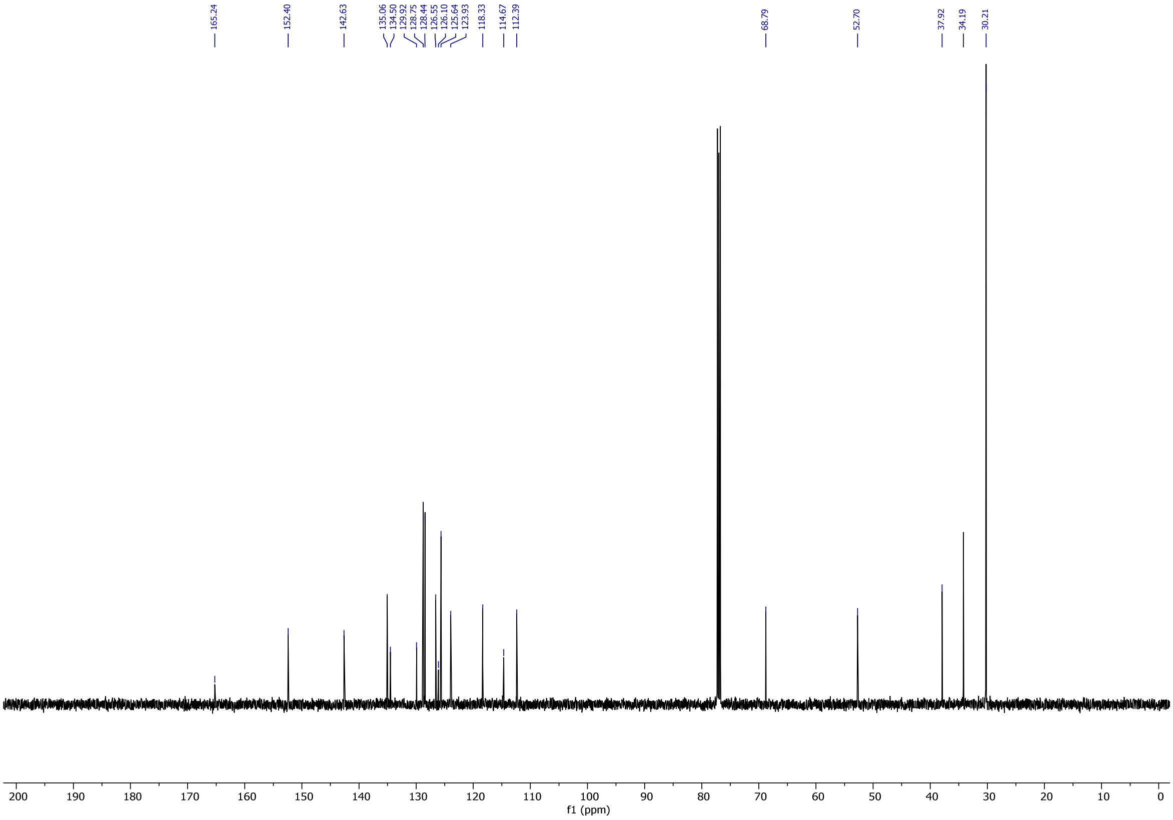

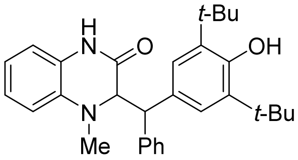

3ca''
^{1}H NMR ($CDCl_3$, 500 MHz)
^{13}C {^{1}H}-NMR ($CDCl_3$, 126 MHz)

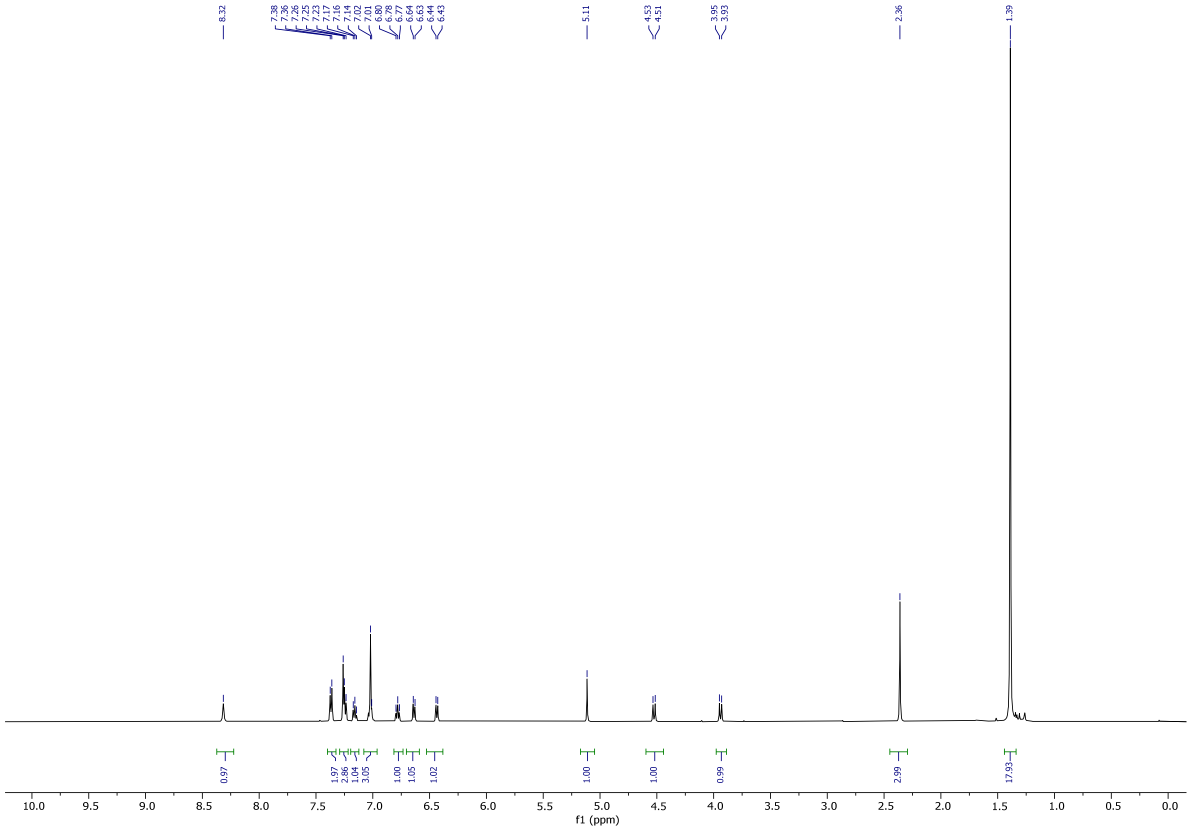

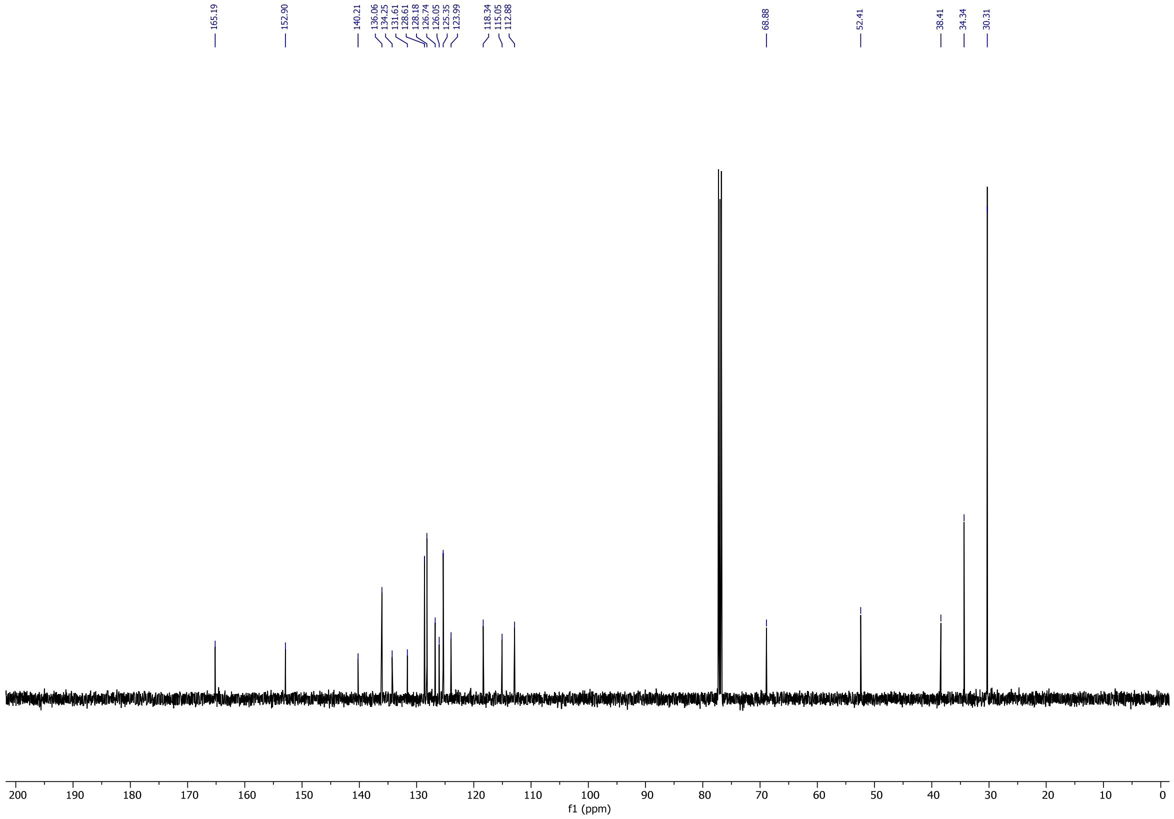

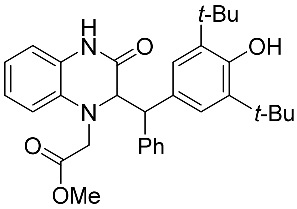

3da
^{1}H NMR ($CDCl_3$, 300 MHz)
^{13}C {^{1}H}-NMR ($CDCl_3$, 75 MHz)

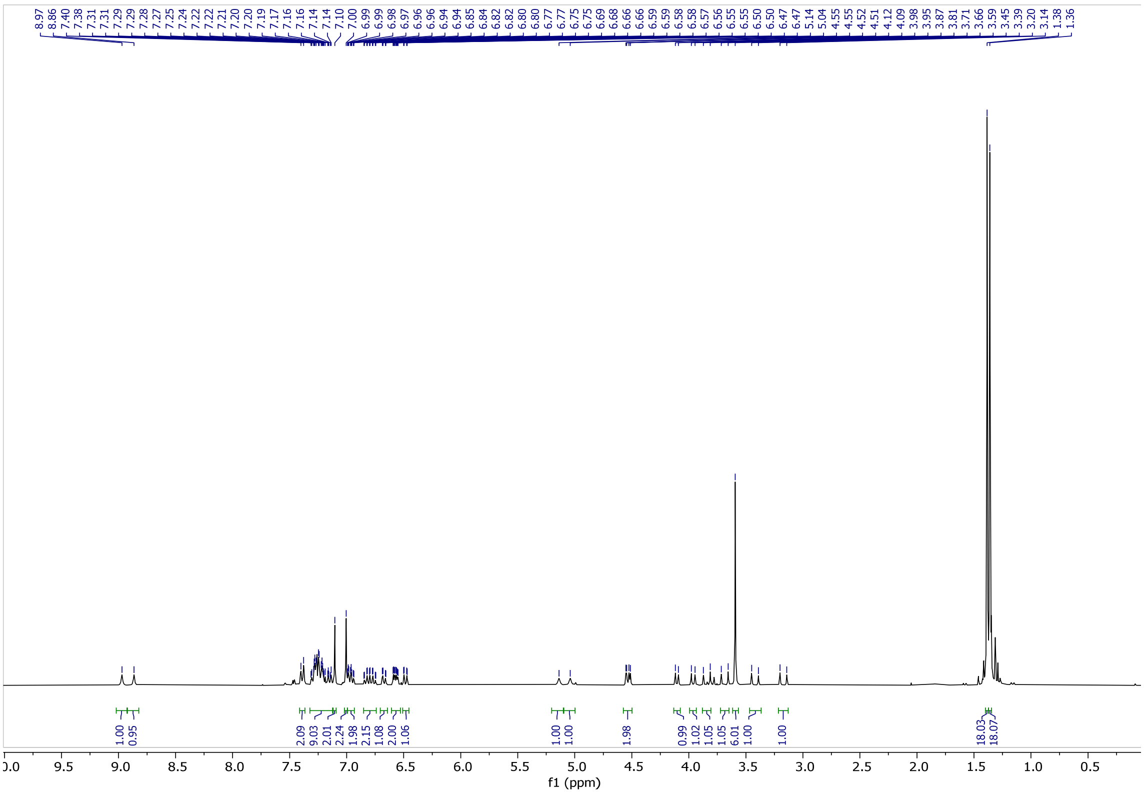

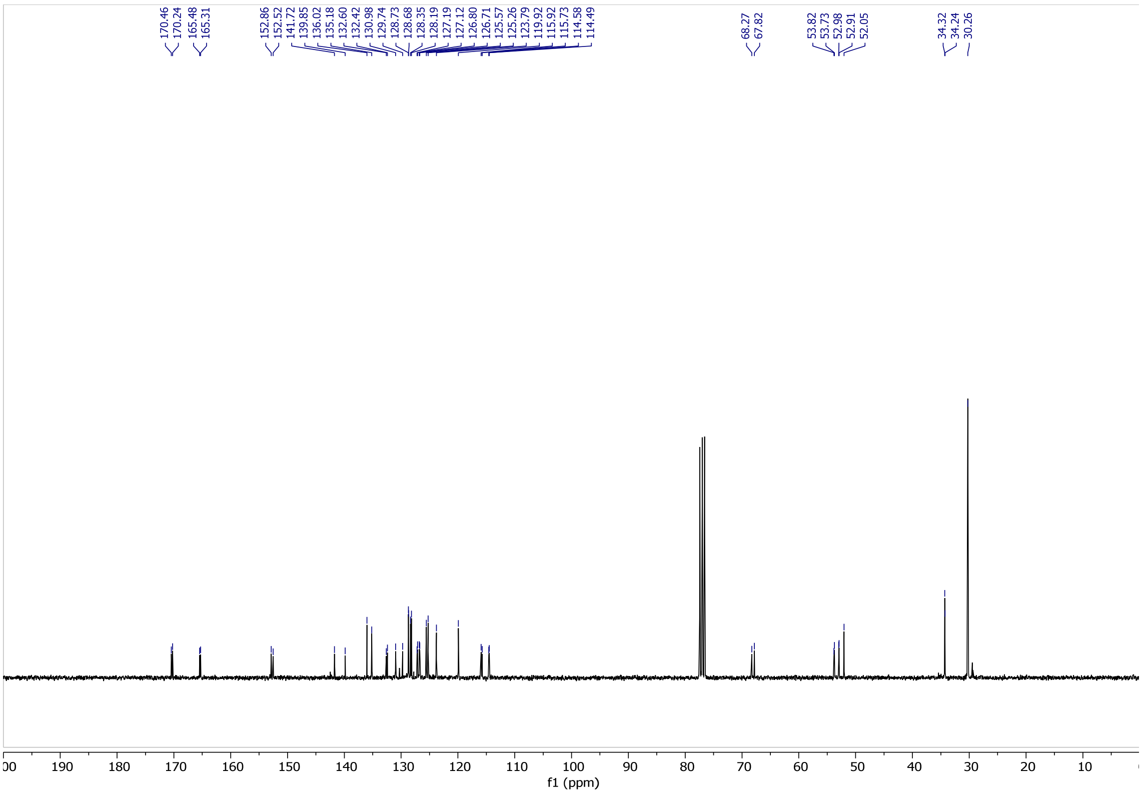

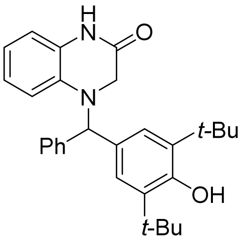

4ea
^{1}H NMR ($CDCl_3$, 300 MHz)
^{13}C {^{1}H}-NMR ($CDCl_3$, 75 MHz)

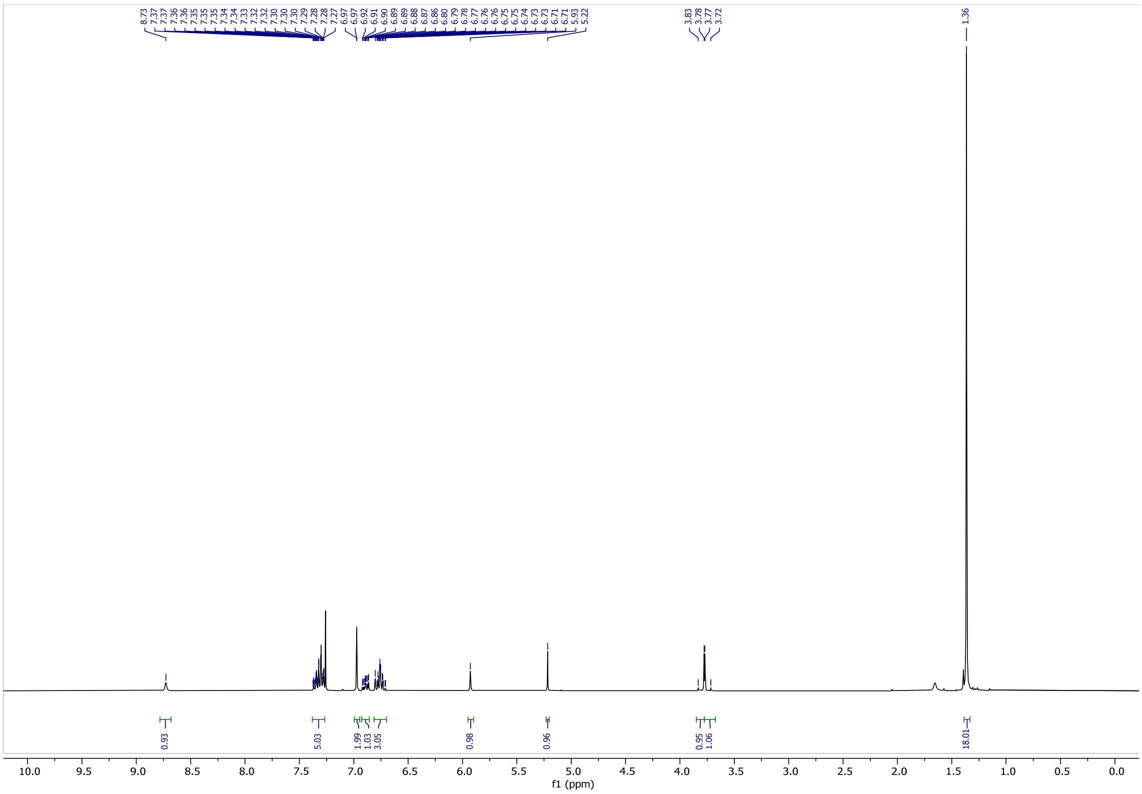

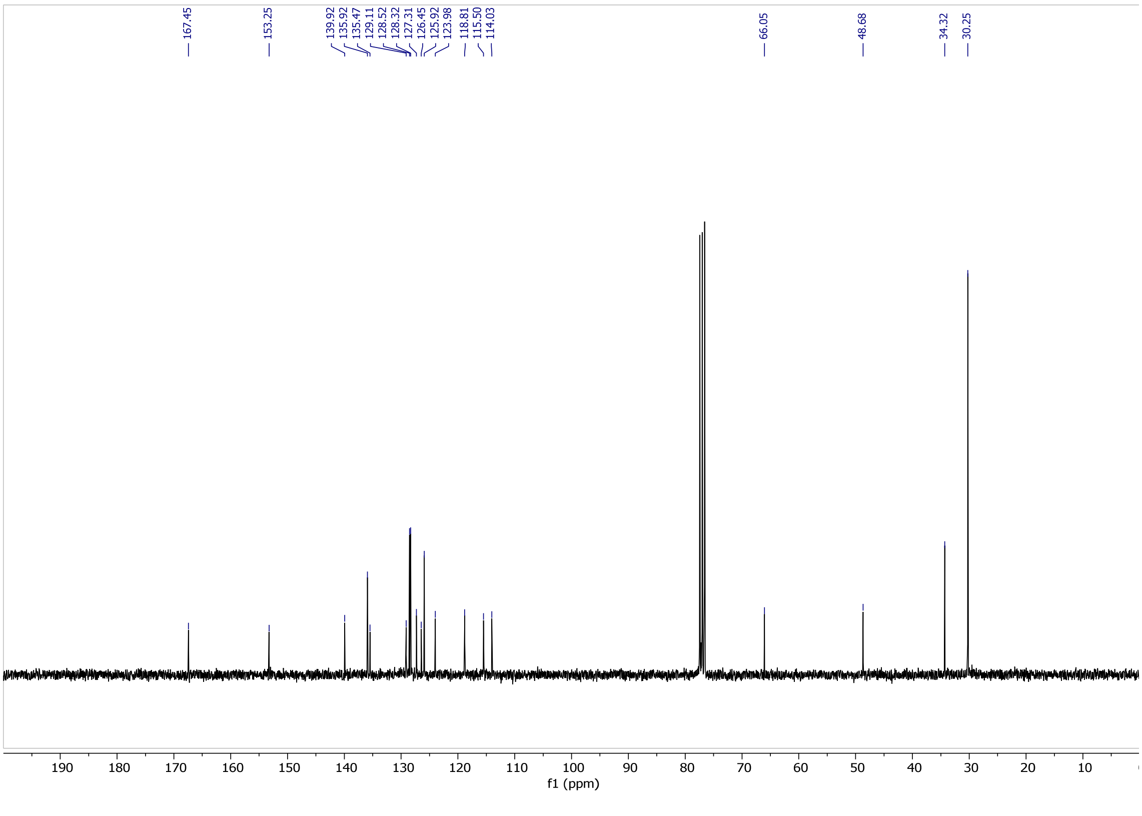

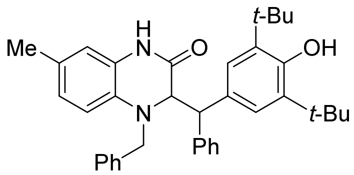

3fa'

^{1}H NMR (CDCl$_3$, 300 MHz)

^{13}C {^{1}H}-NMR (CDCl$_3$, 75 MHz)

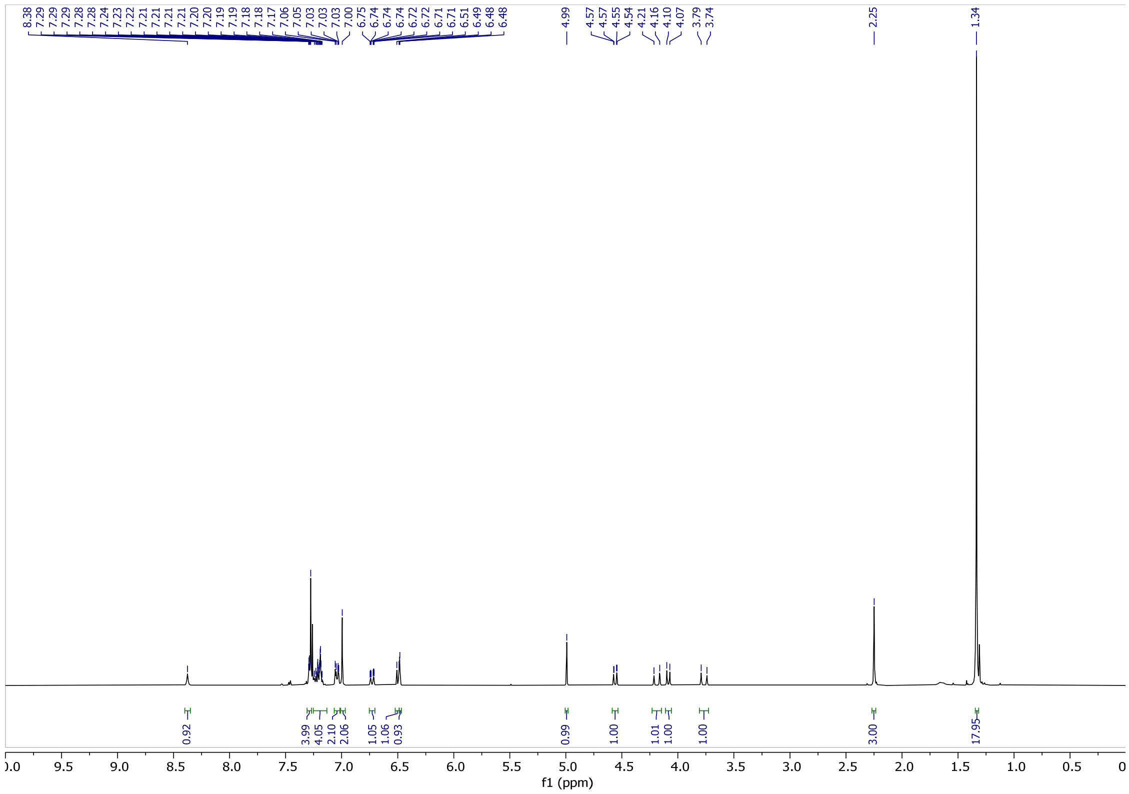

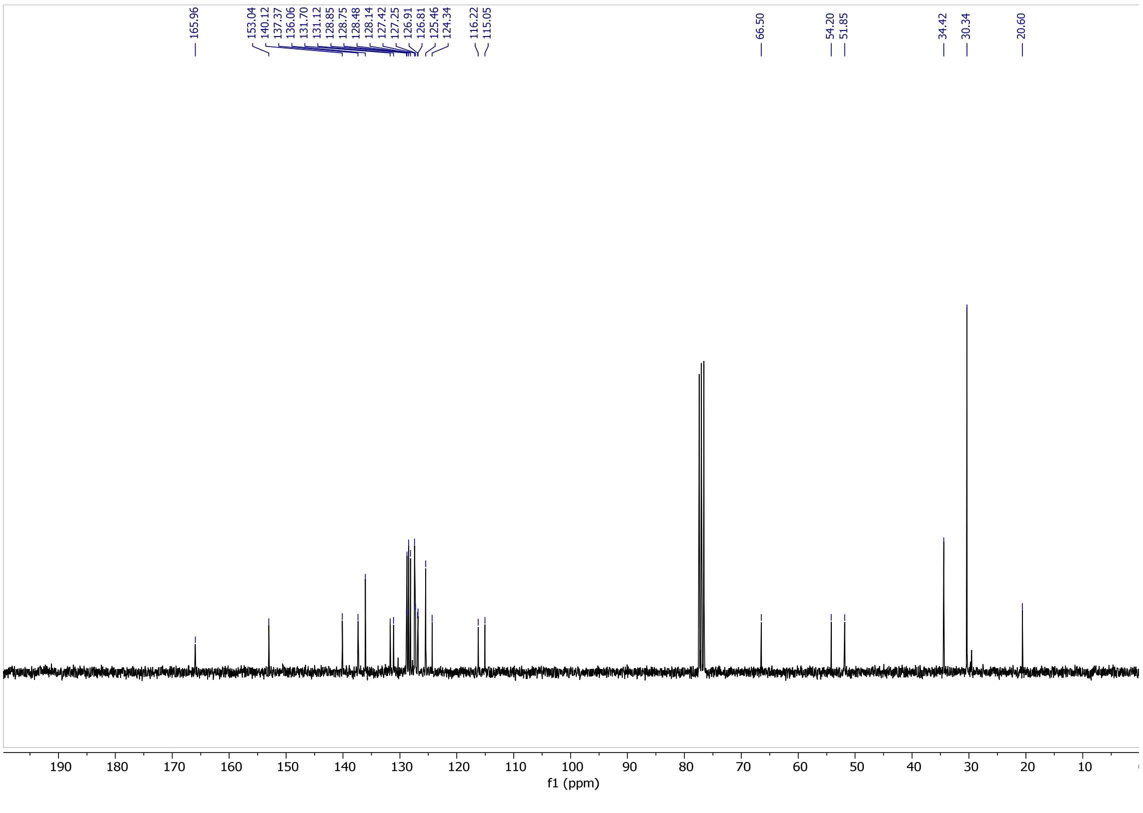

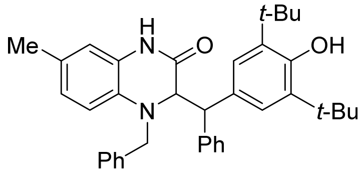

3fa''

^{1}H NMR ($CDCl_3$, 300 MHz)

^{13}C {^{1}H}-NMR ($CDCl_3$, 75 MHz)

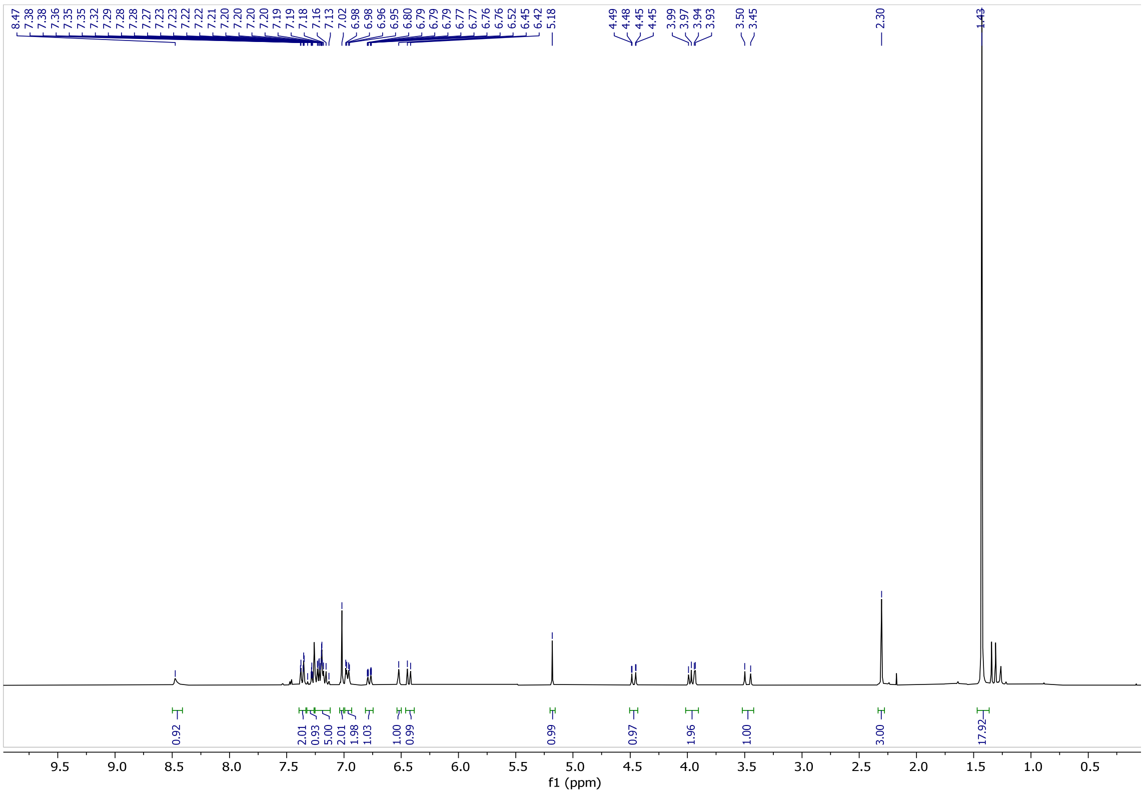

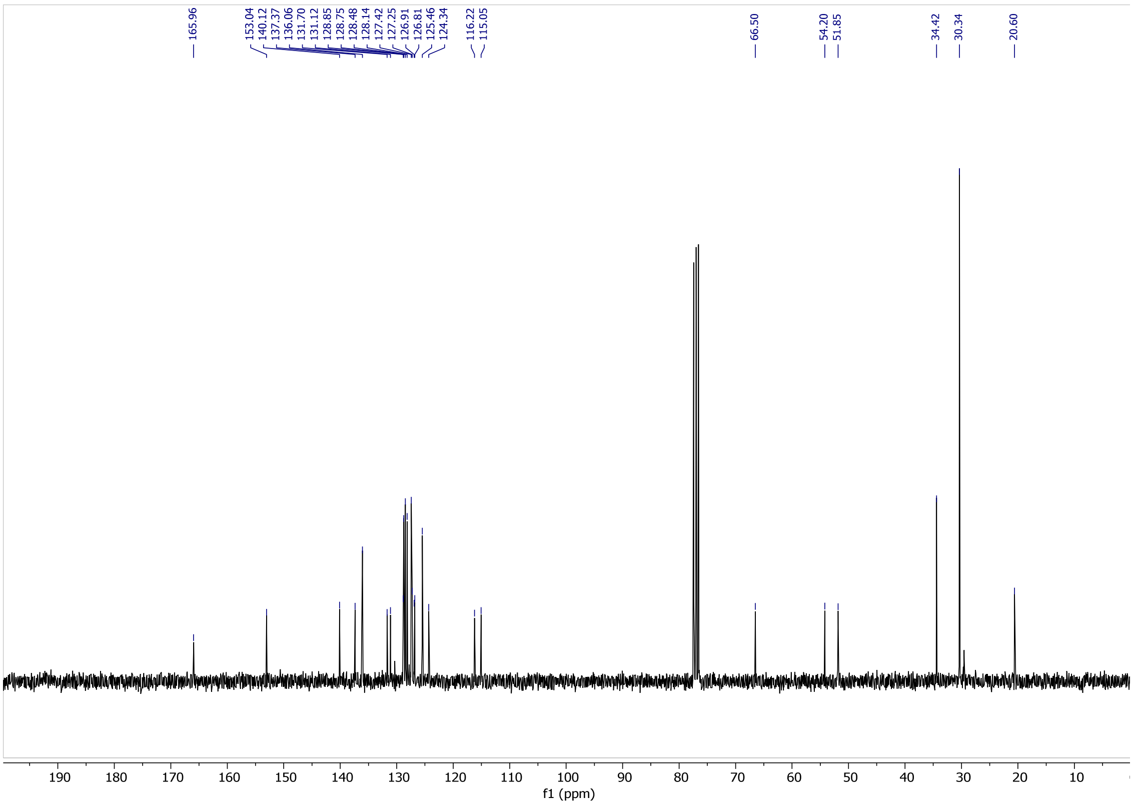

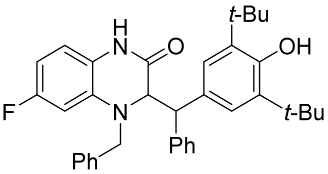

3ga'
^{1}H NMR ($CDCl_3$, 300 MHz)
^{13}C {^{1}H}-NMR ($CDCl_3$, 75 MHz)

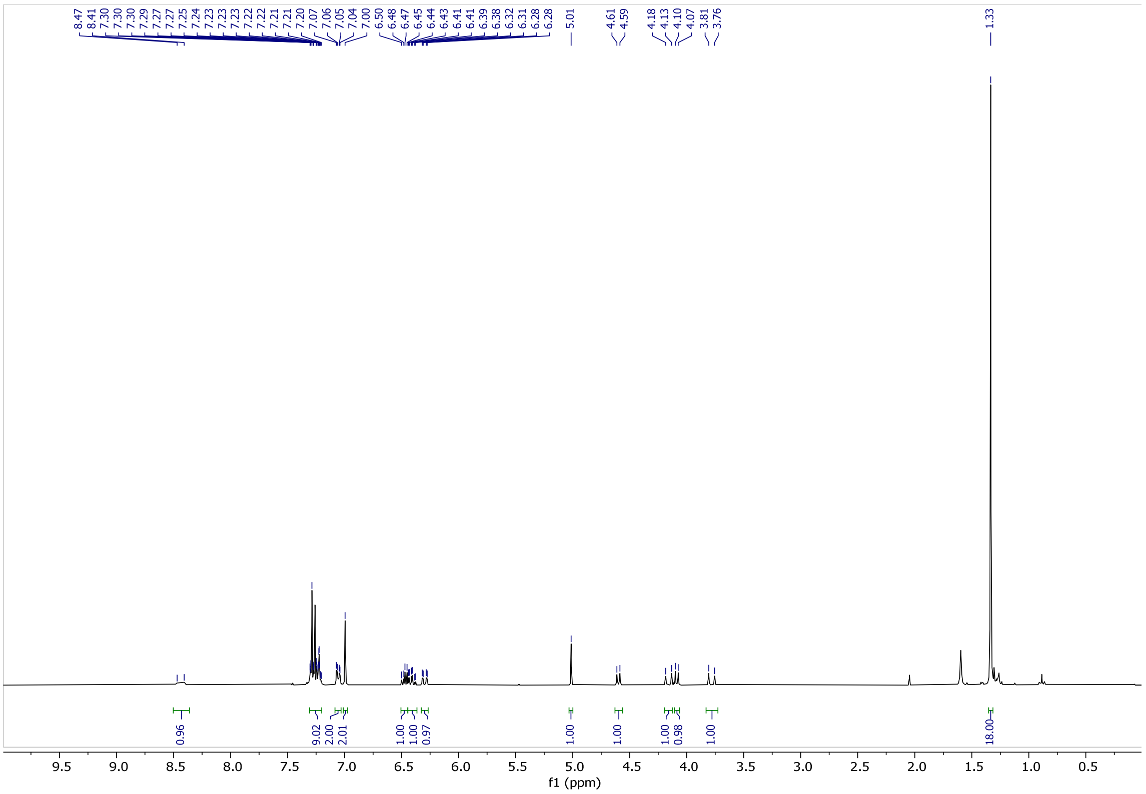

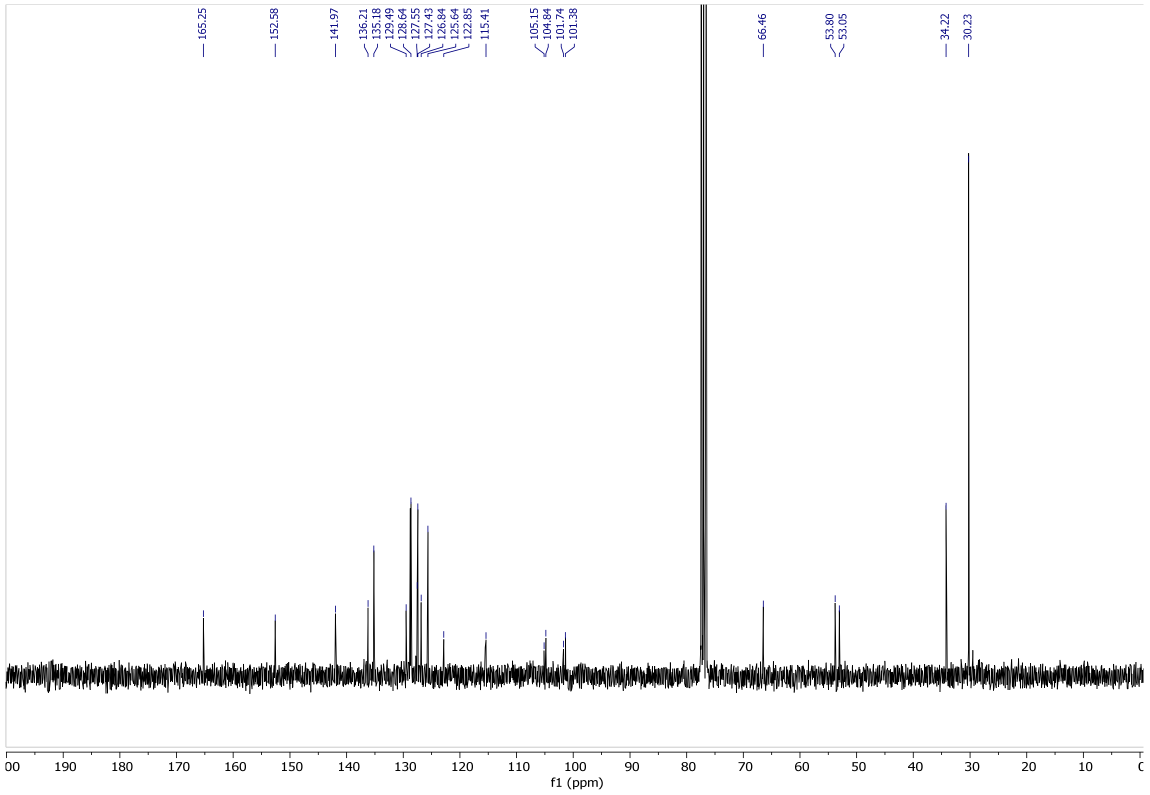

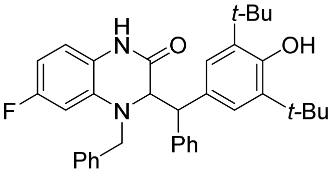

3ga''
^{1}H NMR (CDCl$_3$, 300 MHz)
^{13}C {^{1}H}-NMR (CDCl$_3$, 75 MHz)

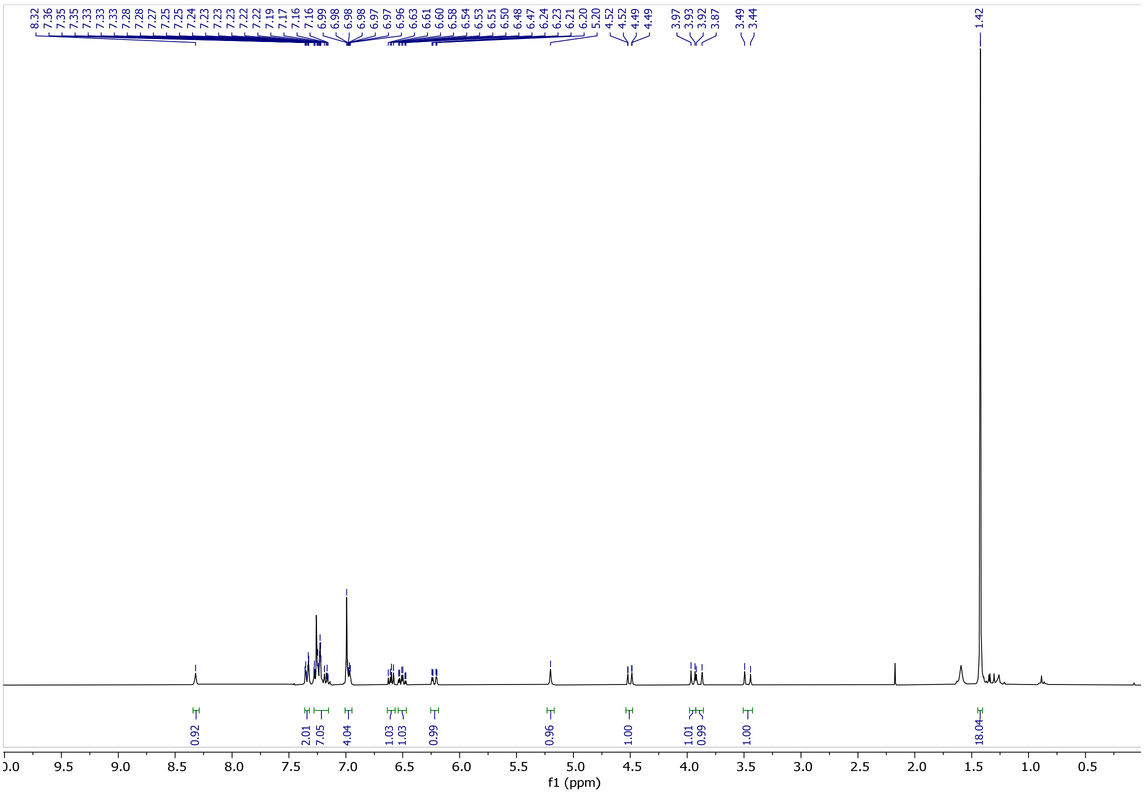

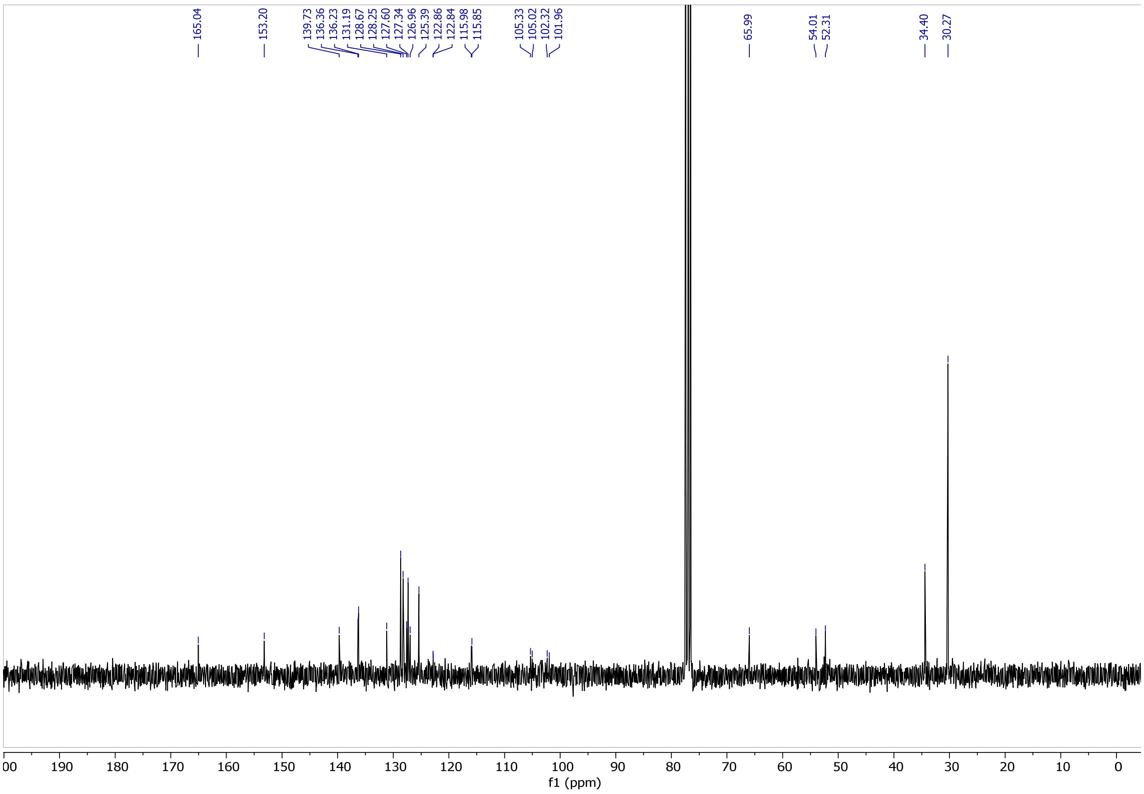

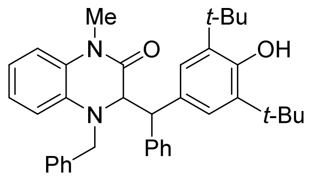

3ha'
^{1}H NMR ($CDCl_3$, 300 MHz)
^{13}C {^{1}H}-NMR ($CDCl_3$, 75 MHz)

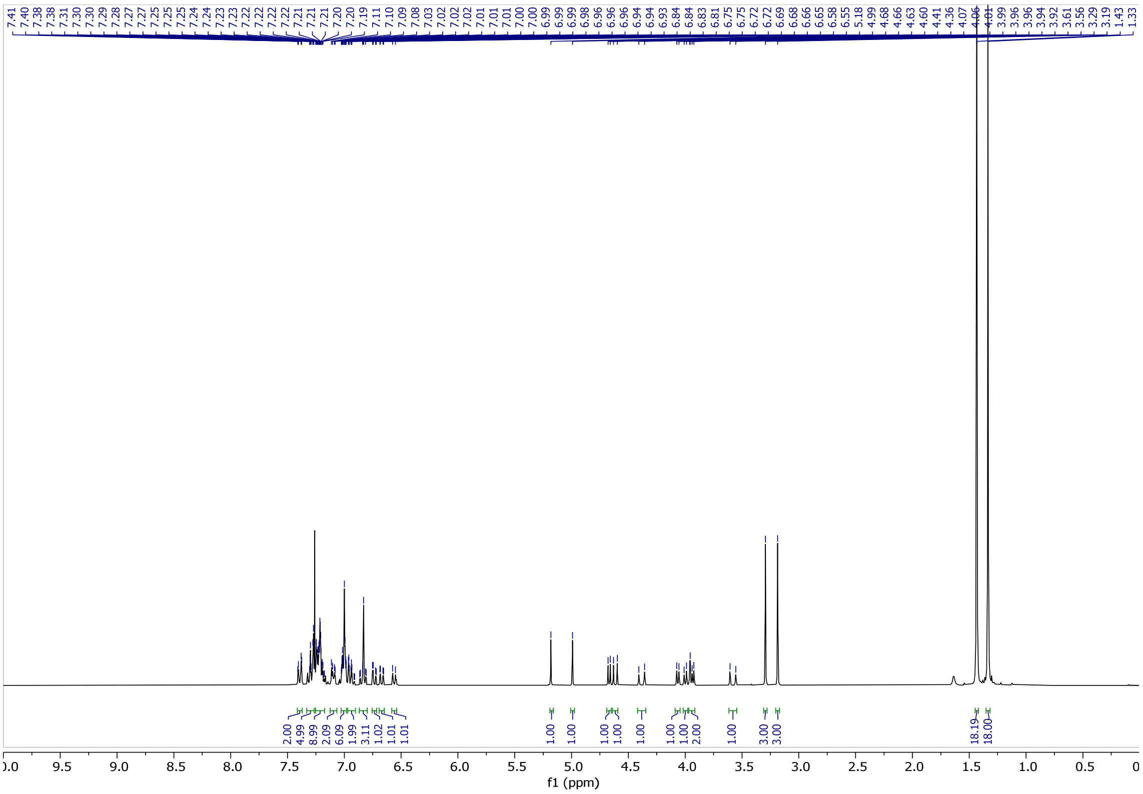

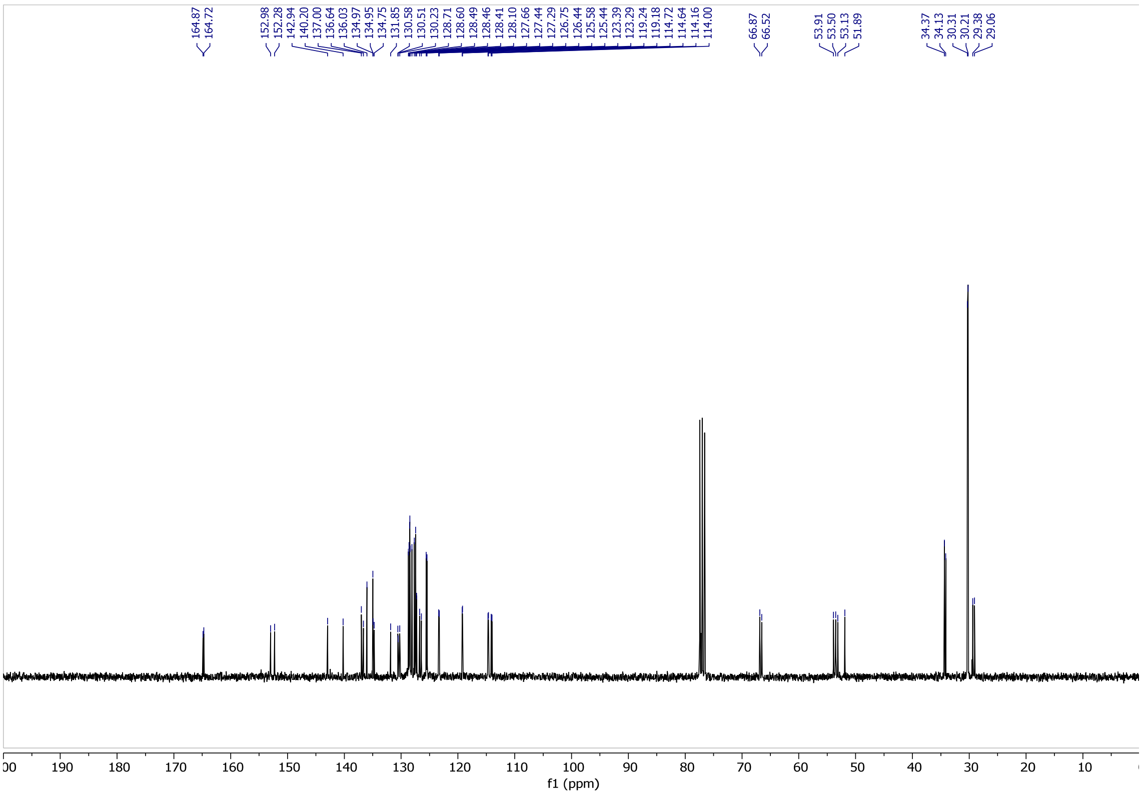

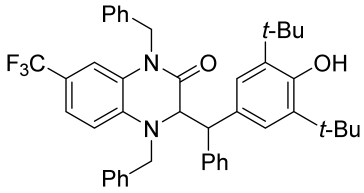

3ia'

^{1}H NMR (CDCl$_3$, 500 MHz)

^{13}C {^{1}H}-NMR (CDCl$_3$, 126 MHz)

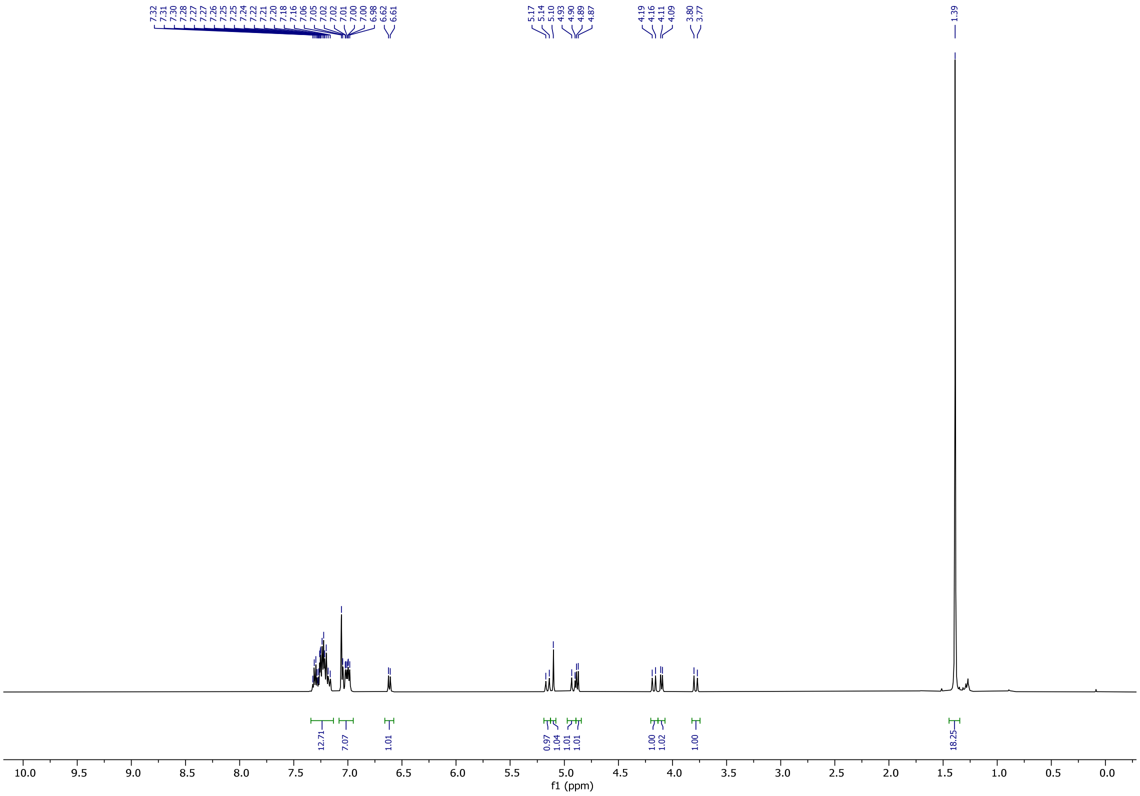

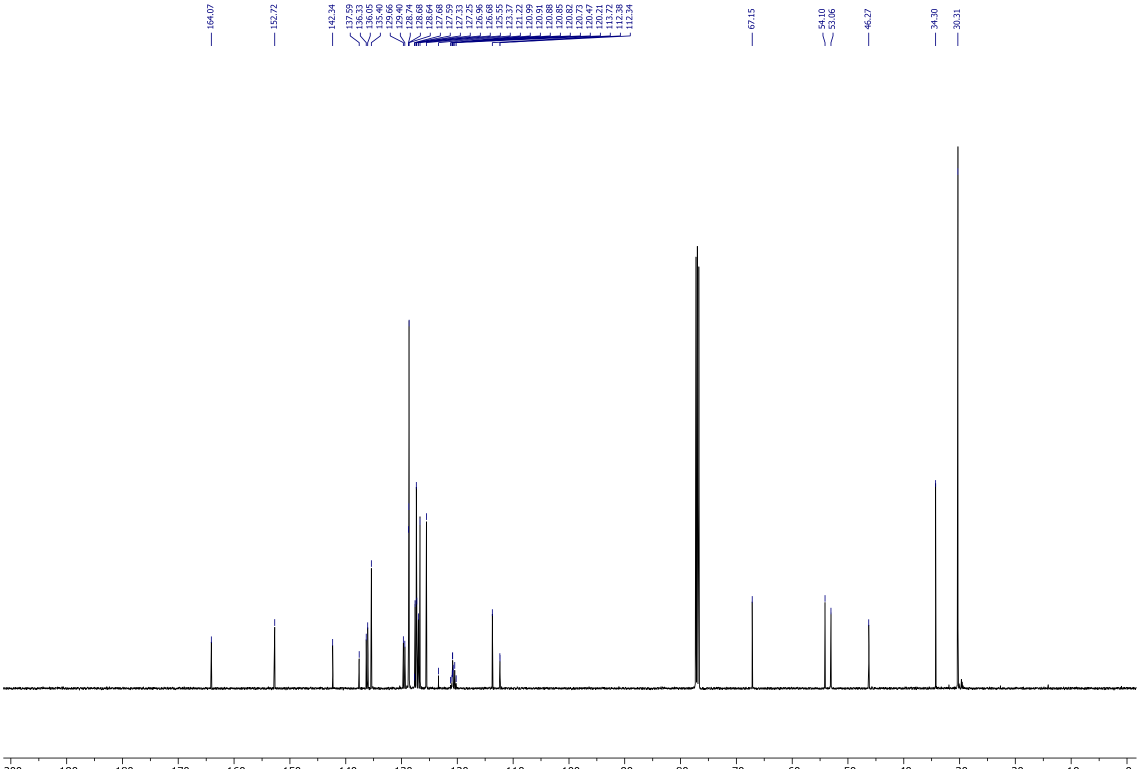

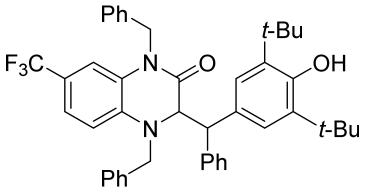

3ia''

^{1}H NMR ($CDCl_3$, 500 MHz)

^{13}C {^{1}H}-NMR ($CDCl_3$, 126 MHz)

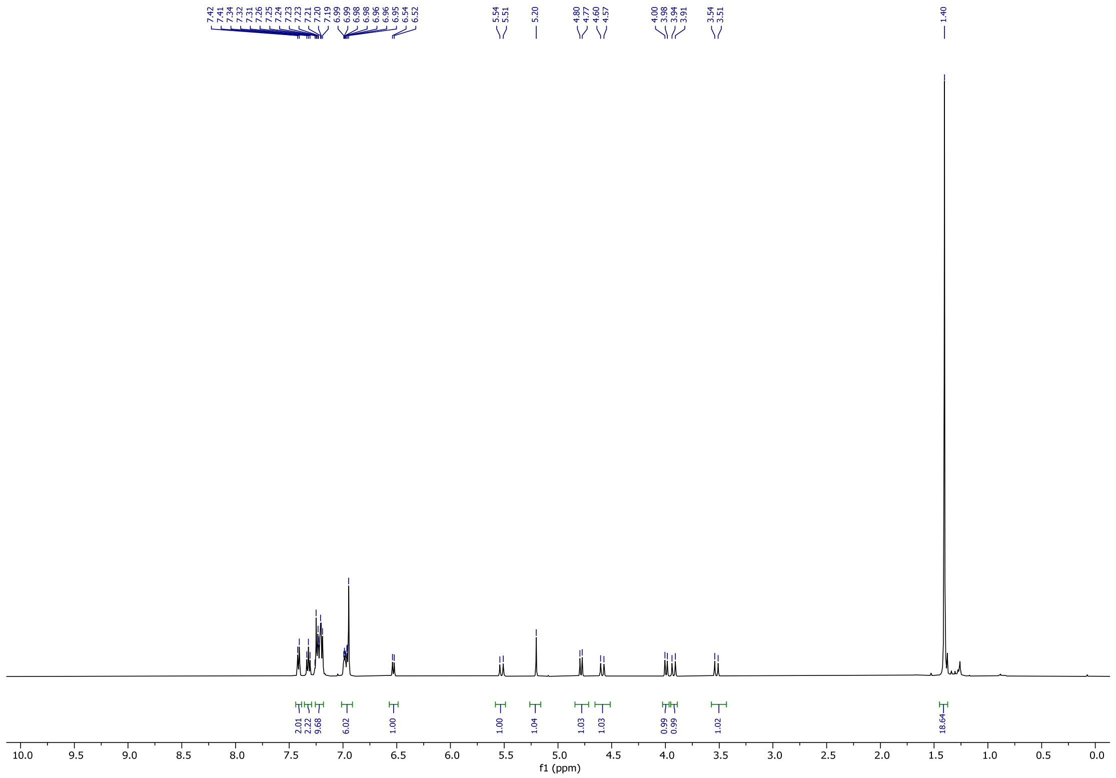

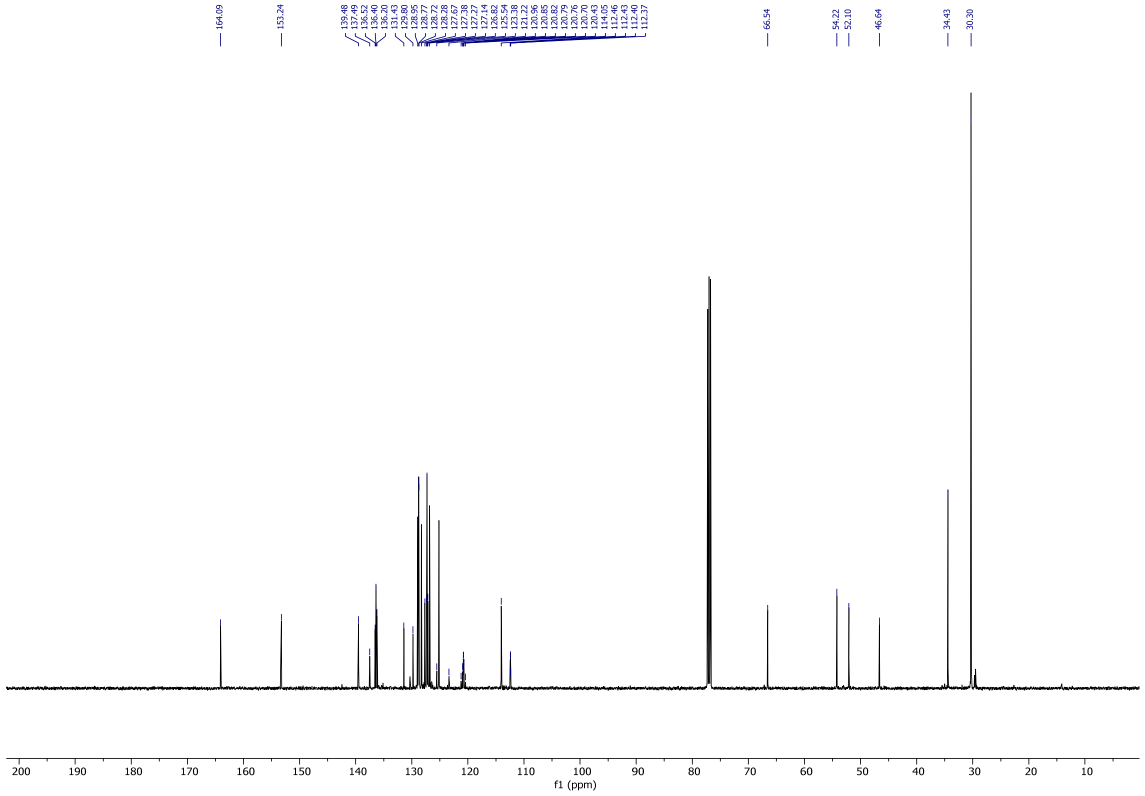

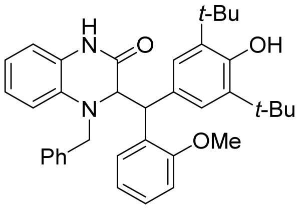

3ab'
^{1}H NMR ($CDCl_3$, 300 MHz)
^{13}C {^{1}H}-NMR ($CDCl_3$, 75 MHz)

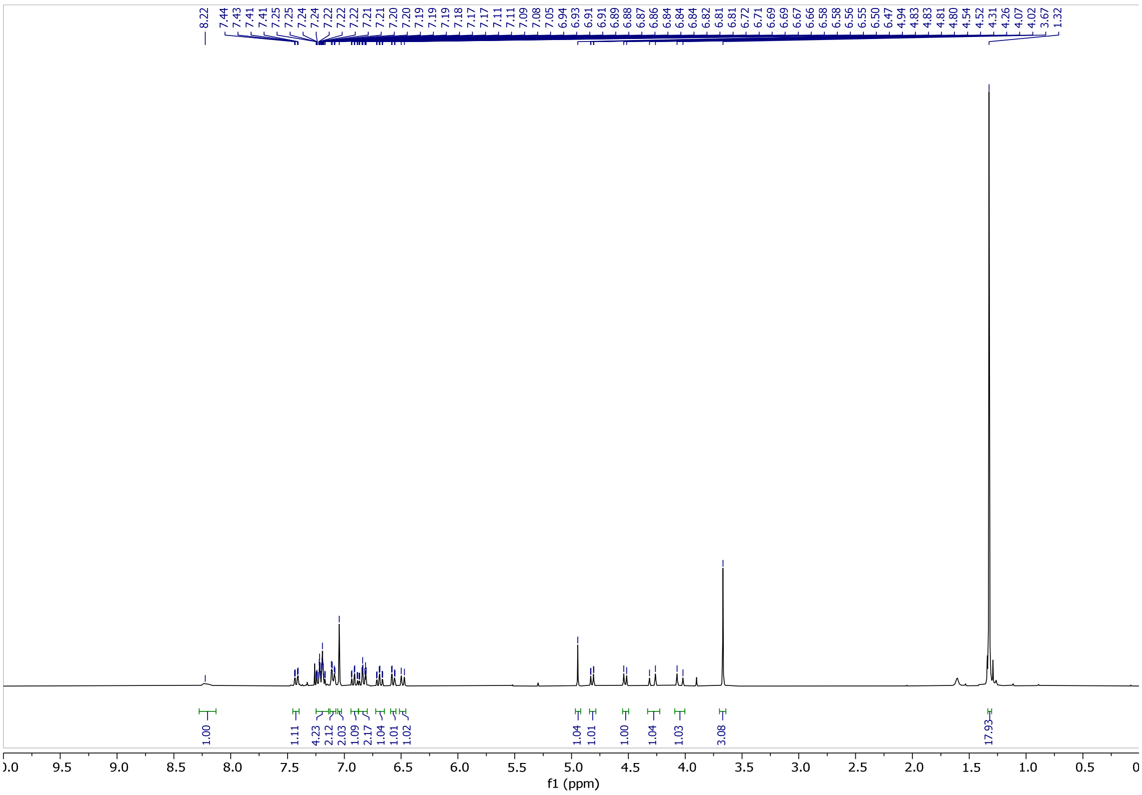

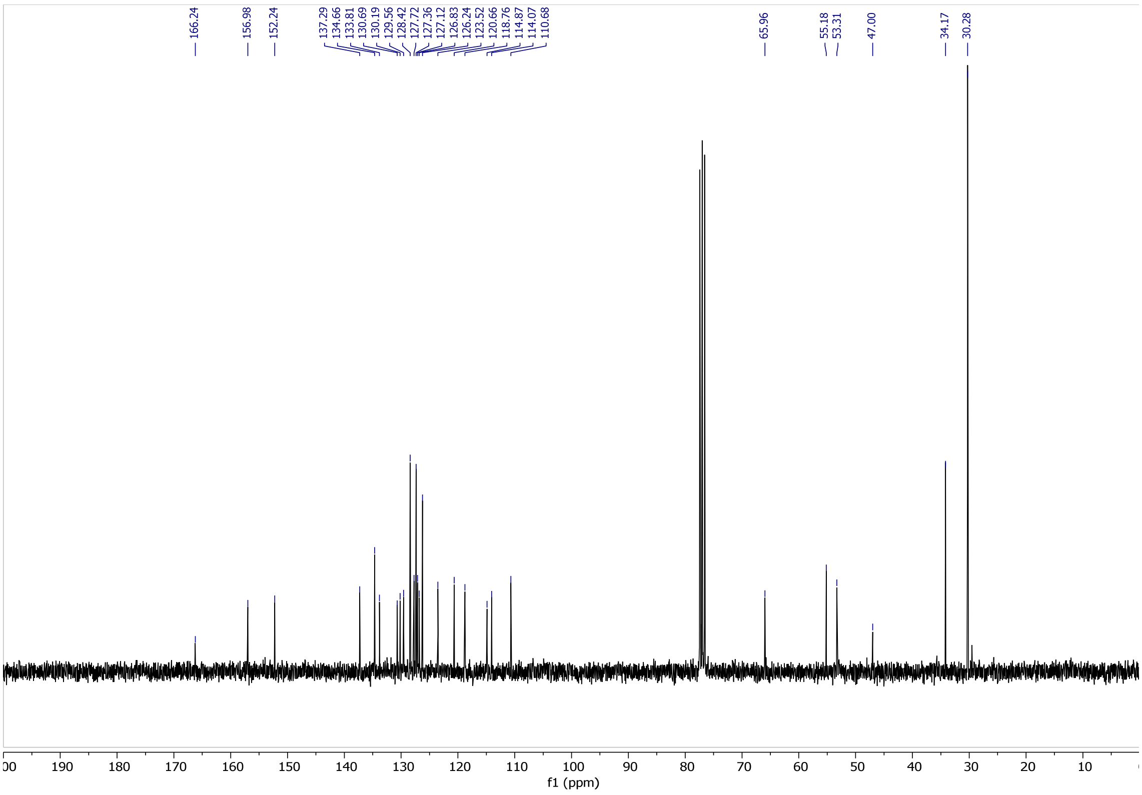

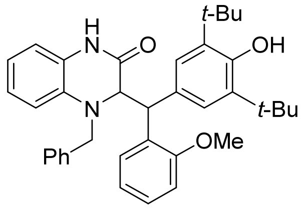

3ab'

^{1}H NMR ($CDCl_3$, 300 MHz)

^{13}C {^{1}H}-NMR ($CDCl_3$, 75 MHz)

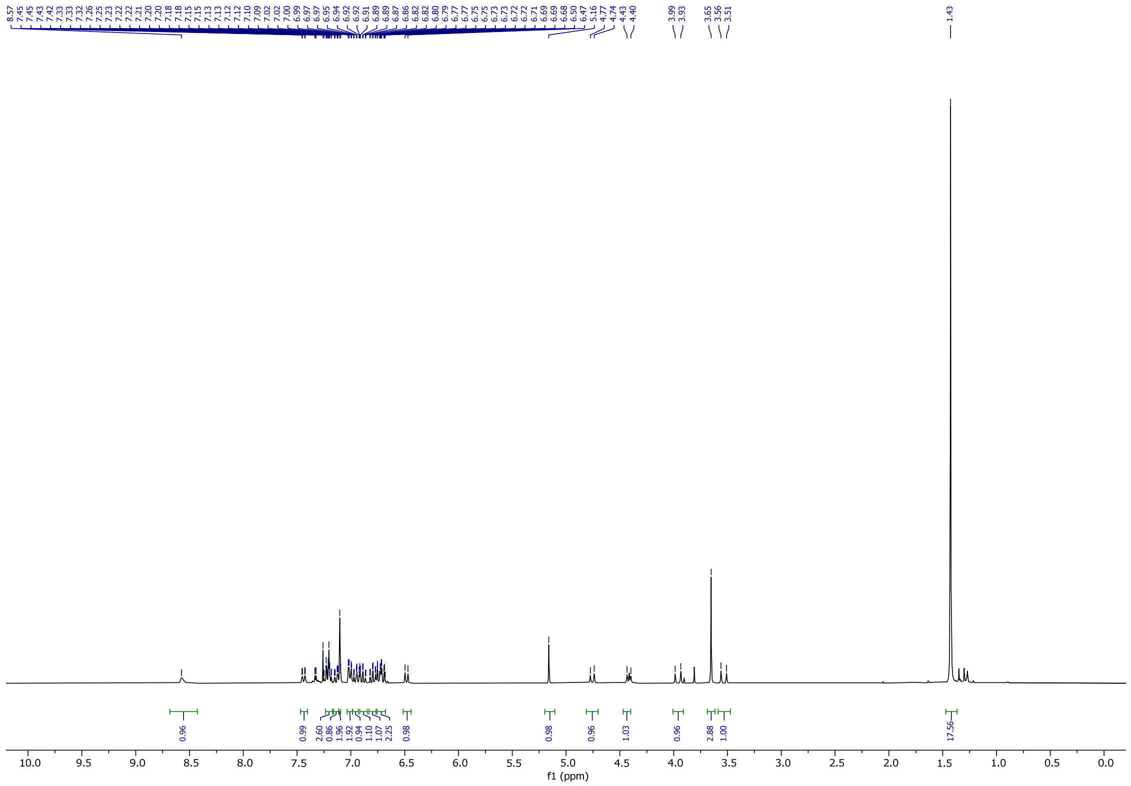

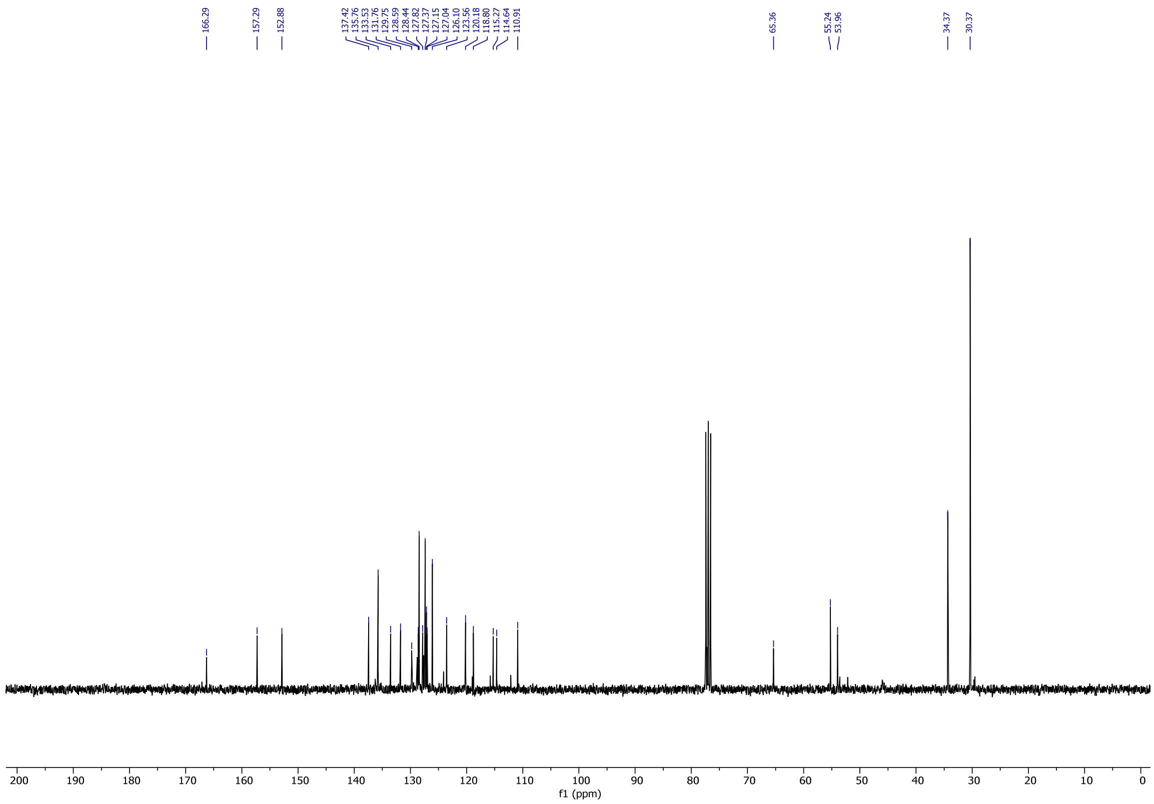

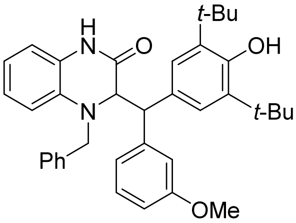

3ac'

^{1}H NMR ($CDCl_3$, 300 MHz)

^{13}C {^{1}H}-NMR ($CDCl_3$, 75 MHz)

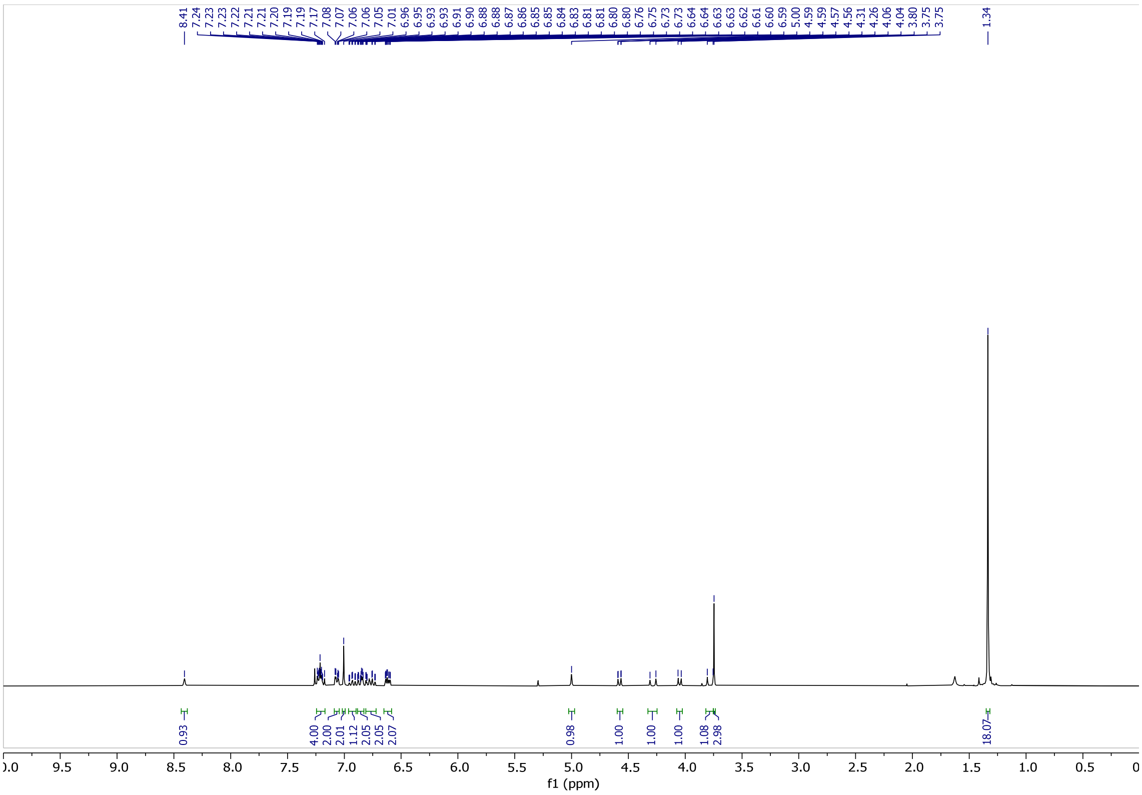

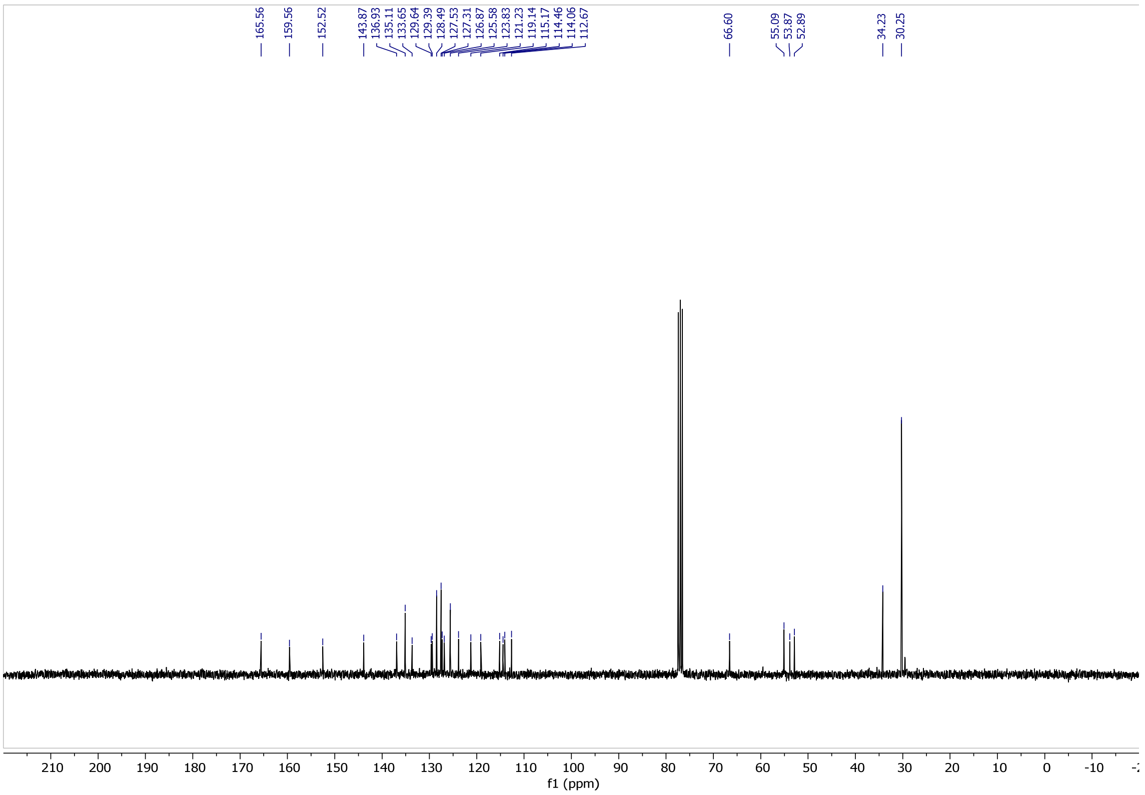

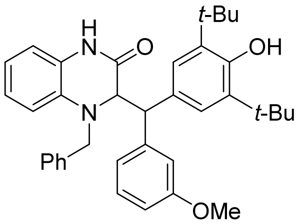

3ac''
^{1}H NMR ($CDCl_3$, 300 MHz)
^{13}C {^{1}H}-NMR ($CDCl_3$, 75 MHz)

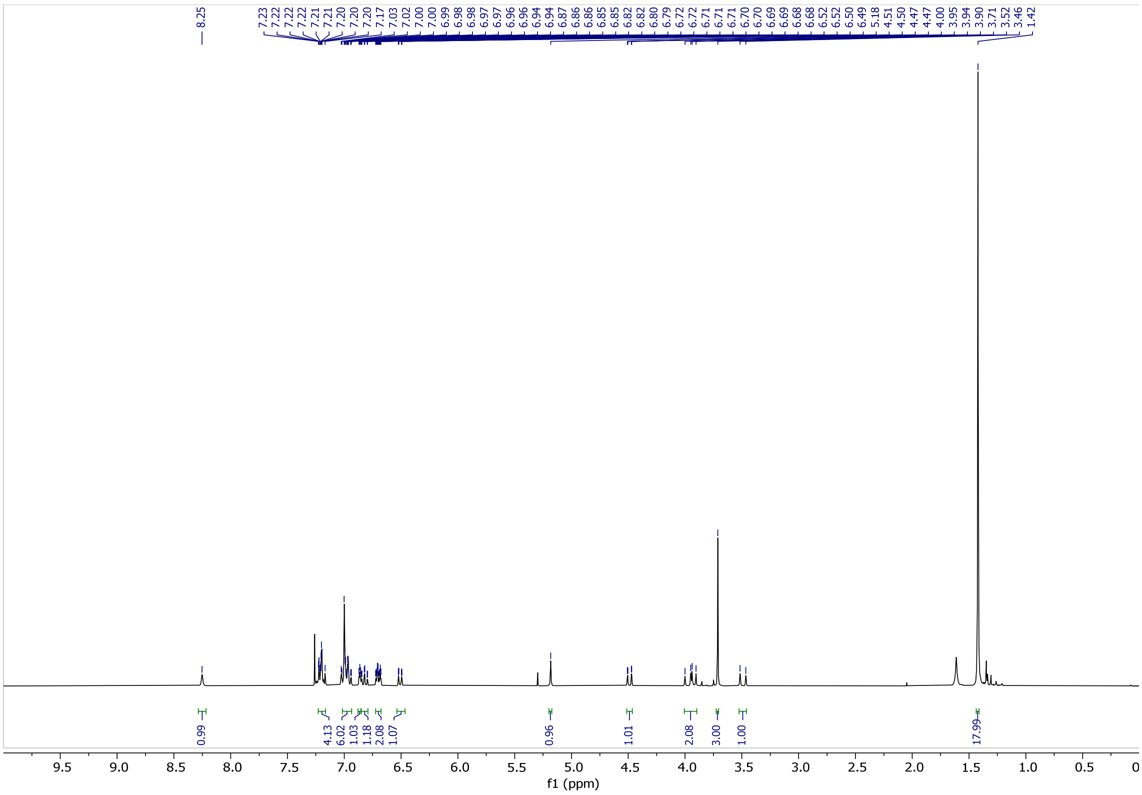

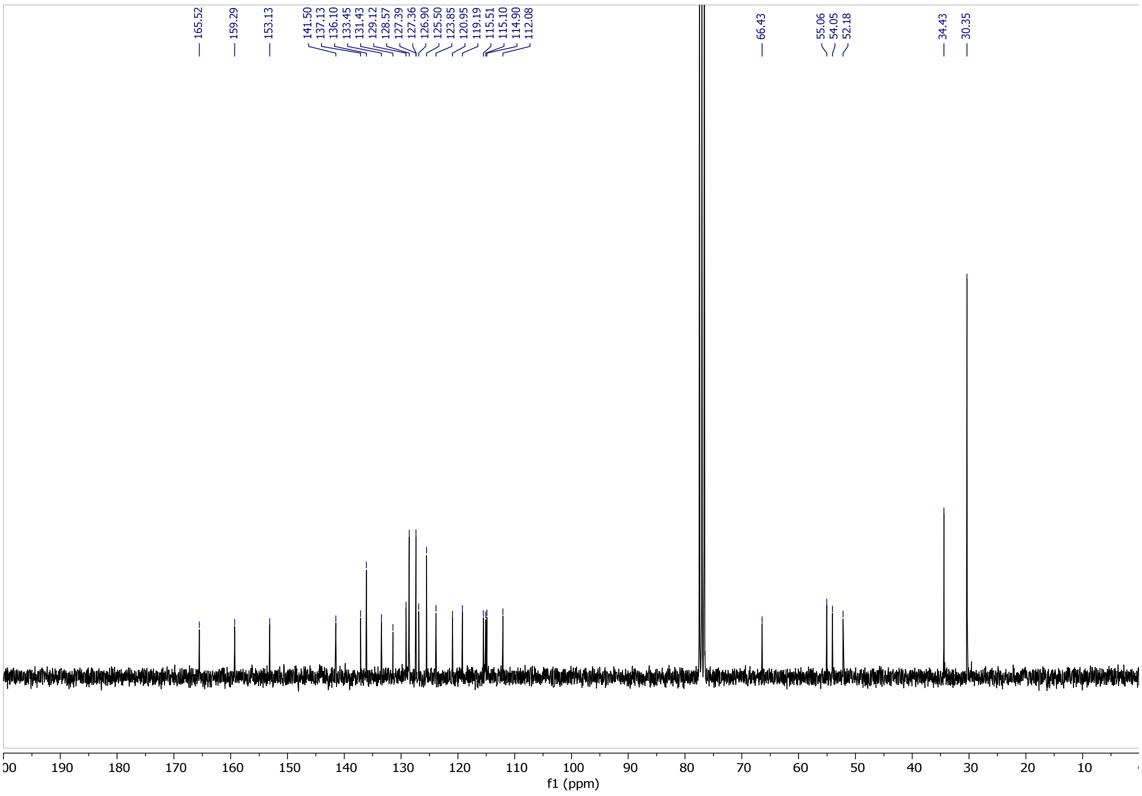

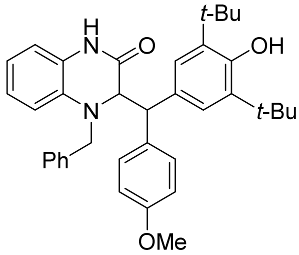

3ad'
^{1}H NMR ($CDCl_3$, 300 MHz)
^{13}C {^{1}H}-NMR ($CDCl_3$, 75 MHz)

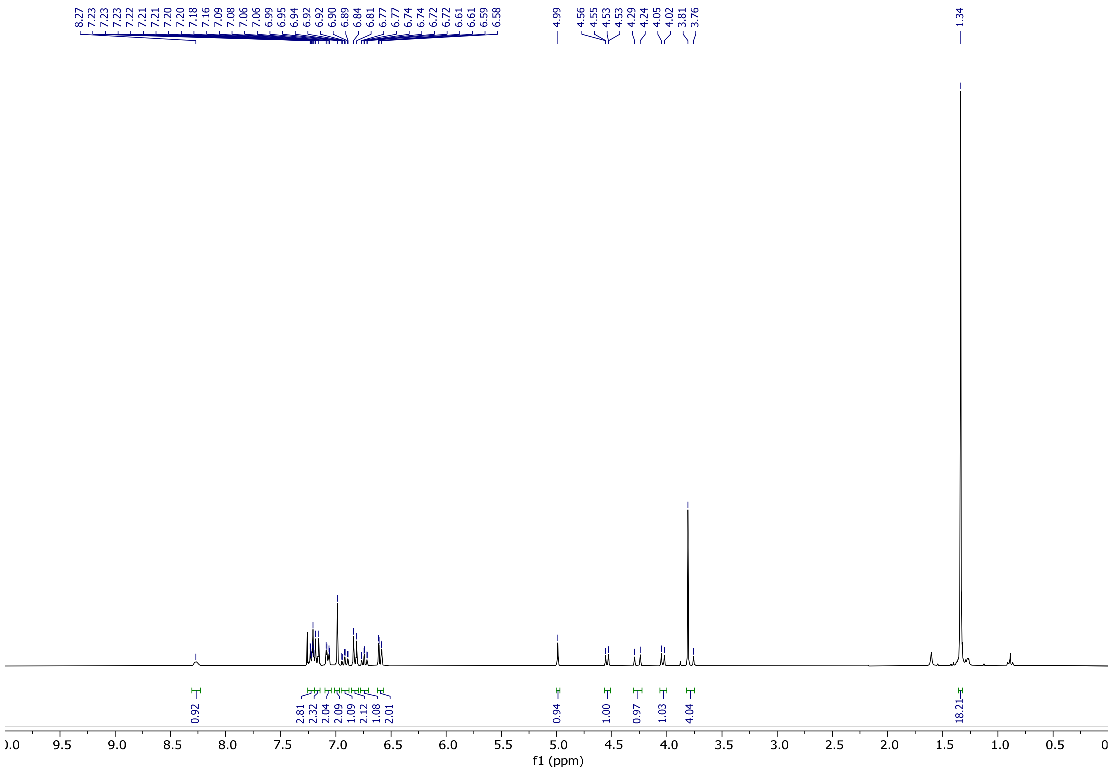

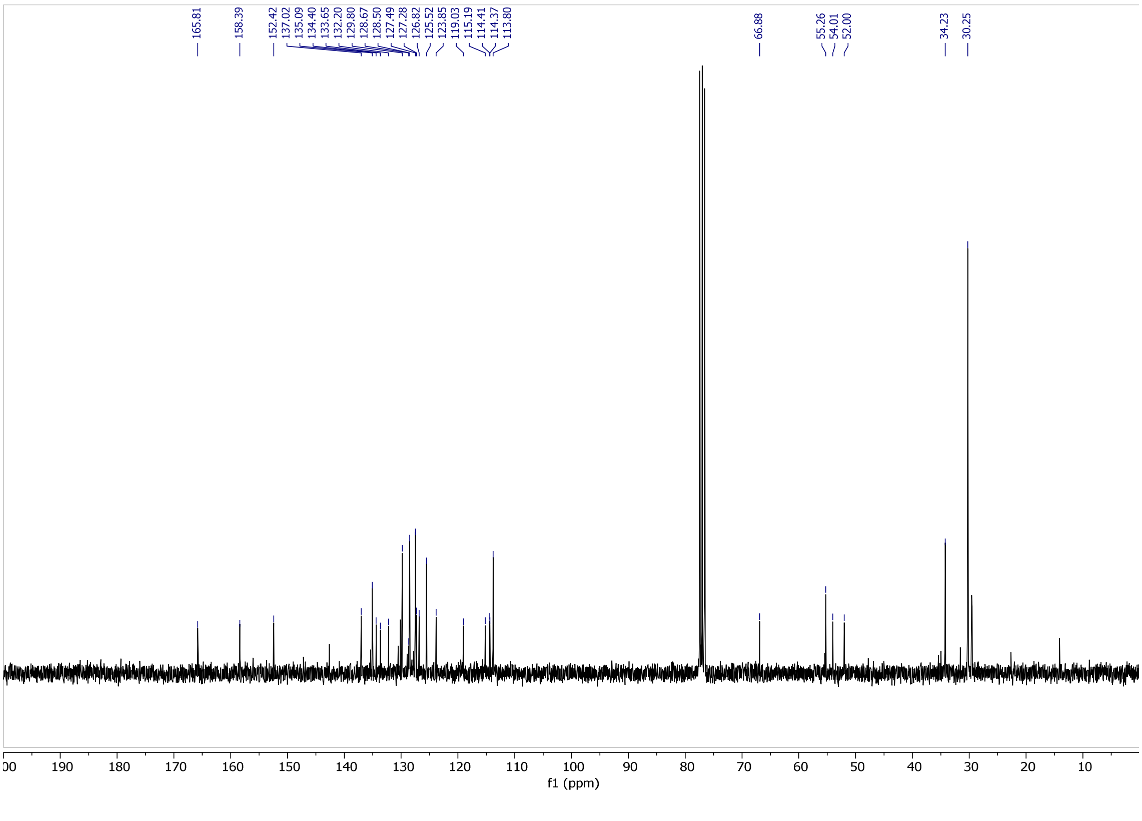

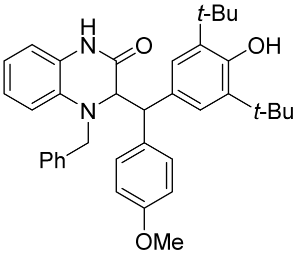

3ad"

^{1}H NMR (CDCl$_3$, 300 MHz)

^{13}C {^{1}H}-NMR (CDCl$_3$, 75 MHz)

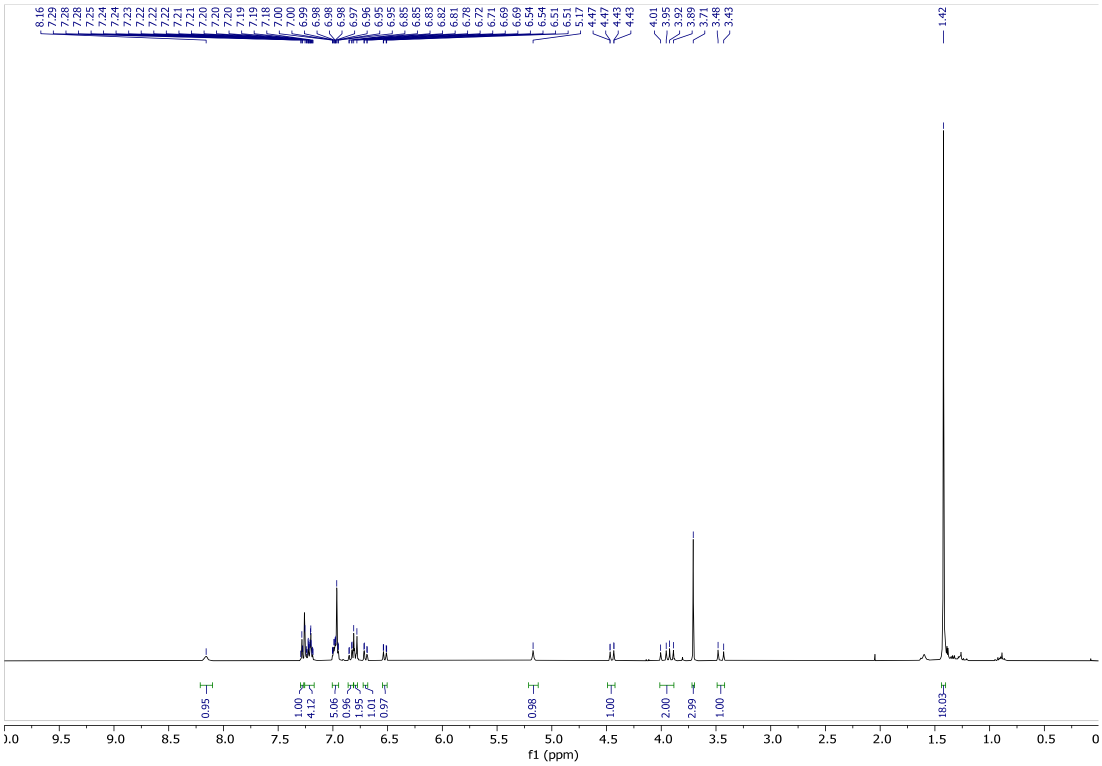

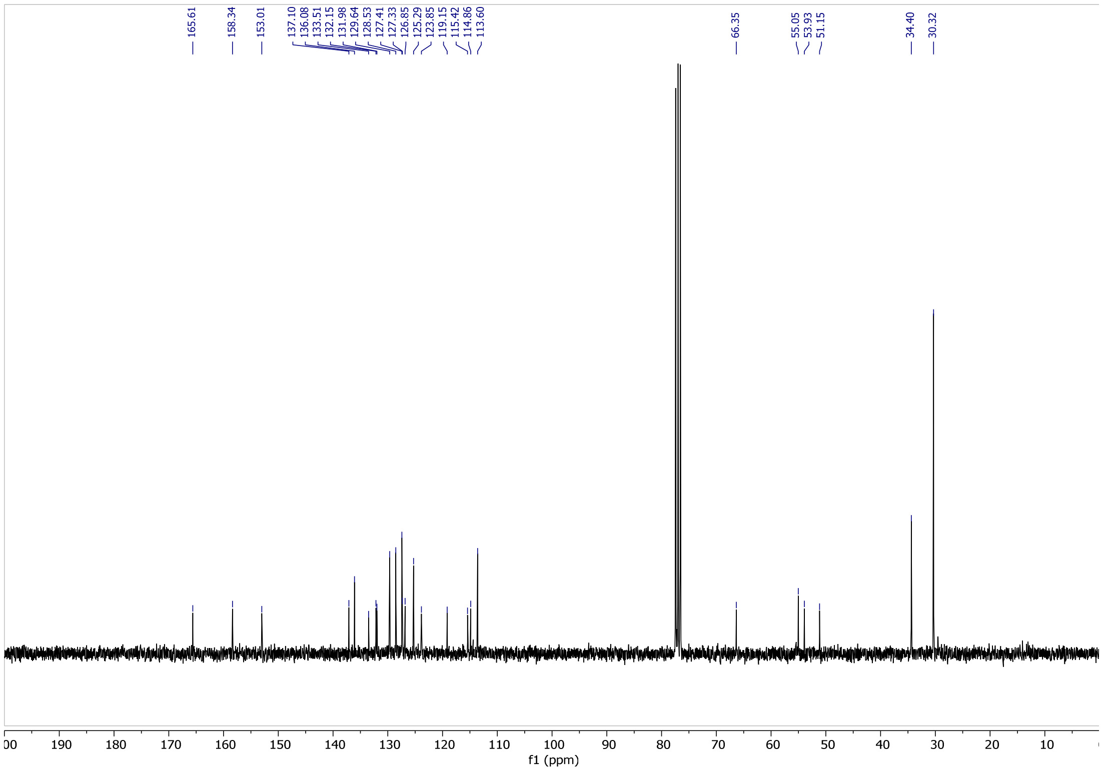

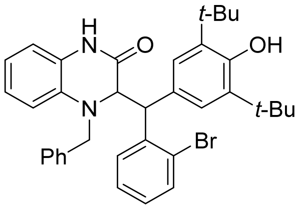

3ae

^{1}H NMR ($CDCl_3$, 300 MHz)

^{13}C {^{1}H}-NMR ($CDCl_3$, 75 MHz)

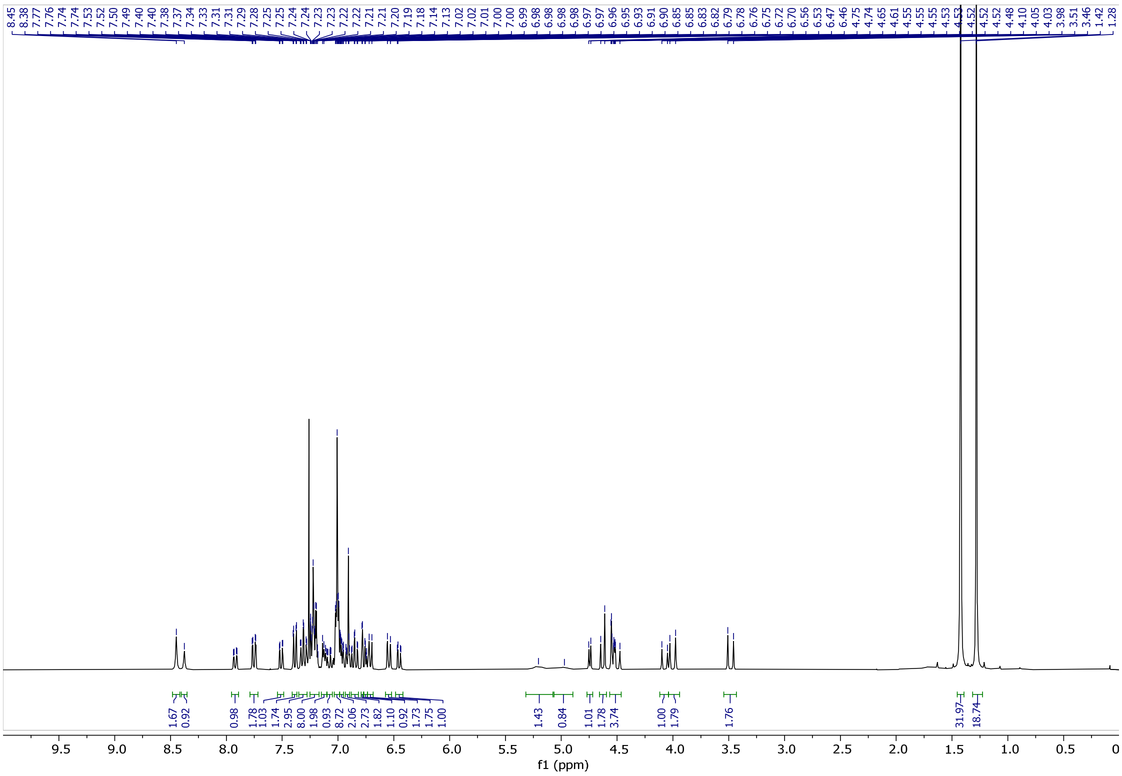

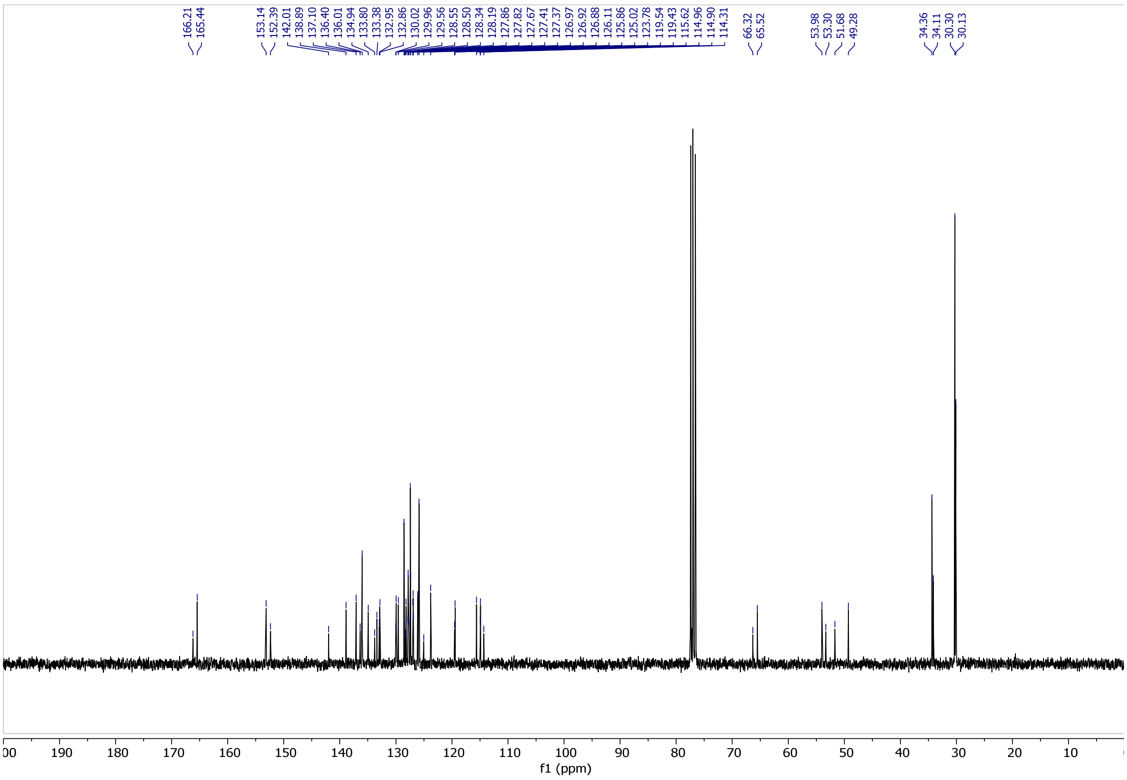

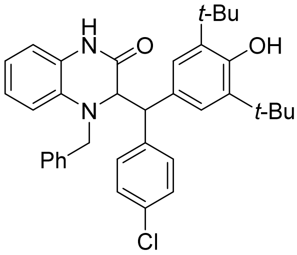

3af'

^{1}H NMR ($CDCl_3$, 300 MHz)

^{13}C {^{1}H}-NMR ($CDCl_3$, 75 MHz)

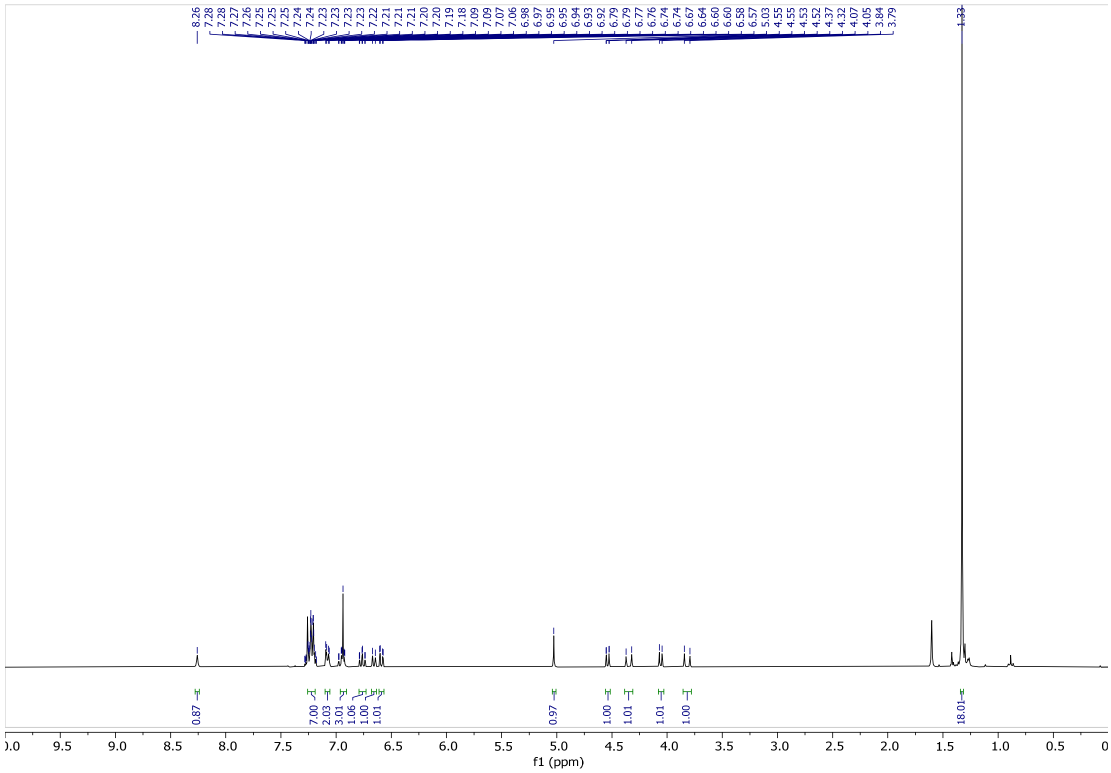

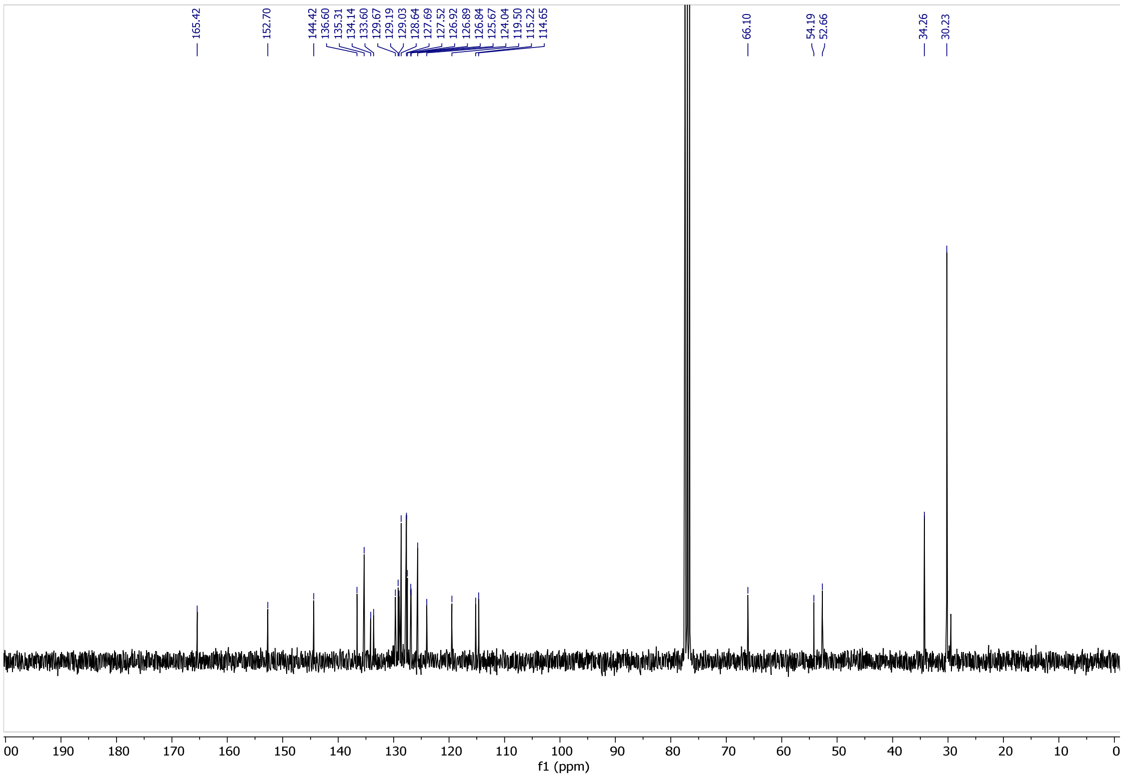

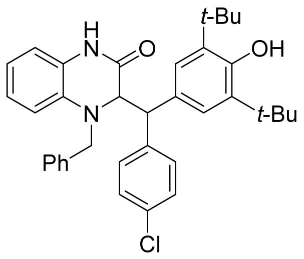

3af''
^{1}H NMR ($CDCl_3$, 300 MHz)
^{13}C {^{1}H}-NMR ($CDCl_3$, 75 MHz)

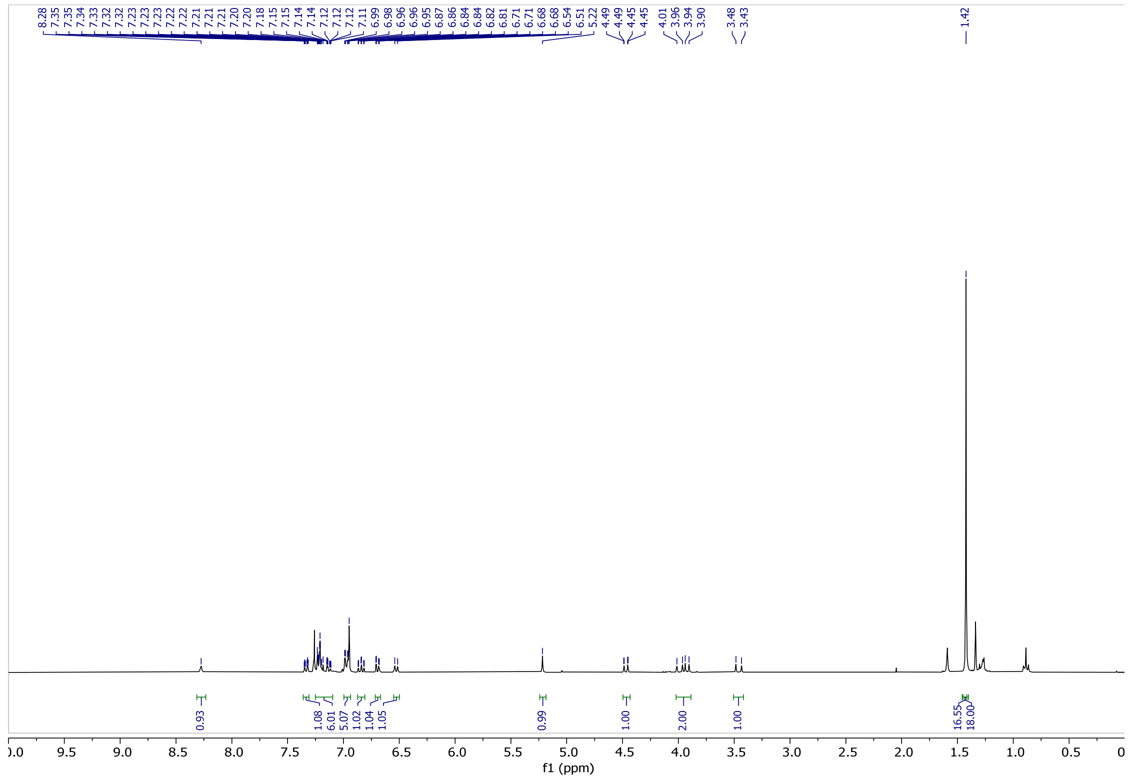

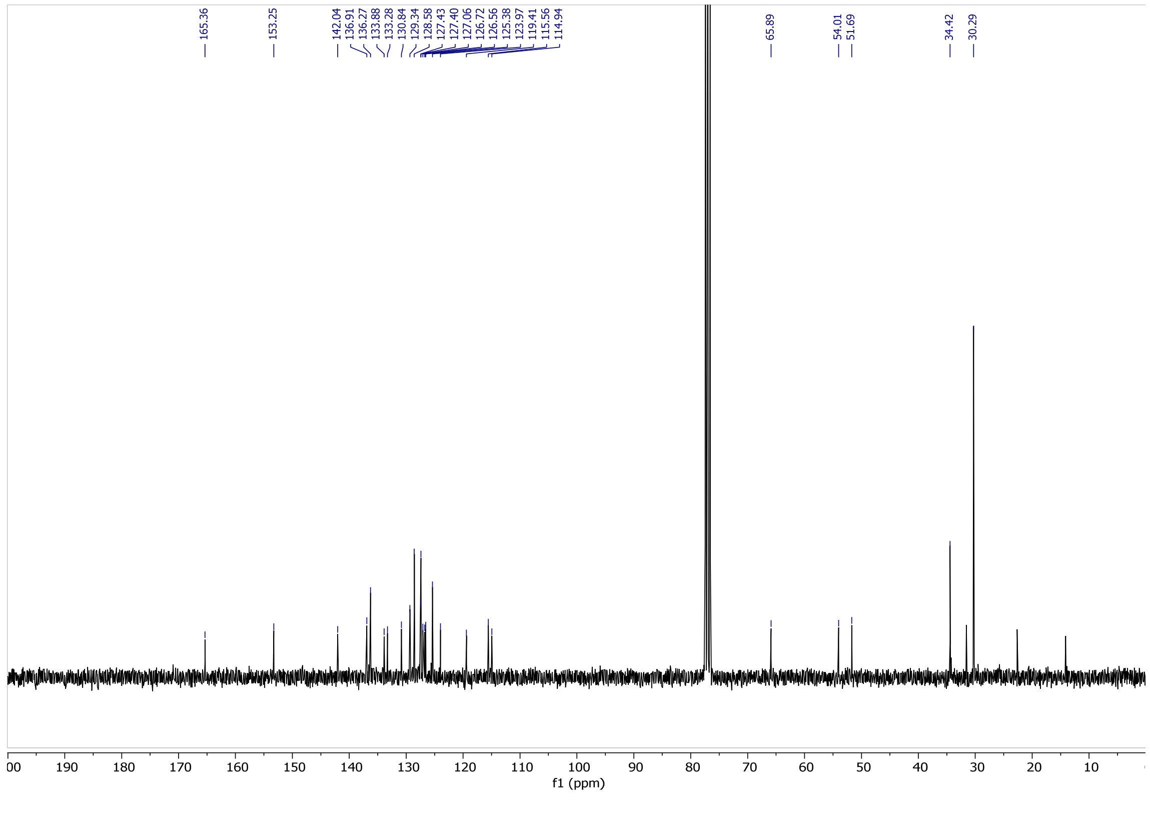

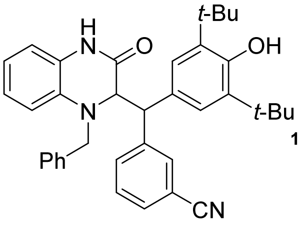

3ag'

^{1}H NMR ($CDCl_3$, 500 MHz)

^{13}C {^{1}H}-NMR ($CDCl_3$, 126 MHz)

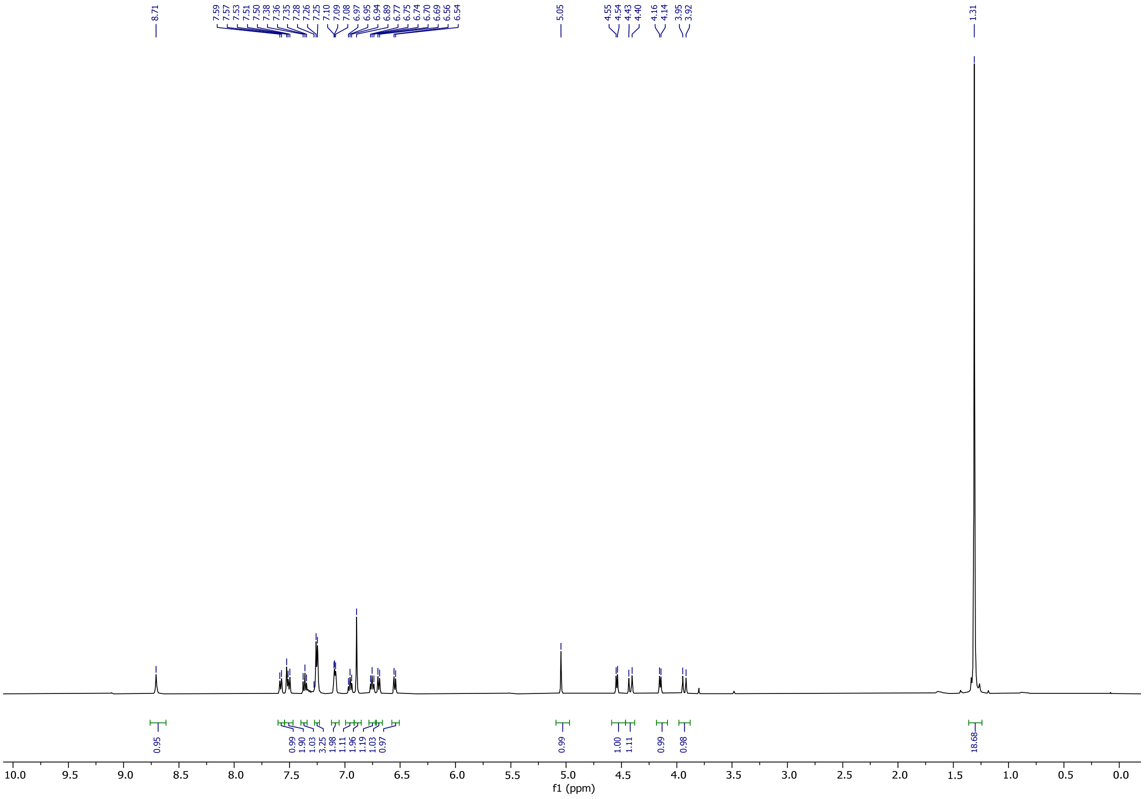

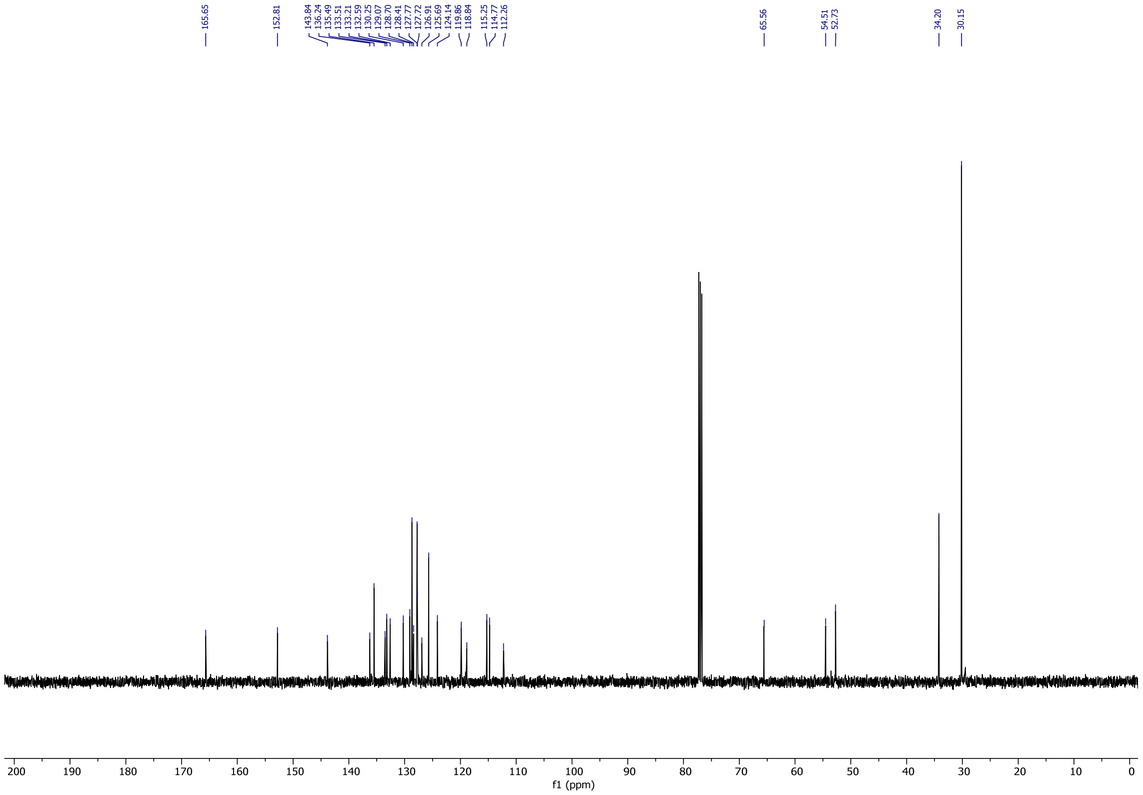

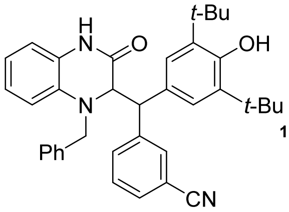

3ag''

^{1}H NMR (CDCl$_3$, 500 MHz)

^{13}C {^{1}H}-NMR (CDCl$_3$, 126 MHz)

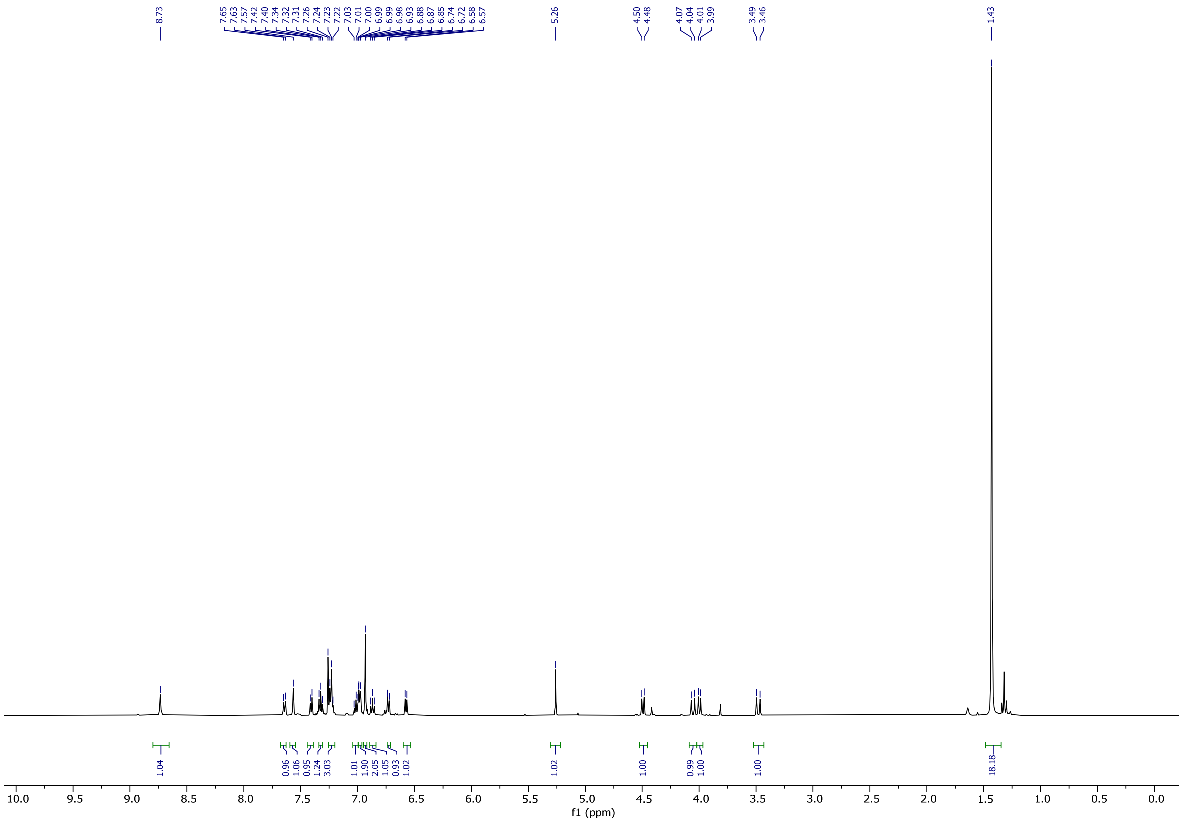

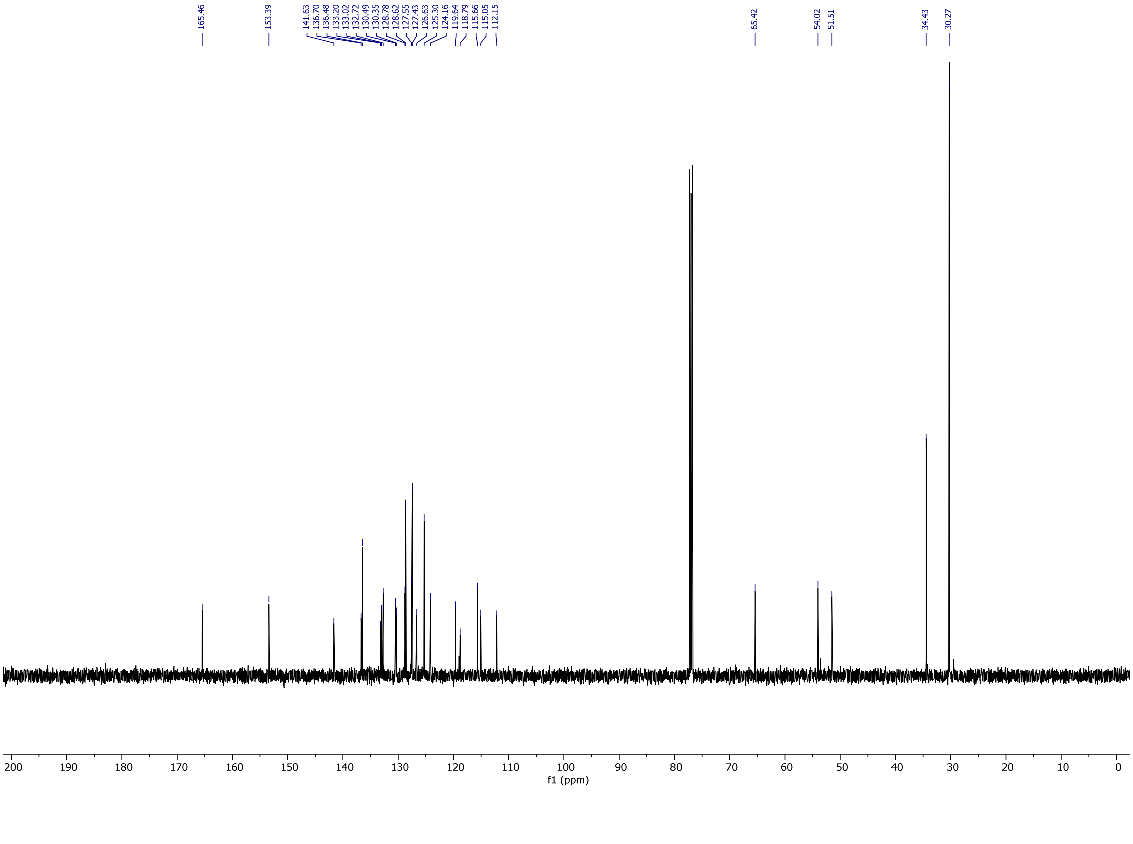

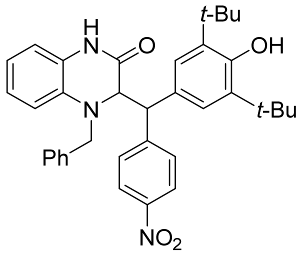

3ah
^{1}H NMR (CDCl$_3$, 300 MHz)
^{13}C {^{1}H}-NMR (CDCl$_3$, 75 MHz)

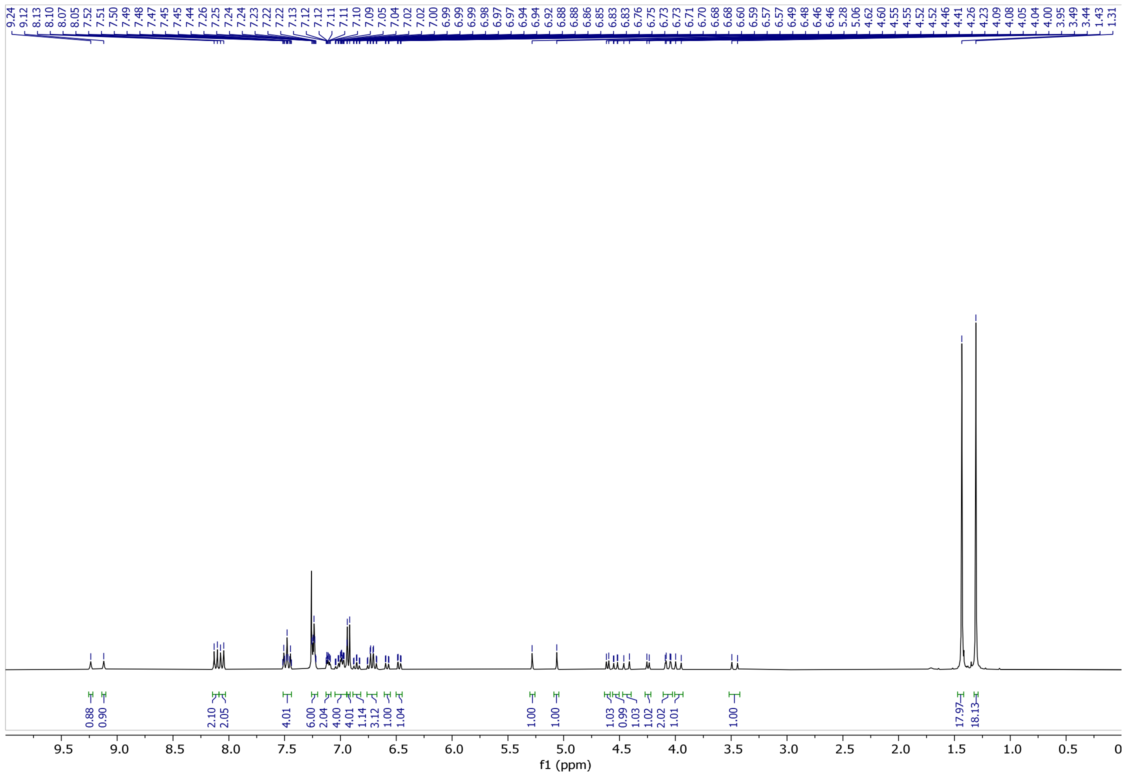

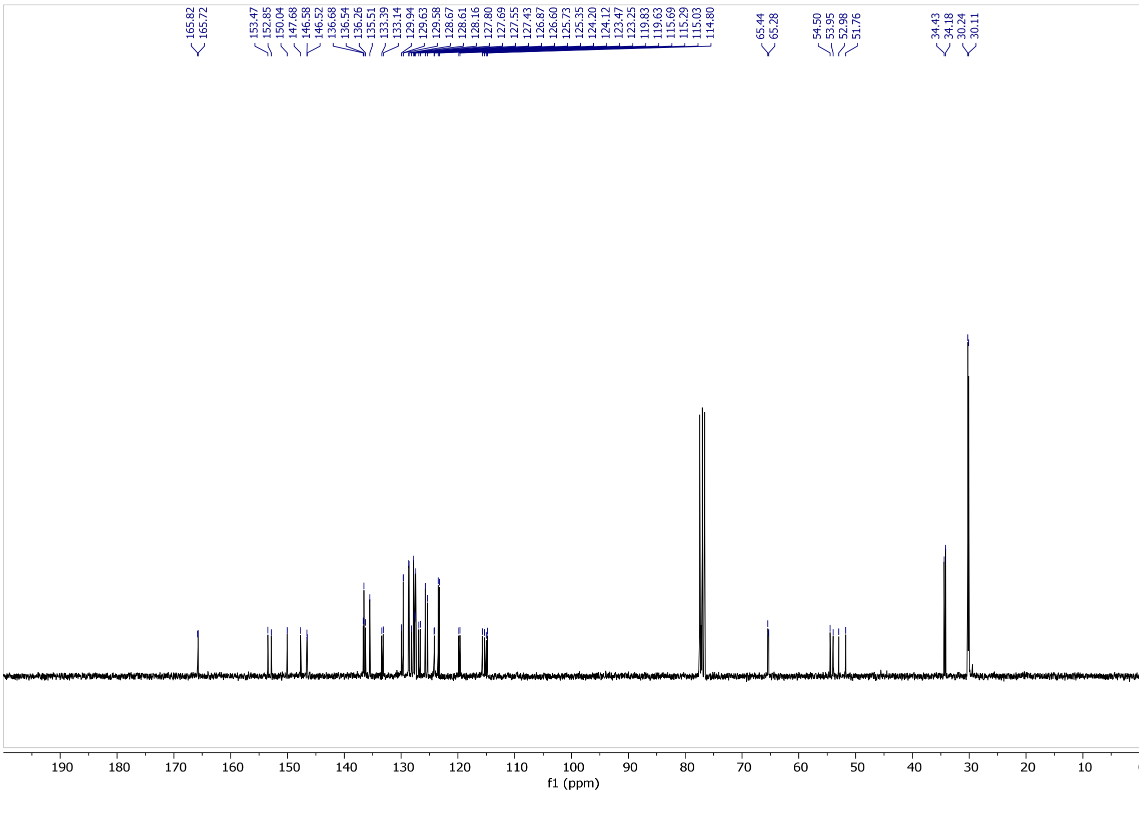

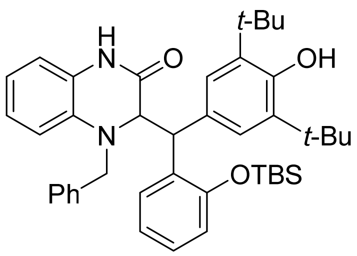

3ai'

^{1}H NMR ($CDCl_3$, 300 MHz)

^{13}C {^{1}H}-NMR ($CDCl_3$, 75 MHz)

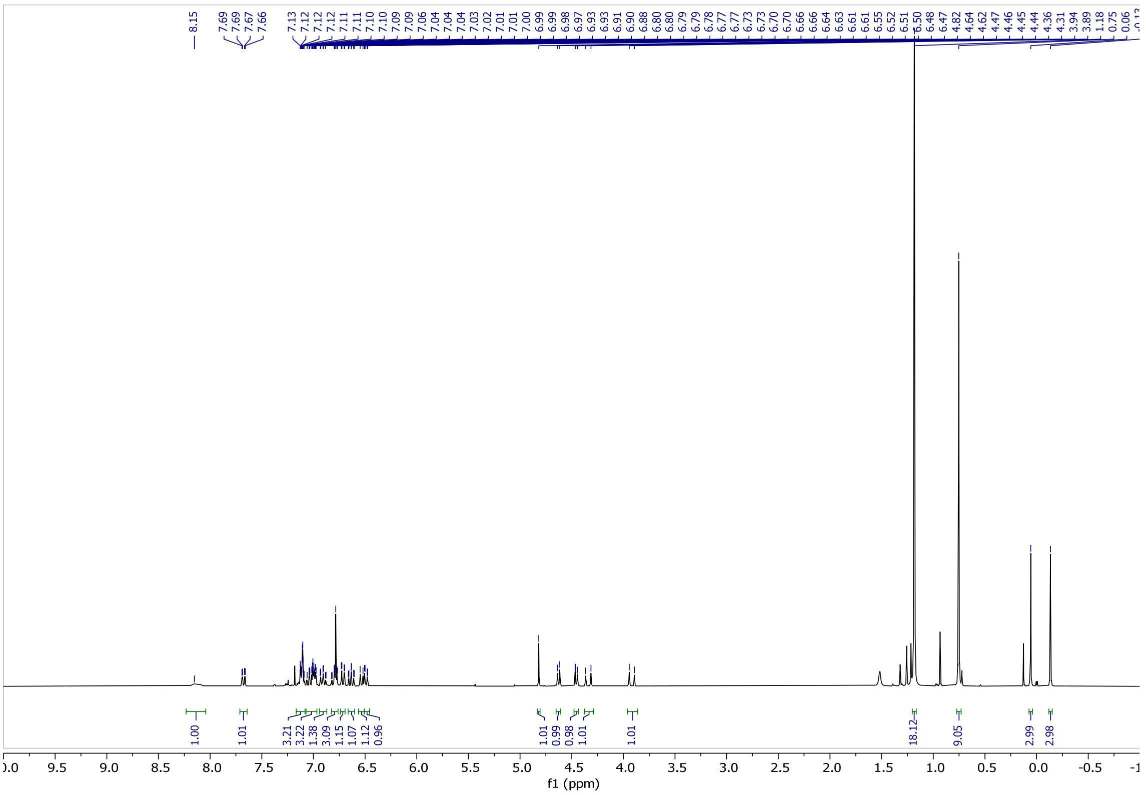

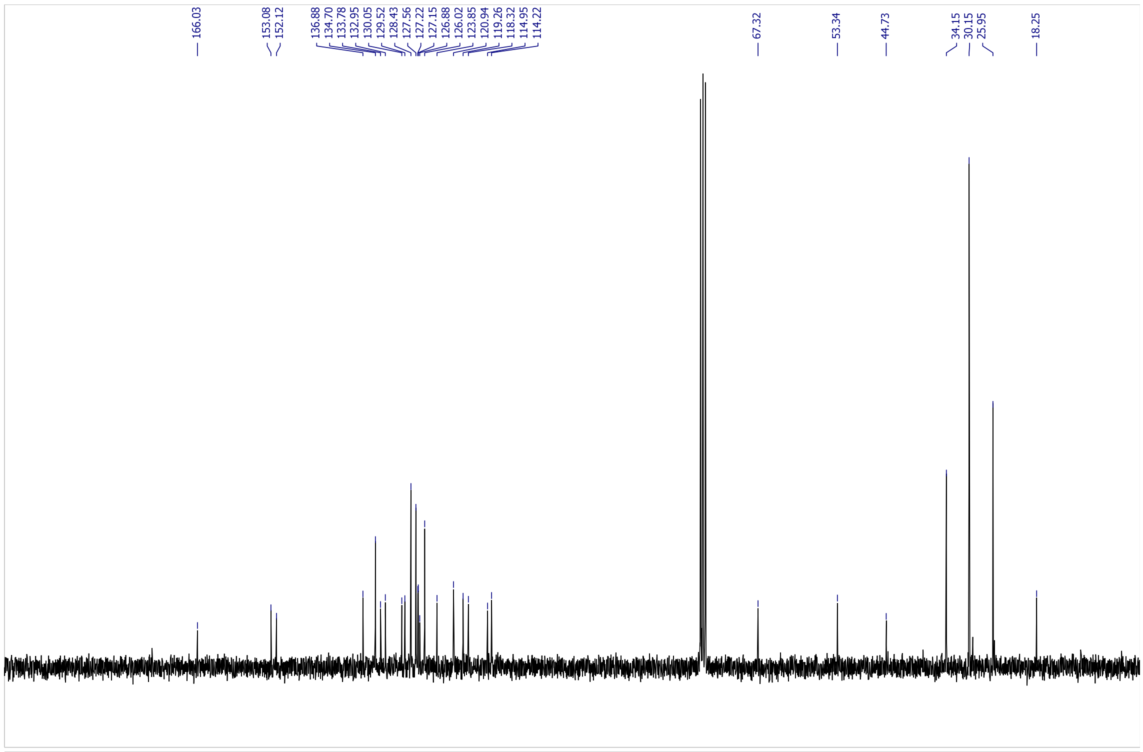

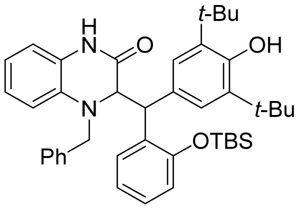

3ai''
^{1}H NMR ($CDCl_3$, 300 MHz)
$^{13}C\{^{1}H\}$-NMR ($CDCl_3$, 75 MHz)

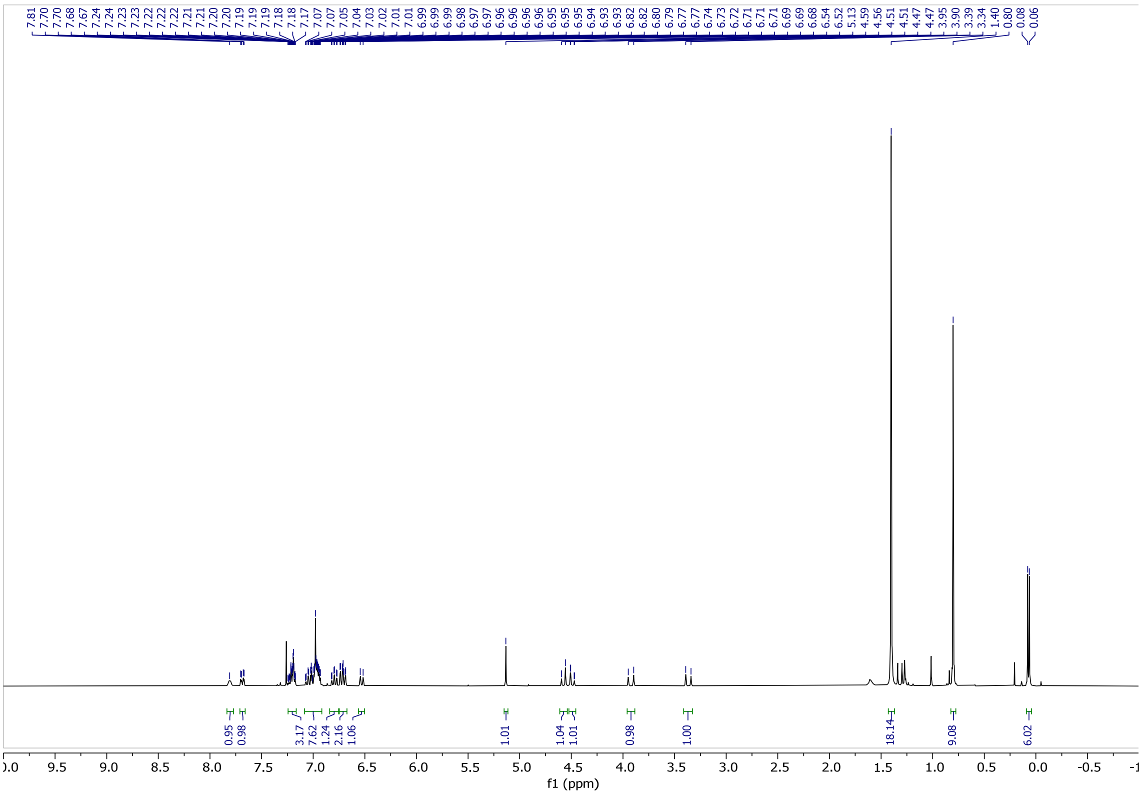

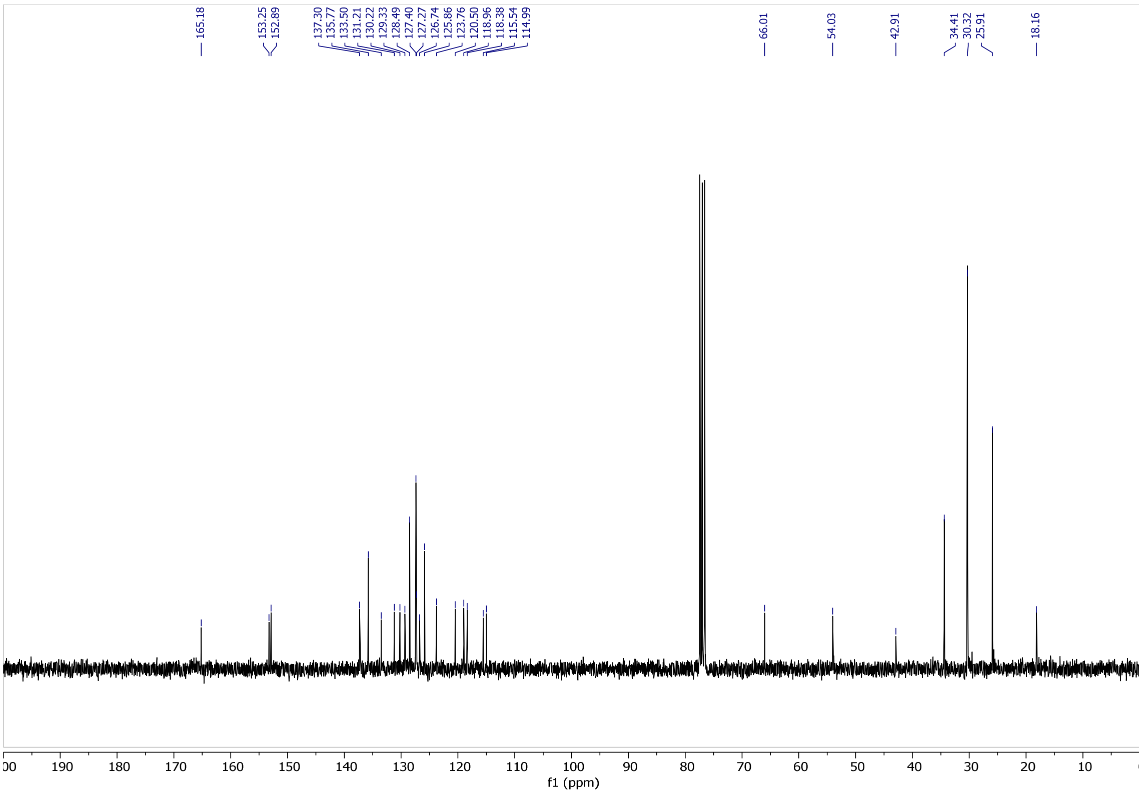

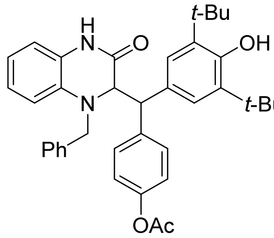

3aj'
^{1}H NMR (CDCl$_3$, 500 MHz)
^{13}C {^{1}H}-NMR (CDCl$_3$, 126 MHz)

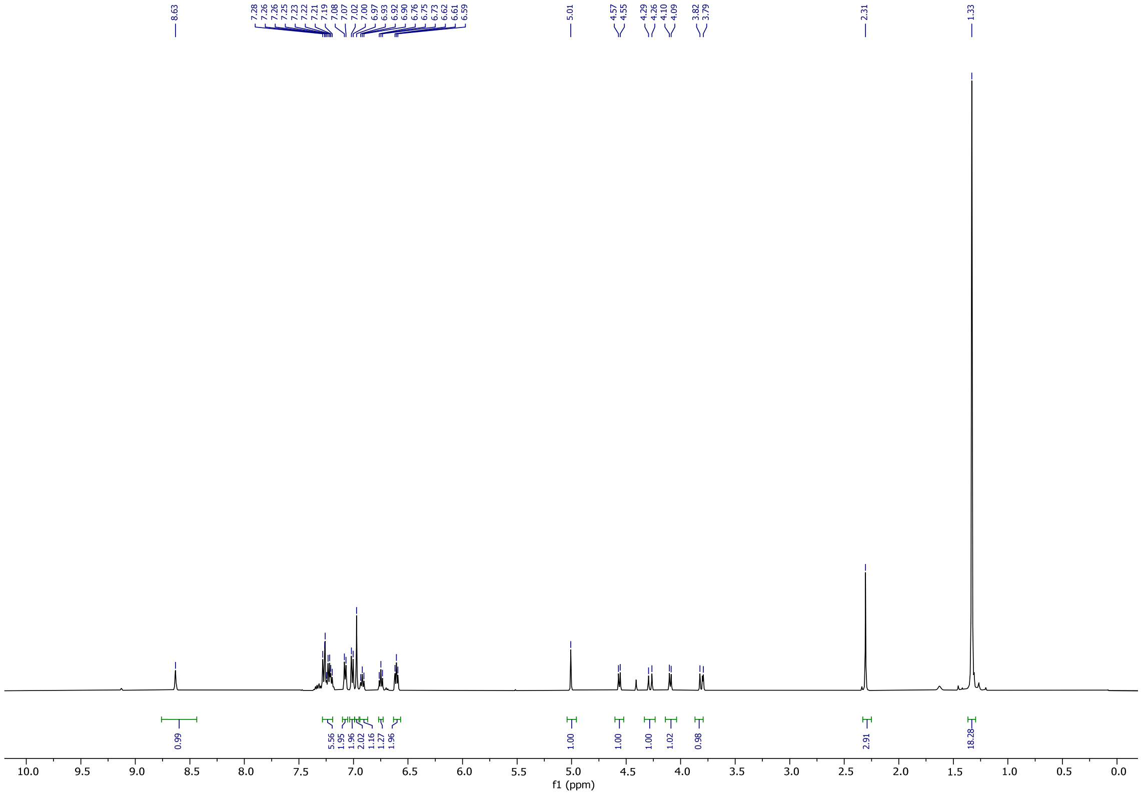

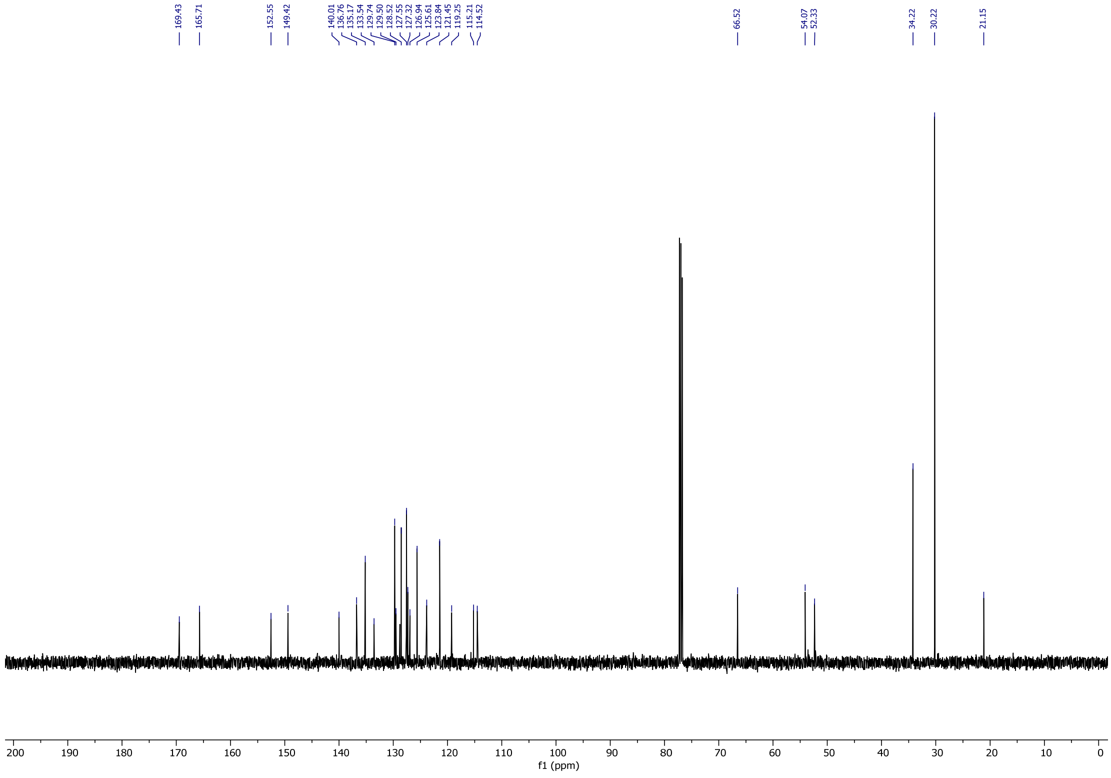

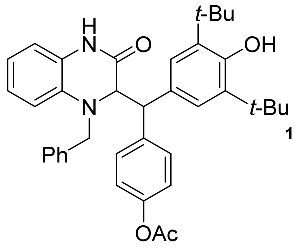

3aj''

^{1}H NMR ($CDCl_3$, 500 MHz)

^{13}C {^{1}H}-NMR ($CDCl_3$, 126 MHz)

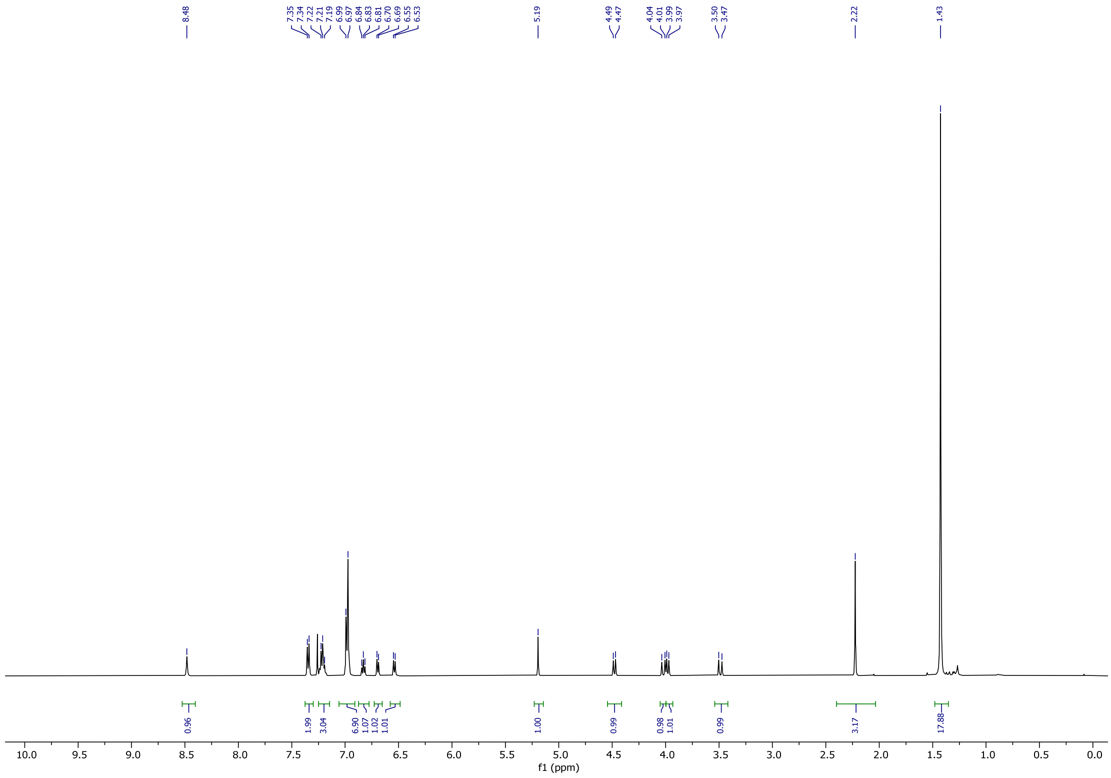

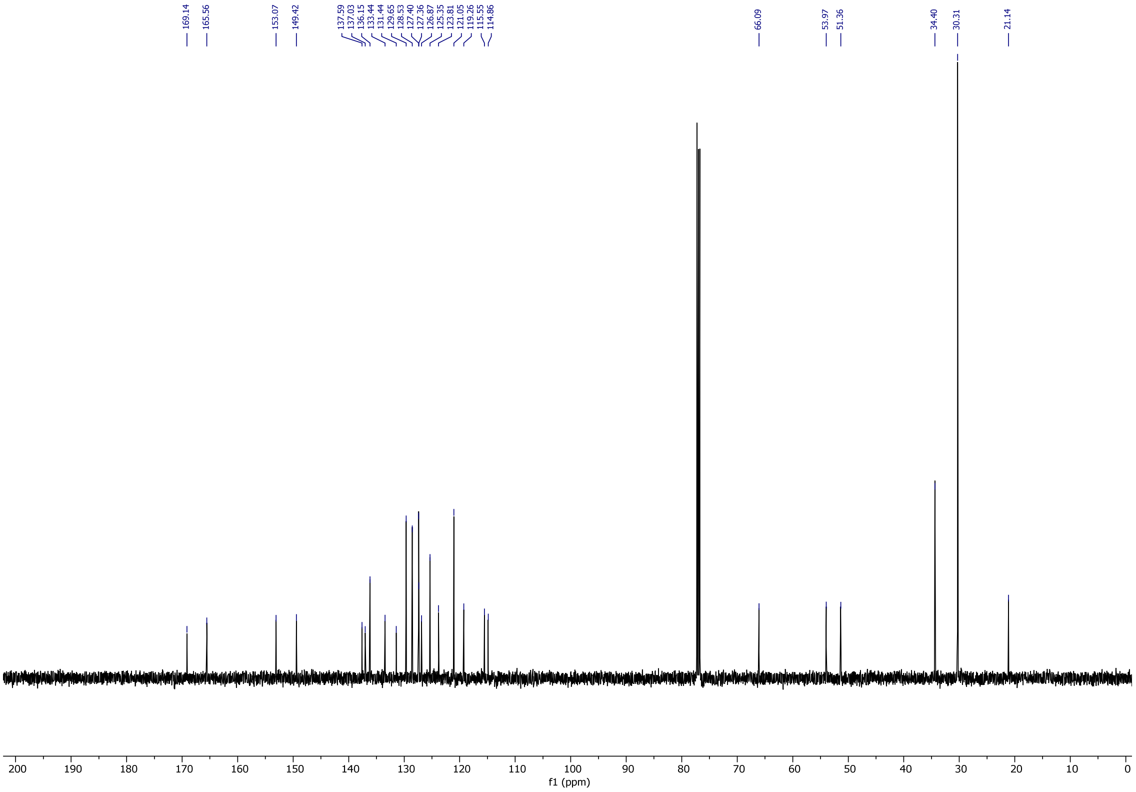

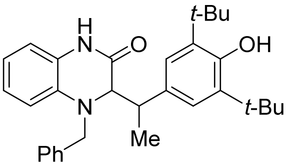

3ak
^{1}H NMR ($CDCl_3$, 500 MHz)
^{13}C {^{1}H}-NMR ($CDCl_3$, 126 MHz)

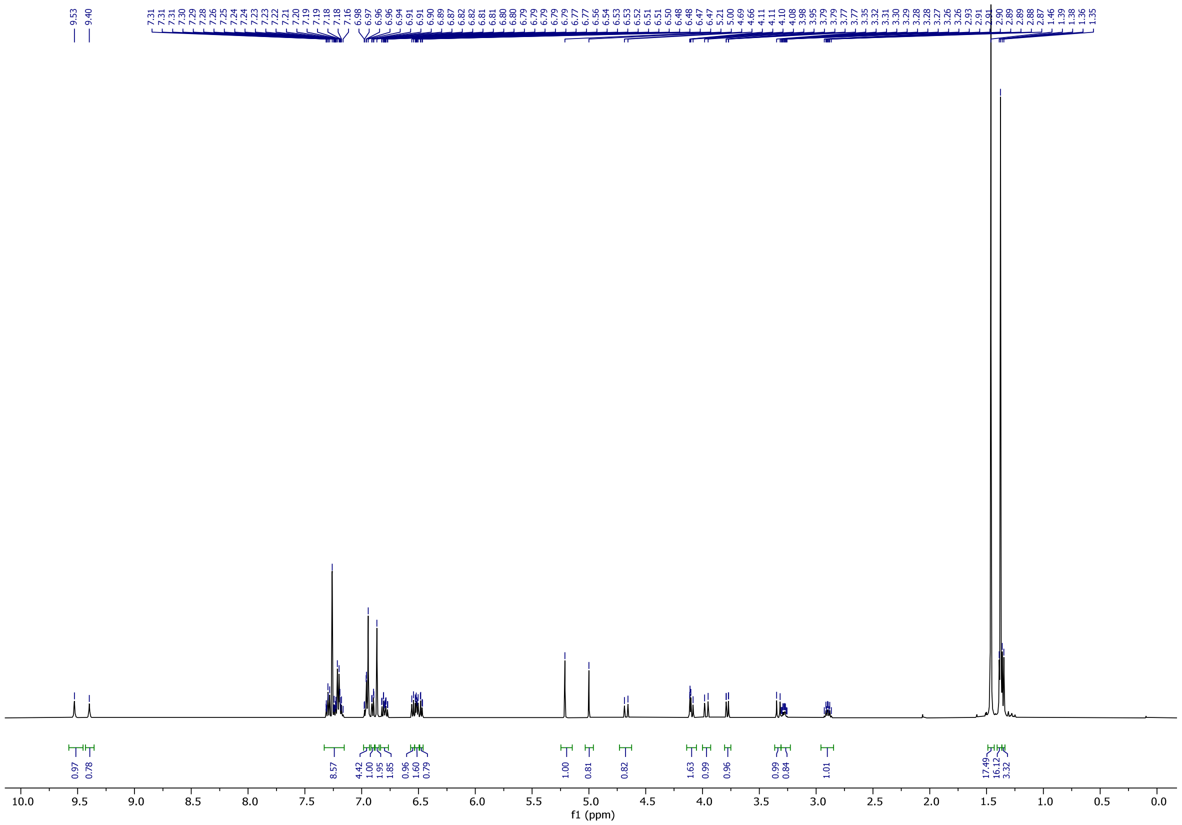

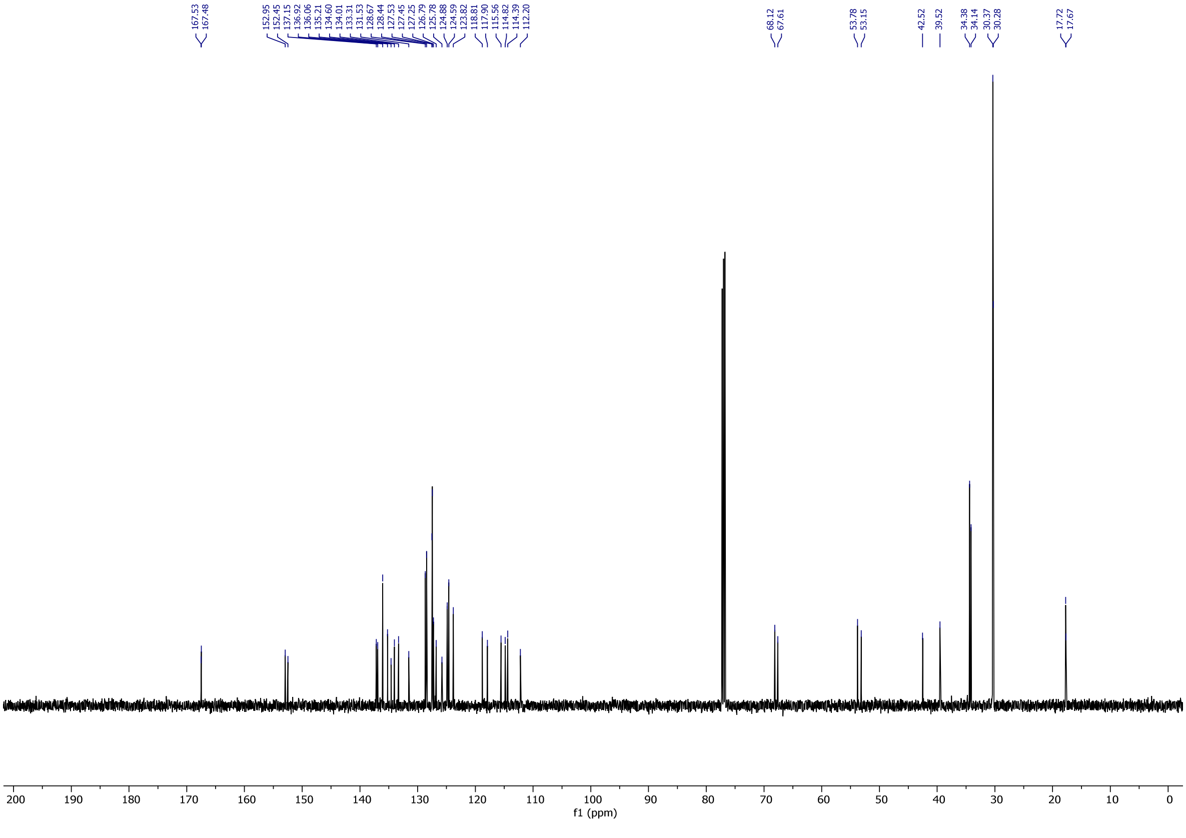

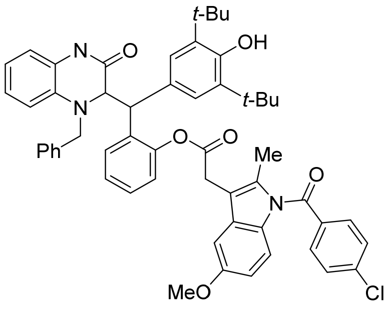

3al'
^{1}H NMR ($CDCl_3$, 300 MHz)
$^{13}C\{^{1}H\}$-NMR ($CDCl_3$, 75 MHz)

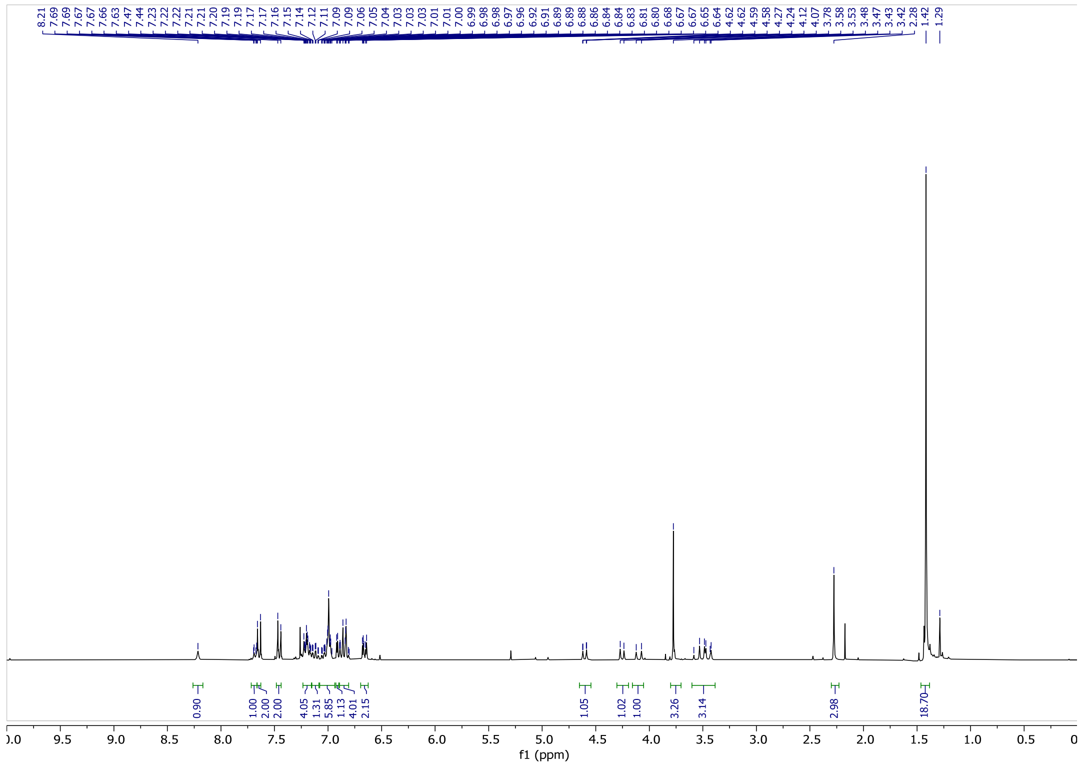

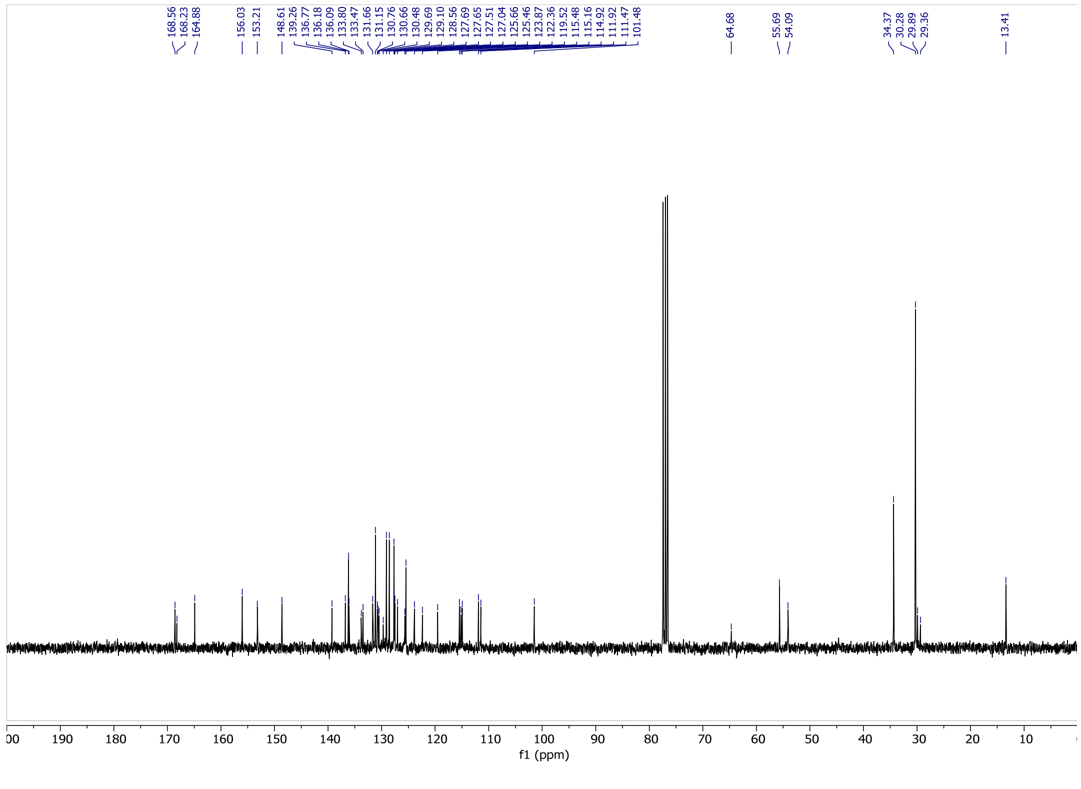

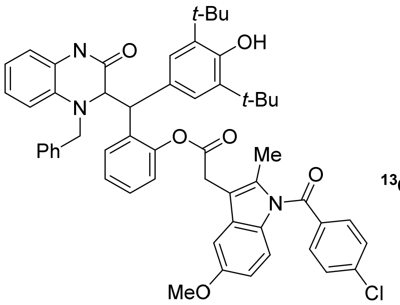

3al''

^{1}H NMR ($CDCl_3$, 300 MHz)

^{13}C{1H}-NMR ($CDCl_3$, 75 MHz)

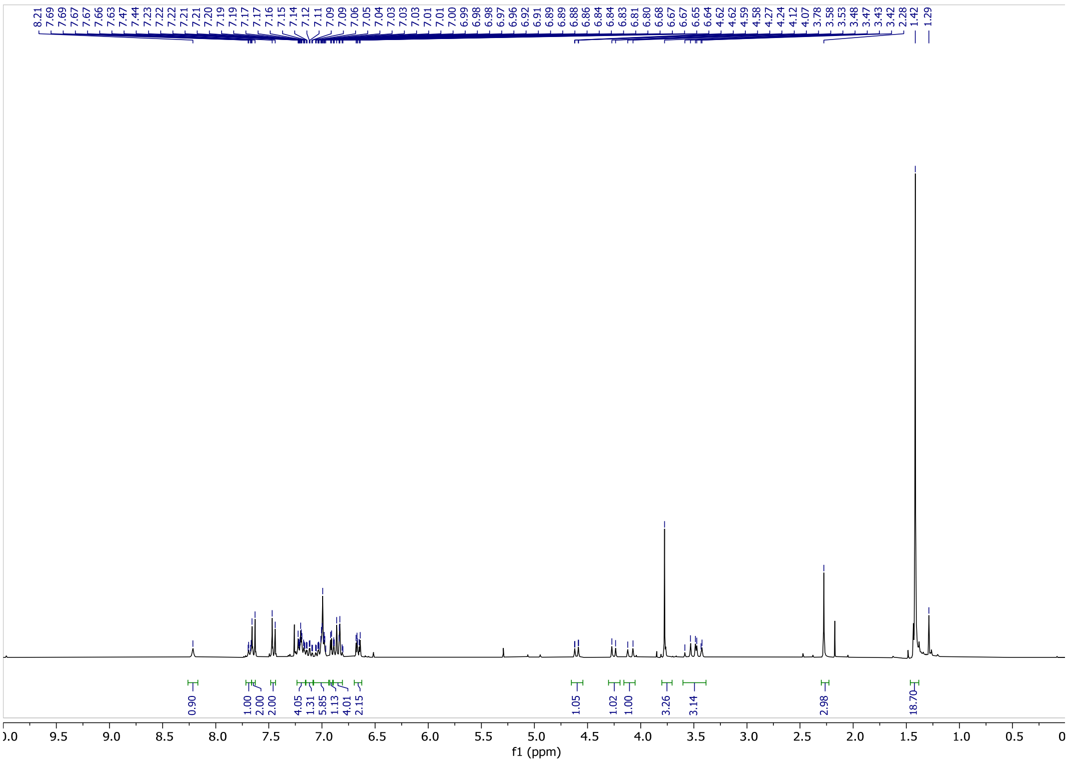

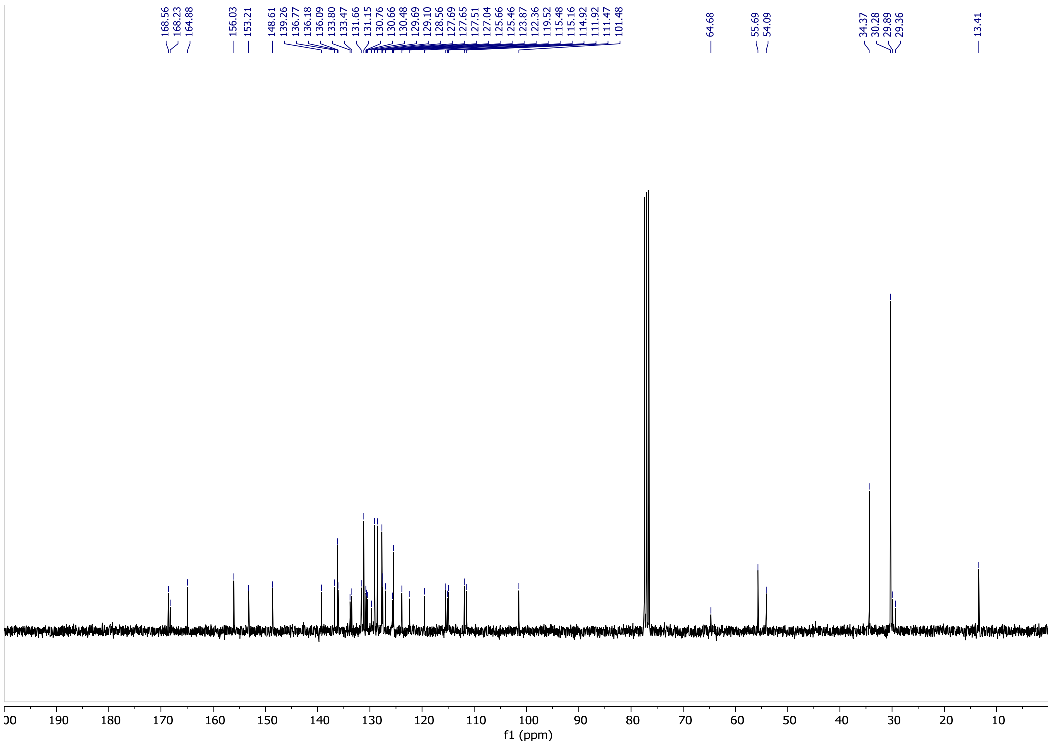

Supplement: Supplementary file 1 — gg2c00064_si_001.pdf [file gg2c00064_si_001.pdf]
